# Resolving phylogenetic conflicts in Pandanales: the dual roles of gene flow and whole-genome duplication

**DOI:** 10.3389/fpls.2025.1511582

**Published:** 2025-02-24

**Authors:** Tian Shi, Jian He

**Affiliations:** State Key Laboratory of Efficient Production of Forest Resources, Beijing Forestry University, Beijing, China

**Keywords:** Pandanales phylogeny, gene flow, whole-genome duplication, gene tree-species tree discordance, phylogenomic

## Abstract

**Introduction:**

Accurate phylogenetic reconstruction is crucial for understanding evolutionary relationships and biodiversity. Despite advances in molecular systematics, the relationships within Pandanales—which include Cyclanthaceae, Pandanaceae, Stemonaceae, Triuridaceae, and Velloziaceae—remain unresolved. This study aims to clarify these relationships by analyzing transcriptomic and genomic data from these families.

**Methods:**

We analyzed transcriptomic and genomic data from 20 samples representing all five families of Pandanales. Our approach involved assembling 2,668 single-copy orthologous genes (SCOGs) and conducting phylogenetic analyses using both coalescent- and concatenation-based methods, alongside plastid genome data. Additionally, we employed HyDe for gene flow analysis and conducted coalescent simulations and QuIBL analyses to explore sources of phylogenetic conflict. We also investigated whole-genome duplication (WGD) events within Pandanales.

**Results:**

The phylogenetic analyses produced strongly supported but topologically incongruent trees. Our gene flow analysis suggested that the concatenation-based topology likely reflects the true evolutionary history of Pandanales. We identified two significant ancient gene flow events: one between Velloziaceae and Triuridaceae, and another between Triuridaceae and the C-P clade (Cyclanthaceae + Pandanaceae). Furthermore, we detected five whole-genome duplication (WGD) events, including two that occurred before the Cretaceous–Paleogene boundary in Stemonaceae and Pandanaceae, one in Triuridaceae during the mid-Paleogene, and two within Velloziaceae near the Paleogene–Neogene boundary.

**Discussion:**

Our findings indicate that gene flow, rather than incomplete lineage sorting, is the primary source of phylogenetic conflict at certain nodes within Pandanales. The identified WGD events likely played a significant role in facilitating adaptation and diversification under changing environmental conditions. These results not only resolve long-standing phylogenetic conflicts but also enhance our understanding of the mechanisms driving plant diversification within this order.

## 1 Introduction

Accurate phylogenetic reconstruction is essential for understanding evolutionary relationships and plays a crucial role in elucidating the complex processes underlying biological diversity and adaptation (Irisarri et al., 2017; Baker et al., 2022; Zuntini et al., 2024). Advances in DNA sequencing technologies have greatly enhanced molecular phylogenetic studies across a broad range of organisms, including plants, animals, and fungi (The Angiosperm Phylogeny Group, 1998; 2003; 2009; 2016; Upham et al., 2019).

However, despite nearly three decades of research in molecular systematics, the evolutionary relationships of certain taxa remain unresolved (Blair and Murphy, 2011; Baker et al., 2022). The persistent ambiguity in these relationships may result not only from technical issues in tree-building methods (e.g., the use of inappropriate substitution models or incorrect orthology inference; Blair and Murphy, 2011) but also from biological processes such as WGD, which significantly increase gene copy numbers and complicate orthology inference (Freeling, 2009). Additionally, the use of multiple gene markers with conflicting signals may contribute to phylogenetic discrepancies. These conflicts are generally thought to arise from two key biological processes: incomplete lineage sorting (ILS) and gene flow, both of which add significant complexity to the interpretation of plant evolutionary relationships (He et al., 2022; Stull et al., 2023).

Pandanales is a monocot order whose evolutionary relationships remain a subject of ongoing debate. It comprises five distinct families—Cyclanthaceae, Pandanaceae, Stemonaceae, Triuridaceae, and Velloziaceae. Despite containing only 34 genera and approximately 1,300 species, Pandanales exhibits remarkable diversity in growth forms, ranging from large, arborescent species of Pandanus, to herbaceous climbers such as Stemona, and inconspicuous, achlorophyllous, mycoheterotrophic herbs in Triuridaceae (Dahlgren and Clifford, 1982; Dahlgren et al., 1985). This diversity extends to reproductive structures: Triuridaceae features unusual apocarpous female flora, while some species of Stemonaceae and Pandanaceae possess monocarpellary flowers—traits that are unusual among monocots. Additionally, flowers in Pandanales often exhibit dimerous and tetramerous arrangements, in contrast to the trimerous pattern commonly found in other lilioid monocots (Rudall and Bateman, 2006).

Due to its remarkable morphological diversity, each family in Pandanales was historically classified into different orders based on morphological traits. Originally, Pandanales was thought to consist solely of the family Pandanaceae (Dahlgren et al., 1985; Cronquist, 1988). Cyclanthaceae were placed in Arecales with palms, Stemonaceae in Dioscoreales, Velloziaceae with bromeliads in Poales, and the mycoheterotrophic Triuridaceae was tentatively aligned with taxa now recognized in Alismatales (Dahlgren and Clifford, 1982; Dahlgren et al., 1985). However, molecular systematics has placed these diverse lineages within a single order, Pandanales (Chase et al., 2000; 2006; Caddick et al., 2002; Davis et al., 2004; Grahan et al., 2006; Mennes et al., 2013; The Angiosperm Phylogeny Group, 2009; 2016; Lam et al., 2016; 2018; Soto Gomez et al., 2020; Baker et al., 2022).

However, the phylogenetic relationships within Pandanales remain contentious. Early systematics studies of this order (Chase et al., 2000; 2006; Caddick et al., 2002; Davis et al., 2004; Grahan et al., 2006) were constrained by the use of limited molecular markers (1–2 chloroplast or rDNA fragments), resulting in low resolution and conflicting phylogenetic relationships. Recent studies using a larger number of molecular markers have provided higher-resolution phylogenetic trees. However, significant discrepancies remain. For example, Mennes et al. (2013) combined 18S rDNA, atpA, matR, and nad1 intron datasets to produce a highly resolved phylogenetic tree. Their analysis identified Velloziaceae as the basal lineage of Pandanales, with Triuridaceae and Stemonaceae diverging later. Cyclanthaceae and Pandanaceae formed a stable clade (C-P clade), which was sister to Stemonaceae with moderate support (Bayesian posterior probability = 0.59). A more recent phylogenomic study by Baker et al. (2022), based on 353 nuclear gene markers, found a similar phylogenetic topology. In contrast, studies using complete plastid and mitochondrial genomes (Lam et al., 2016, 2018; Givnish et al., 2018; Soto Gomez et al., 2020) produced phylogenetic trees that differed from Mennes et al. (2013) by positioning the C-P clade as sister to Triuridaceae, though with weak support.

Another intriguing aspect of Pandanales is the occurrence of WGD events. As previously noted, WGD events can significantly increase gene copy numbers, complicating the inference of orthologous genes and potentially leading to phylogenetic conflicts (Freeling, 2009). In a transcriptome-based study, One KP (2019) identified WGD events in three families of Pandanales—Velloziaceae, Stemonaceae, and Pandanaceae—while no such events were detected in Cyclanthaceae. However, this study did not include Triuridaceae, a family with distinct ecological and morphological characteristics, as discussed earlier. Whether Triuridaceae has undergone WGD remains an open and intriguing question.

Building on these findings, this study has two primary aims. The first is to resolve the precise phylogenetic relationships among the five families within Pandanales, with a particular focus on the accurate placement of the C-P clade. The second aim is to assess the impact of various biological processes—such as ILS, gene flow, and WGD—on the phylogeny and evolutionary history of Pandanales.

## 2 Materials and methods

### 2.1 Data sources and sequence processing

In our study, we obtained genomic data from 17 samples representing all five families of the order Pandanales, covering 17 species across 9 genera. Additionally, three outgroup samples from Dioscoreales, the sister group to Pandanales, were included. Of the 20 total samples, raw transcriptomic sequencing reads for 19 were retrieved from the NCBI Sequence Read Archive (SRA) database (https://www.ncbi.nlm.nih.gov/sra). For Acanthochlamys bracteata, whose entire genome has been sequenced, we directly downloaded all protein-coding DNA sequences (Xu et al., 2022; download from https://ngdc.cncb.ac.cn/gwh/Assembly/20709/show). Further details are provided in **Supplementary Table S1**. Additionally, we downloaded 12 complete chloroplast genome sequences from Pandanales species available in the NCBI database, covering all five families (representing 12 species and 8 genera). Three samples from Dioscoreales were included as outgroups (**Supplementary Table S1**).

The 19 raw transcriptomic sequencing SRA files were extracted using sratoolkit version 2.9.2 (https://ncbi.github.io/sra-tools/). Low-quality bases from the sequencing reads were trimmed using Trimmomatic v.0.39 (Bolger et al., 2014) with the parameters of “LEADING: 3, TRAILING: 3, SLIDINGWINDOW: 4:15, HEADCROP: 8, MINLEN: 36”. Organellar clean reads were identified and removed through bwa-mem v.0.7.17 analysis (using default parameters) against the 15 plastid genomes (**Supplementary Table S1**) and a mitochondrial genome of *Stemona sessilifolia* (PP692484). The remaining clean RNA-Seq reads were *de novo* assembled using Trinity v.2.15.1 (Grabherr et al., 2011) with default parameters. The longest isoform for each gene was selected using the Trinity script “get_longest_isoform_seq_per_trinity_gene.pl.” Protein-coding sequences were identified and translated into amino acid sequences using TransDecoder v3.0.1 (https://github.com/TransDecoder/). These 19 samples’ protein-coding sequences were analyzed alongside *Acanthochlamys bracteata*, for which we directly downloaded all protein-coding sequences (Xu et al., 2022). Finally, redundancy in the assembled sequences was reduced using CD-HIT v4.6.2 (Li and Godzik, 2006).

### 2.2 Nuclear ortholog identification, filter and alignment

The protein-coding contigs from the 20 samples underwent an all-versus-all BLAST search using Proteinortho v6.0.10 (Lechner et al., 2011) with an E-value threshold set at 1×10^−5^. A Python script (https://github.com/Jhe1004/Get_SCOG_from_Proteinortho) was employed to isolate SCOGs from the Proteinortho output, ensuring that each SCOG was present in at least 12 samples. A total of 2,668 SCOGs were identified.

Orthologous sequence identification through BLAST can be complicated by issues like mis-assembly, deep coalescence, or frame shifts (Yang and Smith, 2014). In some cases, sequences can display unexpectedly long branches when incorrectly grouped with other orthologs. To mitigate these issues, TreeShrink v1.3.9 (Mai et al., 2017) was employed to examine and remove excessively long branches in the gene trees. We first aligned the SCOG sequences using MAFFT v7.475 (Katoh and Standley, 2013) with default settings, and ambiguities were resolved by excising uncertain regions with a Python script (https://github.com/Jhe1004/DelMissingSite), applying a 20% “stripping threshold” as described by Duvall et al. (2020). Gene trees were then generated using RAxML v8.2.12 (Stamatakis, 2014) with the GTR+G model. Sequences flagged as suspicious by TreeShrink v1.3.9 (Mai et al., 2017) were pruned from the SCOG alignments using default parameters.

### 2.3 Phylogenetic analysis

We used both coalescent-based and concatenation-based methods to infer phylogenetic relationships from the 2,668 SCOGs. For the coalescent-based method, we employed a two-step summary approach. In the first step, individual gene trees were generated for each SCOG using RAxML v8.2.12 (Stamatakis, 2014) with the GTR+G model. In the second step, all 2,668 gene trees were combined to reconstruct the coalescent tree using ASTRAL v.5.6.3 (Zhang et al., 2018) with default parameters.

For the concatenation-based analysis, all 2,668 SCOGs were merged into a single super-alignment using Geneious Prime’s “Concatenate Sequences or Alignments” function (Kearse et al., 2012). Given that evolutionary rates often vary significantly across the first, second, and third codon positions, we extracted each of these positions separately from the super-alignment to facilitate data partitioning. Phylogenetic reconstruction was then performed using RAxML v8.2.12 (Stamatakis, 2014), applying the GTR+G substitution model (as specified in the RAxML manual) with 100 bootstrap replicates. Since the concatenation-based tree was more consistent with the results of gene flow analysis (detailed results will be described in the Results section), we used this tree as the species tree for subsequent analyses.

Although our transcriptome dataset covered all families within Pandanales, its taxonomic representation at the genus level was limited, including only 9 out of 34 genera. To achieve more comprehensive taxonomic sampling and to further test the monophyly of each family, we incorporated 12 additional samples from the Kew Tree of Life project (https://treeoflife.kew.org/tree-of-life/search-name/?order=Pandanales), which provides direct access to the SCOGs for these samples through their website. These SCOGs were generated by target sequence capture using the universal Angiosperms353 probe set, as described in Baker et al. (2022). On average, each sample contained 335 SCOGs. Together with our transcriptome-derived samples, the dataset included a total of 30 samples, representing 29 species and covering 19 out of 34 genera within Pandanales. These 30 samples, along with three outgroup samples, were processed following the same protocols as described for the transcriptome dataset (Methods 2.2 and 2.3). Finally, both concatenation-based and coalescent-based phylogenetic trees were reconstructed. For downstream analyses, we continued to focus on the transcriptome dataset due to its notably higher data quality.

For the construction of the chloroplast genome phylogenetic tree, we first extracted the protein-coding sequences from the genomes. Sequence alignment was performed using MAFFT v7.221 (Katoh and Standley, 2013). The aligned sequences were then concatenated, and phylogenetic analysis was conducted using maximum likelihood (ML) with RAxML v8.2.12 (Stamatakis, 2014), applying the GTR+G model with 100 bootstrap replicates.

### 2.4 Divergence time estimation

To estimate divergence times within Pandanales, we first filtered the SCOGs to retain only those that were consistent with the species tree topology (concatenation-based tree). This step was necessary because we identified substantial ancient gene flow between families within Pandanales (see Results, section 3.5), which caused significant topological conflicts among the individual gene trees. As a result, the divergence times inferred from some SCOGs were expected to differ significantly from those of the species tree. To mitigate the impact of such conflicts on divergence time estimation, we selected the SCOGs least affected by ancient gene flow. A total of six SCOGs were retained and concatenated for further analysis. Divergence time estimation was conducted in BEAST v2.7.7 (Bouckaert et al., 2014), applying the GTR+G substitution model with relaxed clock priors (Drummond et al., 2006) to account for rate variation among lineages.

Three age constraints were applied during the dating analysis. The first constraint, based on inflorescences fossils of *Mabelia* (Triuridaceae) from the Turonian period (93.5–89.3 Mya; Gandolfo et al., 2002), was a lognormal distribution with a minimum age of 89.3 Mya (95% range = 89.6–111 Mya) for the stem of Triuridaceae. The second constraint, a lognormal distribution with a minimum age of 79 Mya (95% range = 79.3–100 Mya), was applied to the stem of Pandanaceae. This constraint is based on the minimum age of *Pandanites* leaf fossils from Austria, dated to 79 Mya (Kvaček and Herman, 2004). Additionally, a secondary calibration for the stem age of Pandanales was applied, based on a previously estimated age of 106 Mya (Mennes et al., 2013; Soto Gomez et al., 2020), using a normal distribution (95% range = 97.8–114 Mya).

We ran the Markov chain Monte Carlo (MCMC) for 2×10^8^ generations, sampling every 1000 generations and discarding the first 25% as burn-in. To assess the convergence of the MCMC chains, we checked the effective sample size (ESS) for all parameters using Tracer v1.7 (Drummond and Rambaut, 2007), ensuring that ESS values exceeded 200. The maximum clade credibility tree was summarized using TreeAnnotator v2.7.7 (Drummond and Rambaut, 2007).

### 2.5 Detecting whole-genome duplication in the Pandanales

To investigate potential WGD events within Pandanales, we developed a novel detection method. This approach not only determines whether individual genomes have experienced WGD events throughout their evolutionary histories but also estimates the approximate timing of these events.

Our method is based on gene tree dating; however, the construction of these gene trees differs from conventional approaches that rely solely on low-copy orthologous genes. Instead, we include both the orthologs shared among species and their associated paralogs within each genome, which are derived from gene duplication events. In this framework, the leaves of the gene trees represent paralogous genes rather than entire species. We then estimate divergence times at each node, focusing specifically on the timing of paralog origin events.

For instance, consider a gene tree in which species “A” contains four paralogous genes. These genes might exhibit three divergence events at approximately 10 million years ago (Ma), 10.5 Ma, and 20 Ma. By analyzing 1,000 such gene trees and compiling a distribution of their paralog divergence times, one could observe that most paralogs in species A cluster around 10 Ma. This pattern indicates a likely WGD event in species A around 10 Ma. Thus, our method provides an effective means of detecting and dating WGD events.

In this study, the first step involved obtaining the specialized gene trees required for WGD detection. As described in Section 2.2, we used Proteinortho v6.0.10 (Lechner et al., 2011) to cluster homologous genes among the 20 sampled genomes. However, whereas Section 2.2 focused on retaining low-copy gene clusters (i.e., those lacking paralogs), we applied the opposite criterion here: only gene clusters containing paralogous genes were retained. For each retained cluster, we constructed a maximum likelihood (ML) phylogeny using RAxML v8.2.12 (Stamatakis, 2014) with the GTR+G substitution model. Divergence times for all internal nodes in each gene tree were then estimated using TreePL v1.0 (Smith and O’Meara, 2012), with the root age of Pandanales constrained by our previously established dating results (Method 2.4).

Next, we used a Python script to extract the estimated paralog origin times from each gene tree in every sample. These divergence times were subsequently used to plot a frequency distribution. We assumed that a true WGD event would result in a substantial clustering of paralog origin times around the event. However, due to computational uncertainties and inherent biological variation, these times are expected to approximately follow a normal distribution (Vanneste et al., 2014). To illustrate this pattern, we fitted Gaussian mixture models to visualize the timing of duplication events. The Gaussian mixture models were implemented using scikit-learn v0.24.1 (Pedregosa et al., 2011). All Python scripts associated with these steps are available at https://doi.org/10.5281/zenodo.13857688.

Additionally, with the availability of chromosome-level genome assembly for *Acanthochlamys bracteata* (Xu et al., 2022; https://ngdc.cncb.ac.cn/gwh/Assembly/20709/show), we employed a whole-genome dot plot approach to detect WGD events. We performed a BLAST search of *Acanthochlamys bracteata* genes against itself, using an E-value cut-off of 1×10^−3^, retaining the top 10 best hits from the BLAST results. The dot plot was then generated using the “Quick Genome Dot Plot” tool in TBtools (Chen et al., 2023).

### 2.6 Detecting gene tree-species tree discordance

To visually represent conflicts among gene trees, we generated cloud-tree plots using Toytree v2.0.5 (Eaton, 2020). To ensure the accuracy of the gene trees, we only included those constructed from SCOGs with sufficient parsimony-informative sites (i.e., >600 sites). Additionally, only gene trees containing no missing taxa were retained, as required by the Toytree software. The remaining 114 gene trees were time-calibrated using TreePL v1.0 (Smith and O’Meara, 2012), with the root age of Pandanales constrained by our previously established dating results (Method 2.4).

To assess discordance between gene trees and the species tree, we used PhyParts v0.0.1 (Smith et al., 2015) to map properly rooted nuclear gene trees onto the concatenation-based species tree. The same set of gene trees selected for the Toytree visualization was used in this analysis. PhyParts summarizes the proportion of gene trees that are concordant or conflicting with each node of the species tree.

### 2.7 Coalescent simulation for tree discordance

Systematic discordances between gene trees and the species tree are likely caused by gene flow and/or incomplete lineage sorting (ILS). We employed two approaches to evaluate the relative contributions of these processes to the observed phylogenetic incongruences.

First, we conducted a coalescent simulation following established methods (Yang et al., 2020; Morales-Briones et al., 2021; Liu et al., 2022; He et al., 2022). The method begins by calculating the tree-to-tree distances (Robinson and Foulds, 1981) between each empirical gene tree and the species tree, followed by plotting the frequency distribution of these distances. Subsequently, under the assumption that all conflicts in the simulated gene trees arise solely from ILS, a large set of gene trees (simulated gene trees) is generated based on the same species tree using the multispecies coalescent (MSC) model. By comparing the two frequency distributions (one derived from empirical gene trees and the other from simulated gene trees), the extent to which the observed discordances can be attributed to ILS is assessed. If the two distributions are highly similar, the conflicts between the empirical gene trees and the species tree are inferred to be primarily due to ILS. Conversely, significant differences between the distributions suggest the involvement of additional factors, such as gene flow.

For this study, we used the same empirical gene trees as in Method 2.6, while we used the “sim.coaltree.sp” function in the R package Phybase v1.5 (Liu and Yu, 2010) to simulate 10,000 gene trees. The tree-to-tree distances (Robinson and Foulds, 1981 were calculated using DendroPy v4.5.2 (Sukumaran and Holder, 2010). After obtaining the distributions of tree-to-tree distances for both empirical and simulated gene trees, we performed a chi-square test. The null hypothesis posits that conflicts between the empirical gene trees and the species tree result solely from ILS. A p-value below 0.05 leads to rejection of this hypothesis.

Next, we also utilized QuIBL software to evaluate the relative contributions of ILS and gene flow. According to the coalescent theory proposed by Edelman et al. (2019), the internal branches of rooted gene trees for a set of three taxa (a “triplet”) can be modeled as a mixture of two distributions: one representing ILS and the other representing gene flow or speciation events. Therefore, the estimated mixing proportions (π_1_ for ILS and π_2_ for gene flow/speciation, where π_1_ + π_2_ = 1) reflect the relative contributions of each process in generating the gene trees.

For each triplet, QuIBL calculates the proportions of gene trees supporting each of the three possible topologies. It then employs Expectation-Maximization to estimate the mixing proportions (π_1_, π_2_) and other relevant parameters, while also calculating the Bayesian Information Criterion (BIC) scores for both ILS-only and gene flow models. In cases where a topology is discordant with the species tree, high π_2_ values suggest the potential involvement of gene flow.

For this analysis, the same set of gene trees as described in Method 2.6 was used. QuIBL was run on each triplet individually with the default parameters (numdistributions: 2; likelihoodthresh: 0.01; numsteps: 50; gradascentscalar: 0.5). Following Edelman et al. (2019), we applied a ΔBIC < -30 threshold to identify significant gene flow events. These significant gene flow events were then visualized in a heat map, highlighting the π_2_ values.

### 2.8 Gene flow detection

We utilized the HyDe software (Blischak et al., 2018) to detect gene flow events in Pandanales, applying a coalescent-based model to estimate gene flow signals using phylogenetic invariants. Similar to Patterson’s D-statistic (Green et al., 2010; Patterson et al., 2012), HyDe operates on a rooted 4-taxon tree “(((P1, H), P2), O),” where “O” represents the outgroup. The gene flow model assumes that a proportion, γ, of the genetic material in “H” has been introgressed from “P2”. The null hypothesis, which assumes no gene flow, posits that γ equals 0. HyDe uses a Z-test to assess whether γ significantly deviates from 0, with a higher Z-score providing stronger evidence for gene flow.

In our analysis, we applied HyDe to a concatenated super-alignment dataset comprising 17 Pandanales samples, with *Aletris farinosa* designated as the outgroup. We tested all possible combinations of the ingroup samples, with results filtered according to default thresholds (0 < γ < 1, p < 0.05, Z > 1) to identify significant gene flow events. These significant events were then visualized.

We employed a custom visualization script (details and usage available at [https://github.com/Jhe1004/VisualHyde]) to facilitate the clear display of detected gene flow events. This tool enabled an intuitive examination of gene flow patterns across the dataset, providing a straightforward approach to identify and interpret gene flow signals between taxa.

Beyond HyDe, we also used PhyloNet v3.8.0 (Wen et al., 2018) to investigate the phylogenetic networks of Pandanales. In our analysis, we employed a pseudolikelihood-based method in PhyloNet to detect gene flow events using the multispecies coalescent model. The gene trees used in PhyloNet were the same as those described in Method 2.6. We allowed for up to ten gene flow events and ran five independent replicates. The optimal number of gene flow events was determined by plotting the pseudolikelihood scores. When the score first reaches a local maximum or the rate of increase slows significantly, the optimal number of gene flow events is identified.

## 3 Results

### 3.1 Transcriptome and plastid genome characteristics

Transcriptome data for 19 samples were obtained, with high-quality paired-end reads ranging from 10 to 55 million (**Supplementary Table S1**). The number of reads mapped to organelle genomes and subsequently removed from the data ranged from 20,908 (*Pandanus tectorius*) to 1,779,340 (*Pandanus odorifer*). The final count of protein-coding sequences across the 20 samples (19 transcriptome and 1 whole-genome sequencing data) ranged from 16,204 in *Lacandonia schismatica* to 34,500 in *Aletris farinosa*. N50 values, representing the median contig length, ranged from 918 bp in *Croomia pauciflora* to 1,863 bp in *Acanthochlamys bracteata*, with an average of 1,191.6 bp (**Supplementary Table S1**).

Except for *Sciaphila densiflora*, which has a small plastid genome of 21,485 bp (as a parasitic plant), the plastid genomes of the other 14 species are around 150,000 bp. These plastids typically contain about 78 protein-coding genes, with *Sciaphila densiflora* having only 18. Additionally, *Sciaphila densiflora* has only 6 tRNA genes, compared to approximately 30 in the other species. The rRNA gene content is consistent across all species, with four rRNA genes per species (**Supplementary Table S1**).

### 3.2 Identification and filtering of single copy orthologous genes

After clustering the sequences, we identified 2,668 SCOGs (**Supplementary Figure S1**; **Supplementary Table S2**). Using TreeShrink, we removed 1,344 unusually long branches from 891 SCOGs. The alignment lengths of these SCOGs ranged from 243 to 5,889 bp, with an average length of 1,153.1 bp. The number of species represented in each SCOG ranged from 12 to 20, with an average of 17.2. The number of SCOGs contained within each species ranged from 1,781 in *Burmannia biflora* to 2,507 in *Pandanus tectorius*.

### 3.3 Phylogenetic inference and divergence time estimation

**Figure 1:**
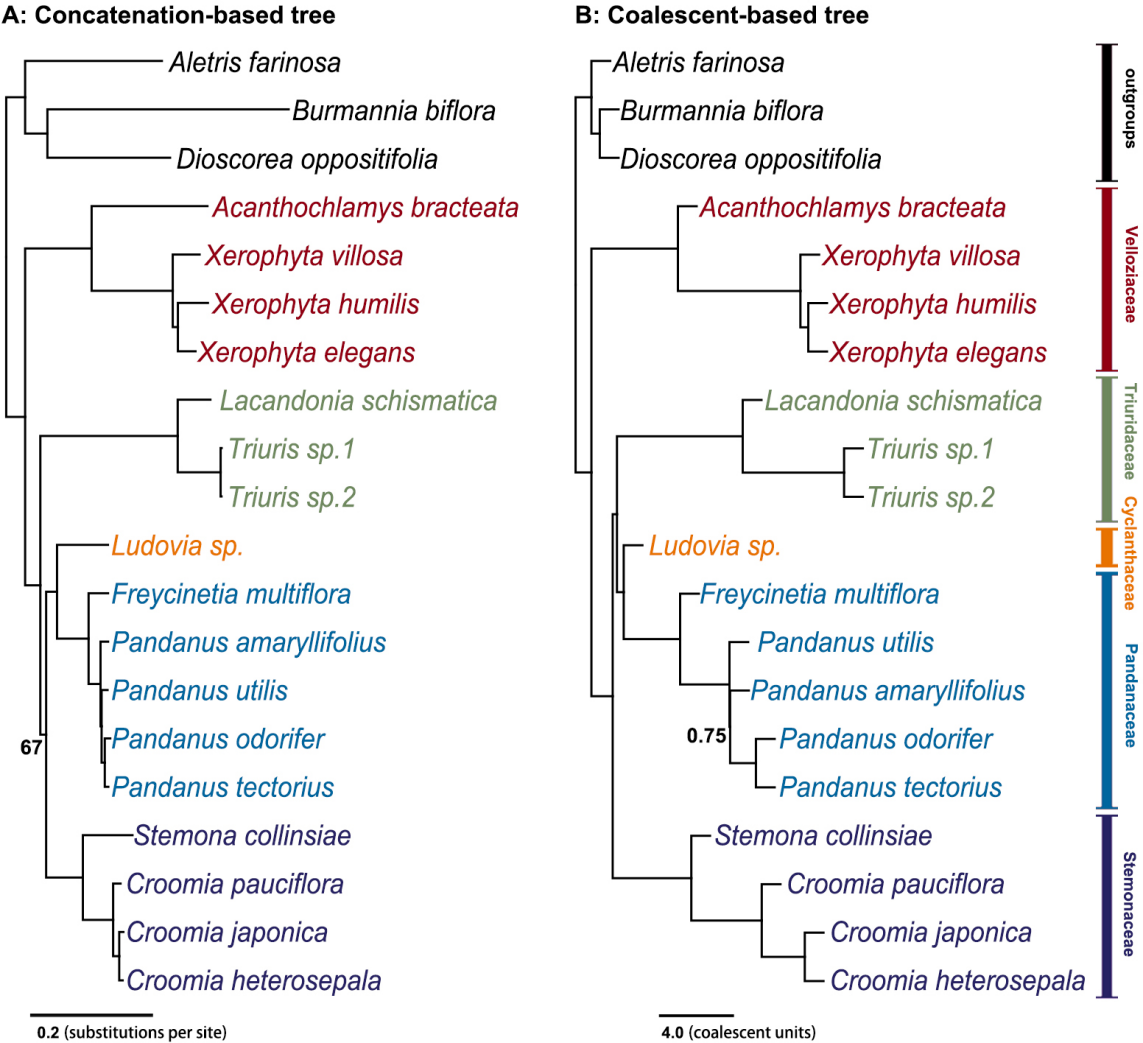
Species tree inferred using both concatenation and coalescent-based methods from 2,668 single-copy orthologous nuclear genes. **(A)** The concatenation-based tree was constructed using the maximum likelihood (ML) method, with numbers next to the nodes indicating bootstrap support values; unlabeled nodes have 100% bootstrap support. **(B)** The coalescent-based tree was inferred using ASTRAL (Zhang et al., 2018), with node labels indicating local posterior probabilities (ASTRAL-lpp); unlabeled nodes have a probability of 1.00.

Both concatenation and coalescent-based analyses of 2668 SCOGs (transcriptome dataset) produced a well-resolved phylogeny of Pandanales, with nearly all clades receiving full support (**Figure 1**). All five families were fully supported. After adding 12 additional samples from the Kew Tree of Life project (see Method 2.3), although the number of SCOGs significantly decreased to 315 (**Supplementary Figure S2**), the resulting phylogenetic trees still identified all five families as fully supported monophyletic groups, and the phylogenetic relationships among them remained identical to the transcriptome dataset (**Supplementary Figure S3**).

In these phylogenetic trees, Velloziaceae consistently emerged as the earliest diverging lineage within Pandanales, with the taxonomically debated genus *Acanthochlamys* placed within this family. The subsequent diverging families were Triuridaceae and Stemonaceae. The remaining two families, Cyclanthaceae and Pandanaceae, formed a very stable clade (C-P clade). However, the phylogenetic position of the C-P clade showed a clear difference between the concatenation and coalescent-based trees: in the concatenation-based tree, the this clade was sister to Stemonaceae, whereas in the coalescent-based tree, it was sister to Triuridaceae (**Figure 1**; **Supplementary Figure S3**). For the plastid genome phylogenetic tree, all five families formed monophyletic clades with high support, and the phylogenetic relationships between these families were identical to the coalescent-based tree (**Supplementary Figure S4**).

**Figure 2:**
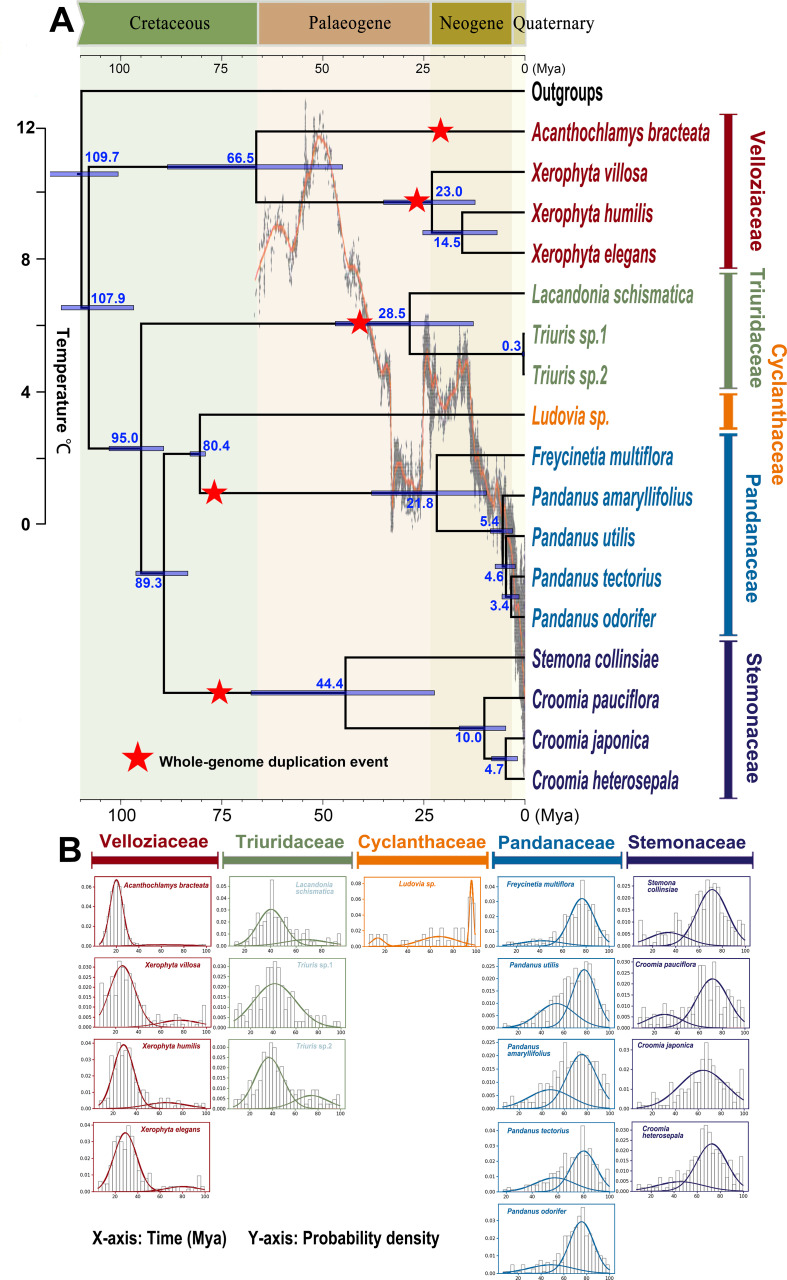
Genome duplication analysis in an absolute dating framework. **(A)** Chronogram displaying divergence times estimated through Bayesian molecular dating using BEAST2. Divergence times are labeled in blue font beneath or above the branches. Detected whole-genome duplication (WGD) events are marked by red stars on the corresponding branches, with the position of each star indicating the approximate timing of the event. **(B)** Absolute age distributions of gene duplications for each Pandanales sample. In each graph, prominent peaks in the curves correspond to WGD events, with the horizontal axis indicating the estimated timing of these events.

Divergence time estimates suggest that the stem age of Pandanales is approximately 109.7 million years ago (Mya) (95% HPD: 100.6–118.6 Mya). The divergence between Velloziaceae and the other families, marking the crown age of Pandanales, occurred around 107.9 Mya (95% HPD: 96.8–114.7 Mya). The origins (stem ages) of the other four families also date back to the Cretaceous period, reinforcing the ancient diversification of Pandanales during this era (**Figure 2A**).

### 3.4 WGD detection

The WGD analysis (**Figure 2B**) revealed several significant WGD events. First, it identified a distinct peak of paralogous gene duplications around 80 million years ago (Mya) for both the Stemonaceae and Pandanaceae clades, with WGD occurring after the divergence of these two families (**Figure 2B**), indicating two separate ancient WGD events. A similar peak was observed around 40 Mya for the stem of Triuridaceae, suggesting a WGD event in that lineage. Additionally, two independent peaks were detected in Velloziaceae: one around 26 Mya in the stem lineage of *Xerophyta*, and another around 24 Mya in the stem lineage of *Acanthochlamys* (**Figure 2A**). The latter was further confirmed by a whole-genome dot plot approach, which revealed a clear WGD event (**Supplementary Figure S5**).

**Figure 3:**
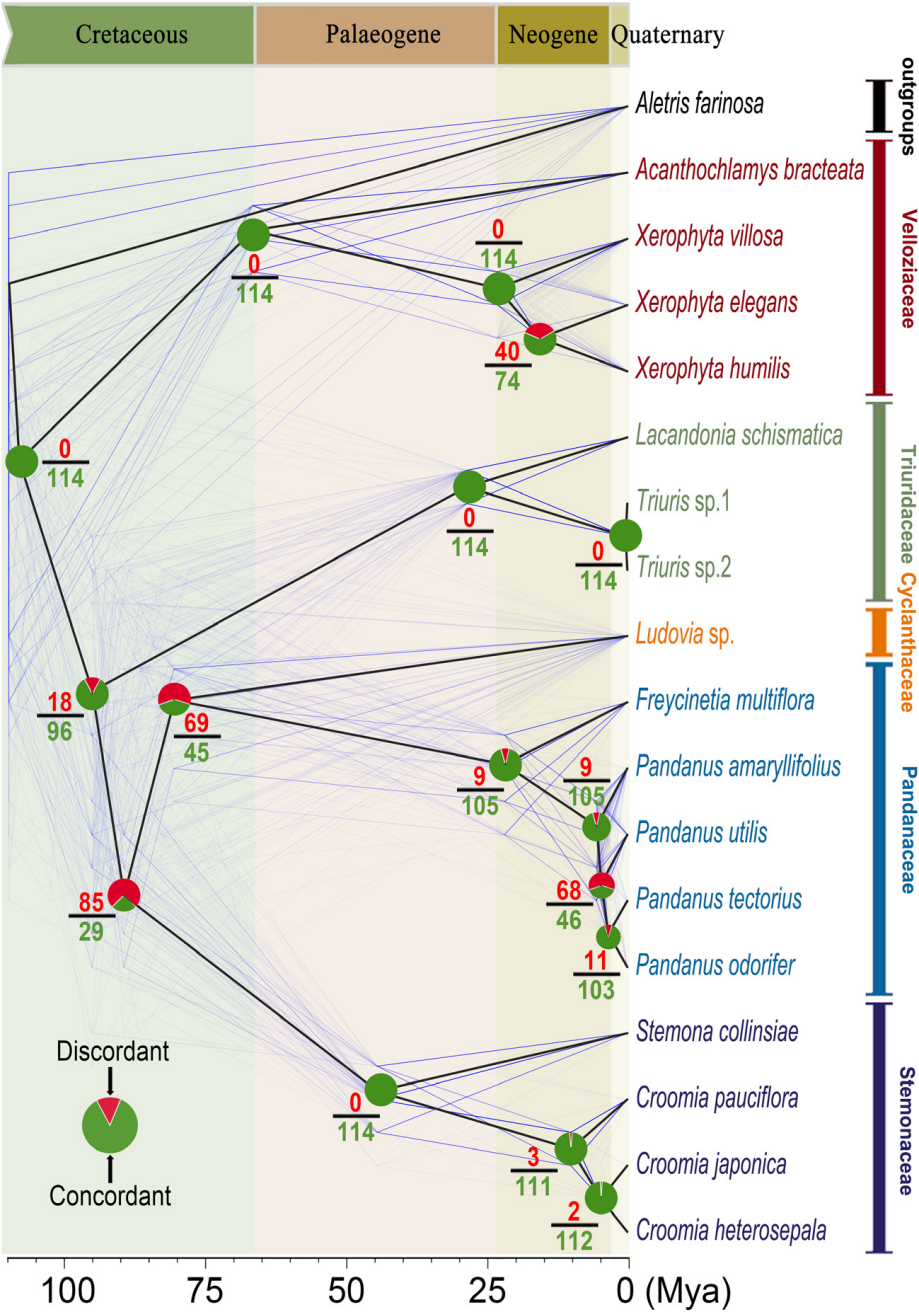
Gene tree-species tree discordance. The chronogram of Pandanales, generated using Bayesian molecular dating in BEAST2, is depicted with thick black lines. Gray trees (“cloud trees”) represent 114 single-copy orthologous nuclear genes (with no missing taxa and >600 parsimony-informative sites) and were constructed using the maximum likelihood (ML) method, with divergence times estimated via treePL. Pie charts and the surrounding numbers at the nodes illustrate the proportions of concordant and discordant gene tree topologies relative to the species tree.

### 3.5 Phylogenetic conflict analysis

At most nodes, the majority of gene trees exhibited a high degree of concordance with the species tree (**Figure 3**). For example, the families Velloziaceae, Triuridaceae, and Stemonaceae were each supported by 100% (114/114) of the nuclear gene trees. Similarly, Pandanaceae was supported by 92.1% (105/114) of the informative gene trees.

Despite the strong concordance observed within most families, the relationships between families, particularly at the crown nodes of the Stemonaceae-Cyclanthaceae-Pandanaceae clade (25.4% concordance; 29/114 gene trees) and the Cyclanthaceae-Pandanaceae clade (39.5% concordance; 45/114 gene trees), exhibited substantial gene tree discordance (**Figure 3**). This indicates that resolving the deeper evolutionary relationships between these families remains challenging.

**Figure 4:**
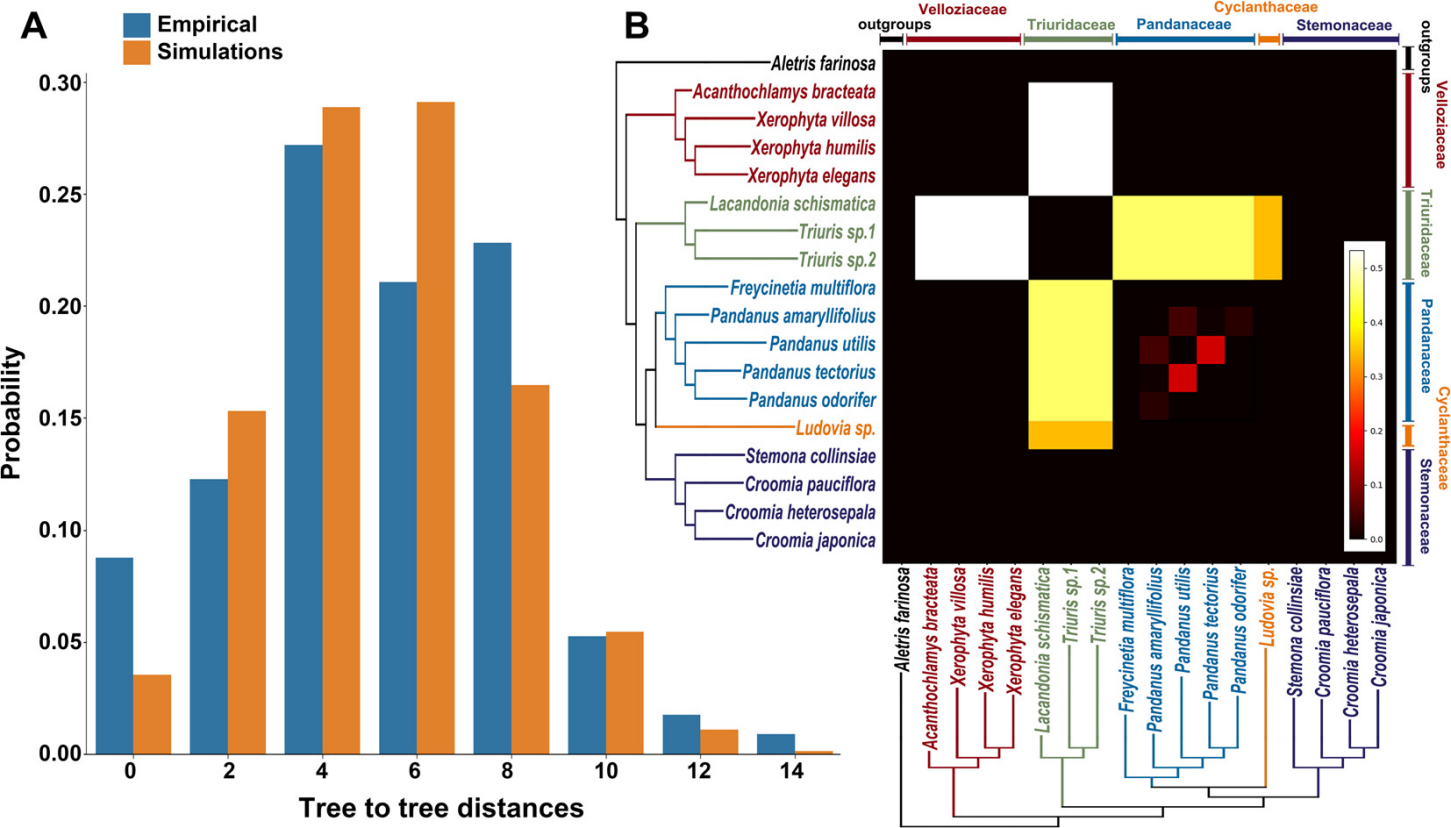
Evaluating the roles of incomplete lineage sorting (ILS) and gene flow in gene tree–species tree conflicts. **(A)** Comparison of tree-to-tree distance distributions between the species tree (**Figure 1A**) and the empirical gene trees (blue bars), and between the species tree and gene trees simulated under a coalescent model (orange bars). The differences in these distributions suggest that processes other than ILS, such as gene flow, may contribute to the observed conflicts. **(B)** QuiBL analysis. The heatmap shows the degree of topological conflict between pairs of samples (x- and y-axes) across all gene trees. Brighter squares indicate stronger evidence that gene flow, rather than ILS, is the predominant factor driving the conflicts between these pairs of taxa.

By comparing the distance distributions (**Figure 4A**) derived from empirical observations with those generated through simulations, the overall shapes of the two distributions were largely similar. The most frequent tree-to-tree distances for empirical data were 4 and 8, whereas for simulations they were 4 and 6. This suggests that incomplete lineage sorting (ILS) is the primary cause of gene tree conflicts during the evolutionary history of Pandanales. However, the results of the chi-square test yielded a p-value of 0.0065, leading to the rejection of the null hypothesis and indicating that ILS alone cannot sufficiently explain the observed discrepancies among gene trees. This suggests that gene flow also plays a role in these discrepancies.

Furthermore, the analysis using QuIBL reinforced this finding (**Figure 4B**). The results indicated that gene flow contributed more than 50% to the gene tree conflicts between Velloziaceae and Triuridaceae, and gene flow also contributed nearly 50% to the gene tree conflicts between Triuridaceae and Pandanaceae. In contrast, conflicts among other branches are likely primarily due to ILS.

### 3.6 Assessment of gene flow

**Figure 5:**
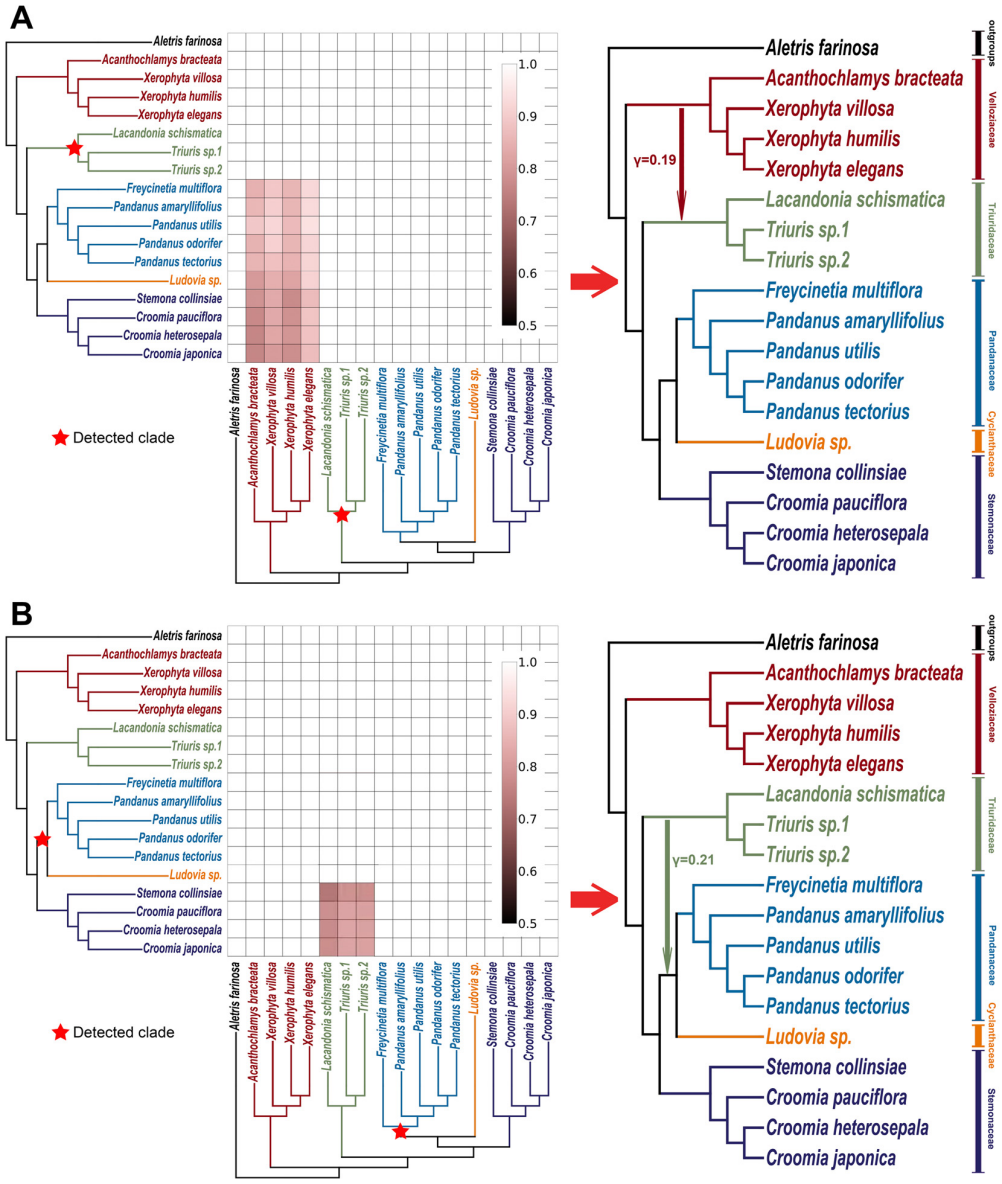
Gene flow detection in pandanales using HyDe. **(A)** Triuridaceae-associated gene flow detection. The detected clade is marked with a red star on the cladogram. In the heatmap, small red squares indicate significant gene flow signals identified by HyDe, where the taxa on the left axis represent the primary genetic contributors, and those on the bottom axis are the secondary contributors (the groups that have experienced gene flow with the detected clade). The color intensity indicates the inheritance probability for the corresponding taxa on the left axis. When squares of similar color intensity cluster into a larger block and the associated taxa form a monophyletic clade, it suggests that the most recent common ancestor of that clade may be one of the parental species (for detailed methods, see https://github.com/Jhe1004/VisualHyde). On the right, a schematic diagram based on the cladogram and heatmap illustrates the inferred gene flow relationships, with numerical values indicating inheritance probabilities. **(B)** Pandanaceae-associated gene flow detection, following the same procedure as in **(A)**.

**Figure 6:**
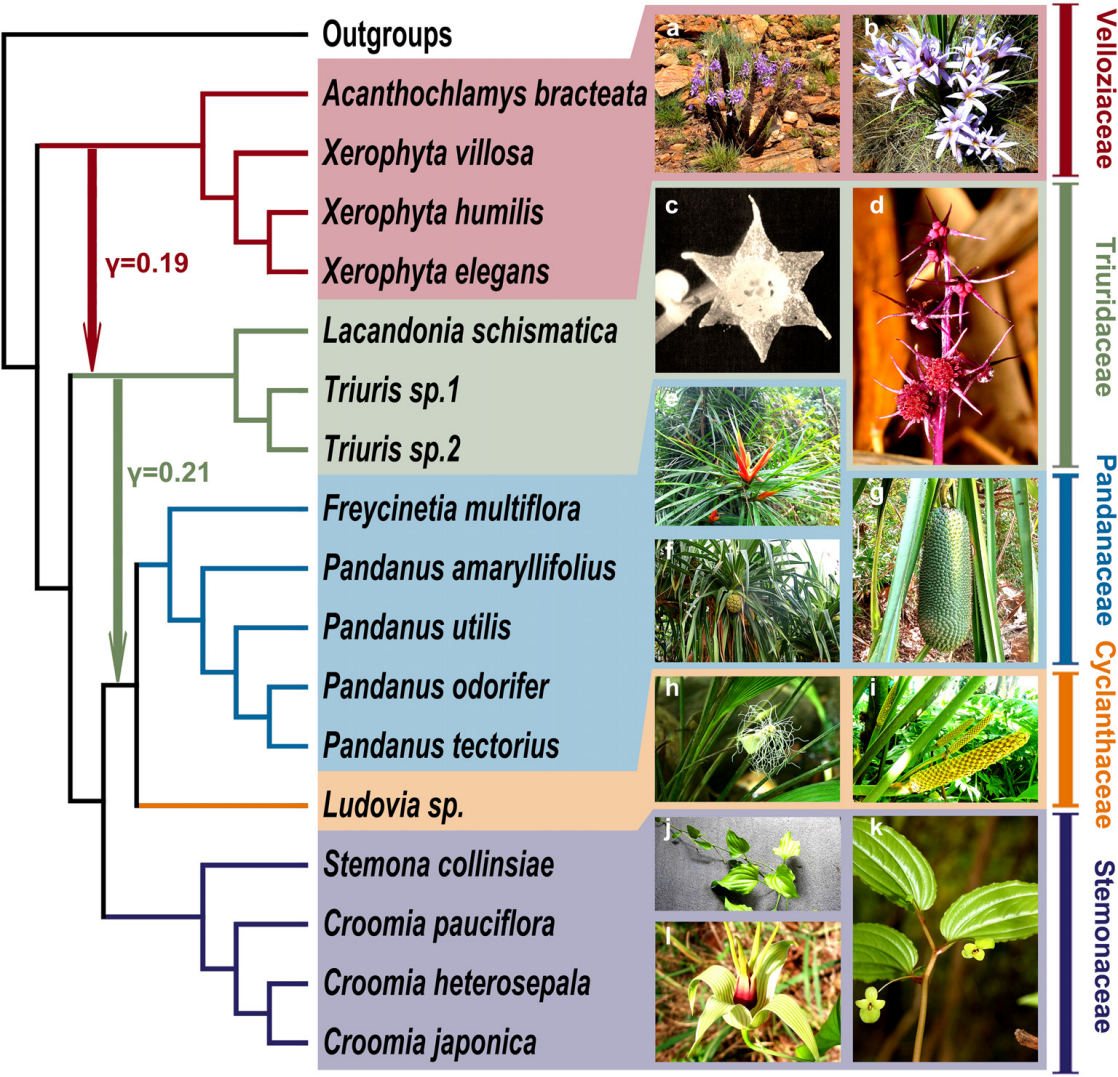
Gene flow network in pandanales inferred from HyDe results. (Left) a schematic diagram based on the cladogram and heatmap illustrates the inferred gene flow relationships, with numerical values indicating inheritance probabilities. (Right) Field photographs of representative species from each family. **(A, B)** *Xerophyta retinervis*; **(C)** *Lacandonia schiamatica*; **(D)** *Sciaphila secundiflora*; **(E)** *Freycinetia excelsa*; **(F)** *Pandanus dubius*; **(G)** *Pandanus unipapillatus*; **(H)** *Dicranopygium yacu*; **(I)** *Carludovica palmata*; **(J)** *Stemona tuberosa*; **(K)** *Croomia heterosepala*; **(L)** *Stemona tuberosa*.

Using HyDe software (Blischak et al., 2018) and its associated visualization script (https://github.com/Jhe1004/VisualHyde), several significant gene flow events between different families were detected within Pandanales (**Figures 5** and **6**; **Supplementary Figures S6** and **S7**). Notably, a substantial gene flow event was identified between Triuridaceae and Velloziaceae, with approximately 19% of the genetic material in Triuridaceae genomes originating from Velloziaceae (**Figures 5A** and **6**). Additionally, gene flow was detected between Triuridaceae and C-P clade (Cyclanthaceae + Pandanaceae), with about 13% of the genetic material in C-P clade genomes deriving from Triuridaceae (**Figures 5B** and **6**).

To further validate these gene flow events, PhyloNet software (Wen et al., 2018) was employed. When allowing for up to three gene flow events, the analysis first produced a local maximum likelihood score (**Supplementary Figure S8F**). Under these conditions, the software was run five independent iterations, yielding consistent results across all runs and nearly identical tree topologies (**Supplementary Figure S8A–E**). The gene flow events between Triuridaceae and Velloziaceae, as well as between Triuridaceae and C-P clade, detected by HyDe, were similarly identified by PhyloNet.

## 4 Discussion

### 4.1 Resolving phylogenetic conflicts in Pandanales: insights from gene flow analysis

In this study, by analyzing 2,668 SCOGs, we reconstructed an almost fully supported phylogeny of Pandanales using both concatenation- and coalescent-based methods (**Figure 1**; **Supplementary Figure S3**). Additionally, we generated a phylogenetic tree from whole-plastid genome data (**Supplementary Figure S4**). However, these three phylogenies exhibited conflicting topologies (**Figure 1**; **Supplementary Figures S3**, **S4**). Consistent with previous findings, the primary point of conflict centered on the placement of the clade comprising Cyclanthaceae and Pandanaceae (C-P clade). In the concatenation-based tree, the C-P clade was resolved as sister to Stemonaceae, matching the results of Mennes et al. (2013) and Baker et al. (2022). By contrast, both the coalescent-based and plastid genome trees supported a sister relationship between the C-P clade and Triuridaceae, in line with Lam et al. (2016, 2018), Givnish et al. (2018), and Soto Gomez et al. (2020).

Debates over concatenation- versus coalescent-based phylogenies are common in systematics, particularly for groups with complex evolutionary histories (Song et al., 2012; Gatesy and Springer, 2014; Springer and Gatesy, 2016; Edwards et al., 2016). Discrepancies often stem from incomplete lineage sorting (ILS), differences between nuclear and organellar signals, and biases related to long branches or limited taxon sampling (Song et al., 2012; Gatesy and Springer, 2014). Although coalescent approaches explicitly address ILS, they generally assume no within-locus recombination—an assumption frequently violated in longer protein-coding sequences, where multiple recombination breakpoints can distort gene tree topologies and reduce the accuracy of species tree inferences (Song et al., 2012; Gatesy and Springer, 2014; Springer and Gatesy, 2016).

Our study provides a novel perspective on this debate. Using HyDe for gene flow analysis, we found that Stemonaceae contributed approximately 79% of the genetic material to the C-P clade, whereas Triuridaceae contributed only 21% (**Figures 5**, **6**). This finding indicates that the C-P clade is more closely aligned with Stemonaceae, thereby supporting the concatenation-based topology. Furthermore, the discrepancy with the plastid genome tree may be explained by the capture of Triuridaceae plastids by the C-P clade during historical gene flow events. Consequently, we propose that the concatenation-based topology may more accurately reflect the true species tree for Pandanales.

In addition, we observed significant discordance between gene trees and the species tree, revealing a distinct pattern: while gene trees and the species tree were largely congruent at backbone nodes within individual families, notable conflicts emerged at the divergence points between families (**Figure 3**). This pattern may clarify the long-standing debate concerning the phylogenetic placement of Pandanales families, as different genetic markers have often produced conflicting phylogenetic outcomes. Further analyses of the causes of these gene-tree–species-tree conflicts showed that, for several strongly discordant nodes—such as the stem node of the C-P clade (**Figure 3**)—gene flow, rather than ILS, was the primary factor (**Figure 4**).

In summary, we ultimately credible a highly supported species tree for Pandanales (**Figure 1A**). Our findings suggest that the extensive heterogeneity among gene trees, which has long complicated the phylogenetic relationships among the five families, is more likely attributable to gene flow than to ILS.

### 4.2 The role of whole-genome duplication and gene flow in shaping Pandanales evolution

Our findings reveal five distinct WGD events in Pandanales, each coinciding with significant geological or climatic transitions (**Figure 2**). Notably, two of these events occurred on the stem of Stemonaceae and Pandanaceae just before the Cretaceous–Paleogene (K–Pg) boundary, a period (~66 Ma) marked by the Cretaceous mass extinction. Although these same two WGDs were detected by One KP (2019), that study did not estimate their timing. Our results now place them in a critical window of global upheaval, implying that polyploidy may have conferred adaptive advantages or facilitated lineage survival during this mass extinction event (Fawcett et al., 2009; Vanneste et al., 2014).

Of the remaining three WGD events, one occurred during the mid-Paleogene on the stem lineage of Triuridaceae, whereas two occurred near the boundary between the Paleogene and Neogene in Velloziaceae (**Figure 2**). Specifically, these latter WGDs were detected on the stem of *Acanthochlamys* and *Xerophyta*, with the latter also reported in One KP (2019). These periods are similarly characterized by considerable climatic fluctuation (Zachos et al., 2001), underscoring the hypothesis that WGDs can arise when environmental pressures are high. In such scenarios, the genomic redundancy provided by WGD may enable rapid adaptation, either by buffering deleterious mutations or by allowing neofunctionalization of gene duplicates (Wang et al., 2011; Wu et al., 2020).

Together, these five WGDs contribute to a growing body of evidence that links polyploidy with macroevolutionary success in plants (Moriyama and Koshiba-Takeuchi, 2018; Carretero-Paulet and Van de Peer, 2020; Hämälä et al., 2024). Although the precise effect of WGD on diversification rates remains an area of active debate (Fawcett et al., 2009; Cai et al., 2019; Koenen et al., 2021; Soltis and Soltis, 2021), there is a broad consensus that genome duplications can promote genetic innovation, lineage persistence, and ecological expansion. In the case of Pandanales, the temporal alignment of these WGDs with known periods of climatic or geological change—ranging from the K–Pg boundary to later Paleogene–Neogene transitions—suggests that polyploidy may have played a pivotal role in the evolution and diversification of its constituent families.

In addition to the five detected WGD events, we identified two notable gene flow events in Pandanales. Although their exact timing remains uncertain, these events, occurring between Velloziaceae and Triuridaceae and between Triuridaceae and the C-P clade, likely predate the crown diversification of Velloziaceae, suggesting an origin at least before 66.5 Ma. Increasing evidence indicates that gene flow can facilitate rapid adaptation by introducing novel alleles into recipient lineages (Comeault and Matute, 2018; Marques et al., 2019; McGee et al., 2020). In this study, these gene flow events may have enabled lineages to cope with or even thrive amidst the rapidly changing environments of the Late Cretaceous. These findings underscore the dual role of WGD and gene flow in shaping the evolutionary trajectories of Pandanales.

## Data Availability

The data that support the findings of this study are openly available in the Science Data Bank at https://doi.org/10.5281/zenodo.13857688.

## Supplementary material

Supplementary Figure 1 Heatmap depicting the recovery efficiency of single-copy orthologous nuclear genes (SCOGs) recovered through transcriptome sequencing. The heatmap shows the recovery efficiency of 2,668 SCOGs across individual samples (rows). Columns represent each SCOG analyzed. Blue squares indicate the presence of a SCOG, while white squares denote its absence, highlighting sample-specific differences in gene recovery.

Supplementary Figure 2 Heatmap depicting the recovery efficiency of single-copy orthologous nuclear genes (SCOGs) recovered by combining transcriptome data with the Universal Angiosperms353 Probe Set data. The heatmap illustrates the recovery efficiency of 315 SCOGs across individual samples (rows). Columns represent each SCOG analyzed. Blue squares indicate the successful recovery of a SCOG, while white squares denote its absence, showcasing the performance of the combined approach.

Supplementary Figure 3 Species tree inferred using both concatenation and coalescent-based methods from 315 single-copy orthologous nuclear genes (recovered by combining transcriptome data with the Universal Angiosperms353 Probe Set data). **(A)** The concatenation-based tree was constructed using the maximum likelihood (ML) method, with numbers next to the nodes indicating bootstrap support values; unlabeled nodes have 100% bootstrap support. **(B)** The coalescent-based tree was inferred using ASTRAL (Zhang et al., 2018), with node labels indicating local posterior probabilities (ASTRAL-lpp); unlabeled nodes have a probability of 1.00.

Supplementary Figure 4 Maximum likelihood phylogenetic tree of Pandanales based on complete plastid genomes. The phylogenetic tree was constructed using the maximum likelihood (ML) method, with numbers next to the nodes representing bootstrap support values. Nodes without labels indicate 100% bootstrap support.

Supplementary Figure 5 Whole-genome duplication dot plots of *Acanthochlamys bracteata*. The duplication signals are highlighted with blue circles.

Supplementary Figure 6 Gene flow detection based on the results of HyDe, with heatmaps drawn for each sample in Pandanales (see Methods 2.7).

Supplementary Figure 7 Gene flow detection based on the results of HyDe, with heatmaps drawn for each internal node in the phylogeny of Pandanales(see Methods 2.7).

Supplementary Figure 8 Phylogenetic network of Pandanales constructed using single-copy orthologous genes with PhyloNet. **(A-E)** Phylogenetic networks generated from five independent runs where the maximum number of allowed gene flow events was set to 3. The numbers next to the curves represent inheritance values. **(F)** Likelihood scores for the phylogenetic networks obtained when the maximum number of gene flow events ranged from 0 to 10.

Supplementary Table 1 Sample information for RNA raw sequencing quality, *de novo* assembly statistics, NCBI-SRA accession numbers, and chloroplast genome data, including genome size, number of protein-coding genes, and accession numbers used in this study.

Supplementary Table 2 Alignment details for 2221 single-copy orthologous genes.

Supplementary Table 3 Sample information for 12 additional samples from the Kew Tree of Life project (https://treeoflife.kew.org/tree-of-life/search-name/?order=Pandanales).

Supplementary Table 4 Raw Output File from HyDe Software.
