# Supplementary figures and images for: Resolving phylogenetic conflicts in Pandanales: the dual roles of gene flow and whole-genome duplication
Source: Front Plant Sci. 2025 Feb 24;16:1511582. doi: 10.3389/fpls.2025.1511582 (PMC11891173; doi:10.3389/fpls.2025.1511582)

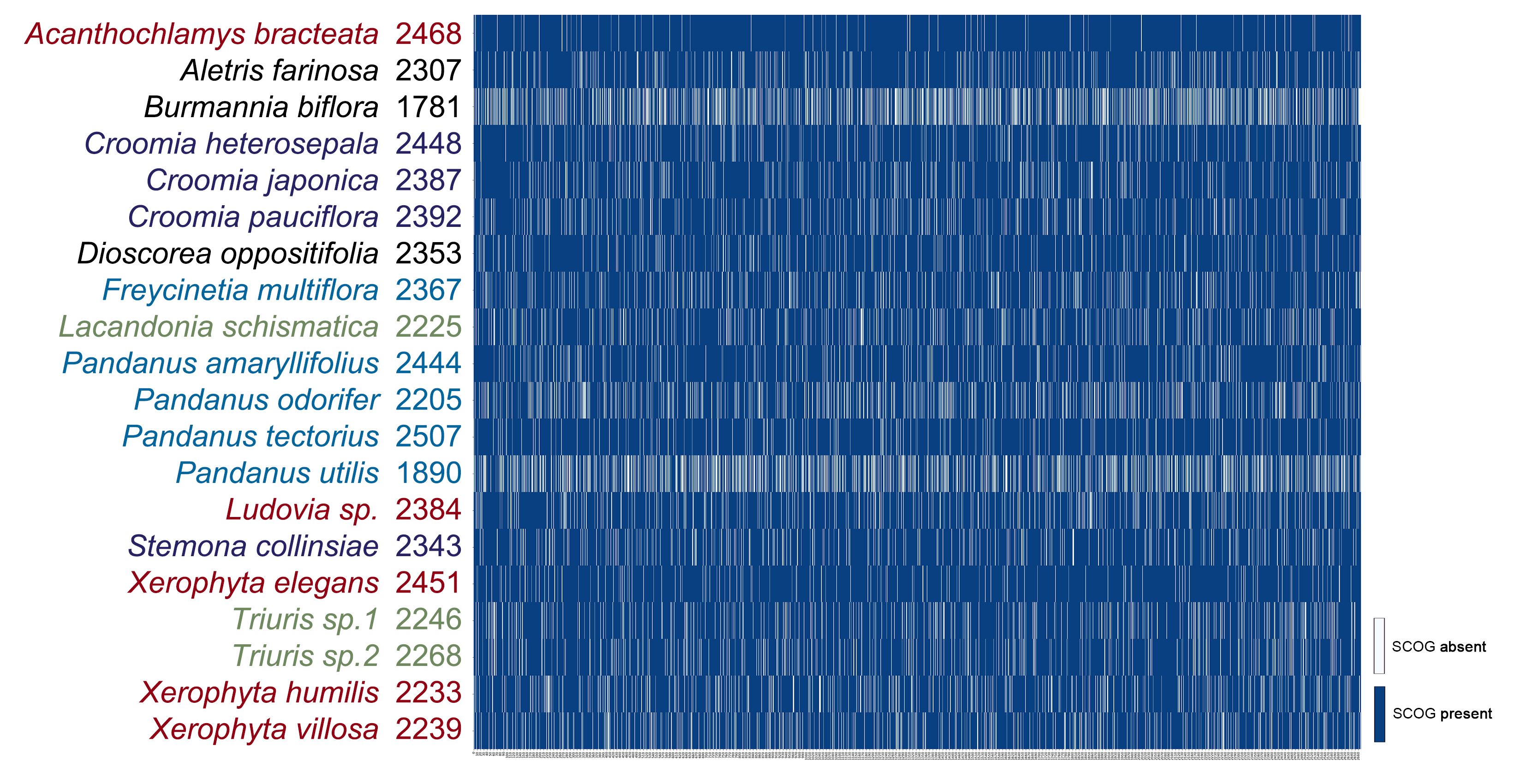

Supplement: Supplementary Figure 1 — Heatmap depicting the recovery efficiency of single-copy orthologous nuclear genes (SCOGs) recovered through transcriptome sequencing. The heatmap shows the recovery efficiency of 2,668 SCOGs across individual samples (rows). Columns represent each SCOG analyzed. Blue squares indicate the presence of a SCOG, while white squares denote its absence, highlighting sample-specific differences in gene recovery. [file Image1.jpeg]

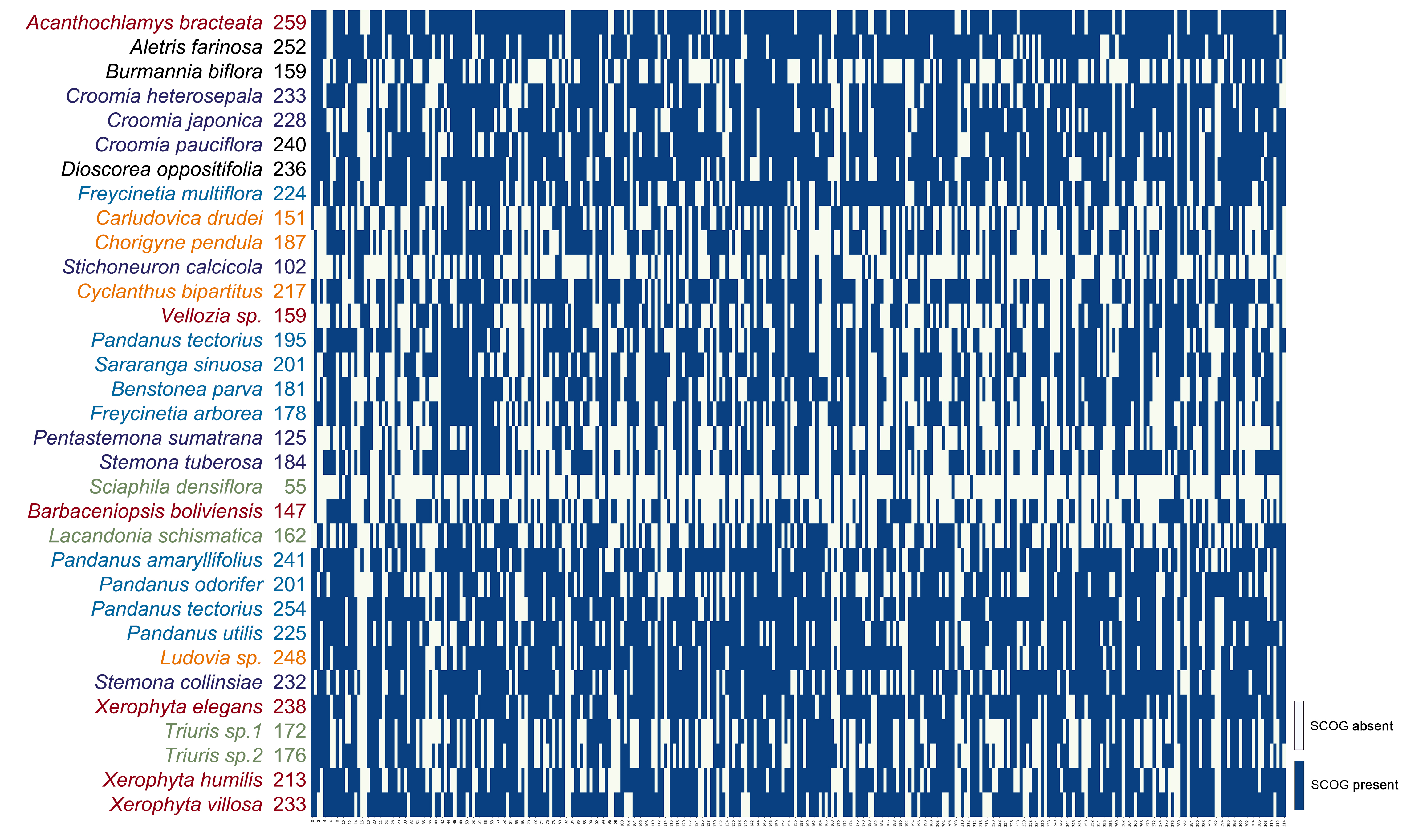

Supplement: Supplementary Figure 2 — Heatmap depicting the recovery efficiency of single-copy orthologous nuclear genes (SCOGs) recovered by combining transcriptome data with the Universal Angiosperms353 Probe Set data. The heatmap illustrates the recovery efficiency of 315 SCOGs across individual samples (rows). Columns represent each SCOG analyzed. Blue squares indicate the successful recovery of a SCOG, while white squares denote its absence, showcasing the performance of the combined approach. [file Image2.jpeg]

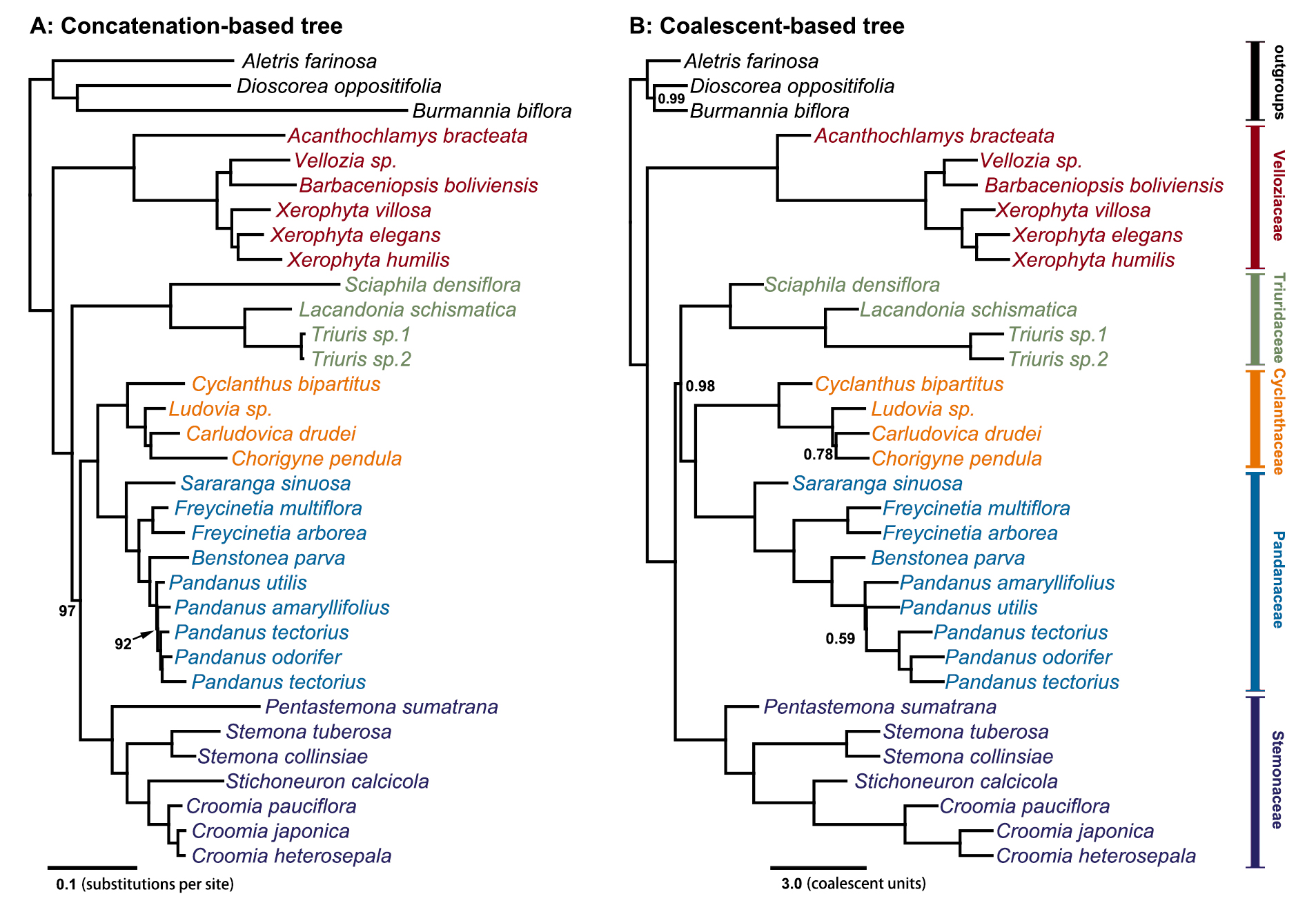

Supplement: Supplementary Figure 3 — Species tree inferred using both concatenation and coalescent-based methods from 315 single-copy orthologous nuclear genes (recovered by combining transcriptome data with the Universal Angiosperms353 Probe Set data). (A) The concatenation-based tree was constructed using the maximum likelihood (ML) method, with numbers next to the nodes indicating bootstrap support values; unlabeled nodes have 100% bootstrap support. (B) The coalescent-based tree was inferred using ASTRAL (Zhang et al., 2018), with node labels indicating local posterior probabilities (ASTRAL-lpp); unlabeled nodes have a probability of 1.00. [file Image3.jpeg]

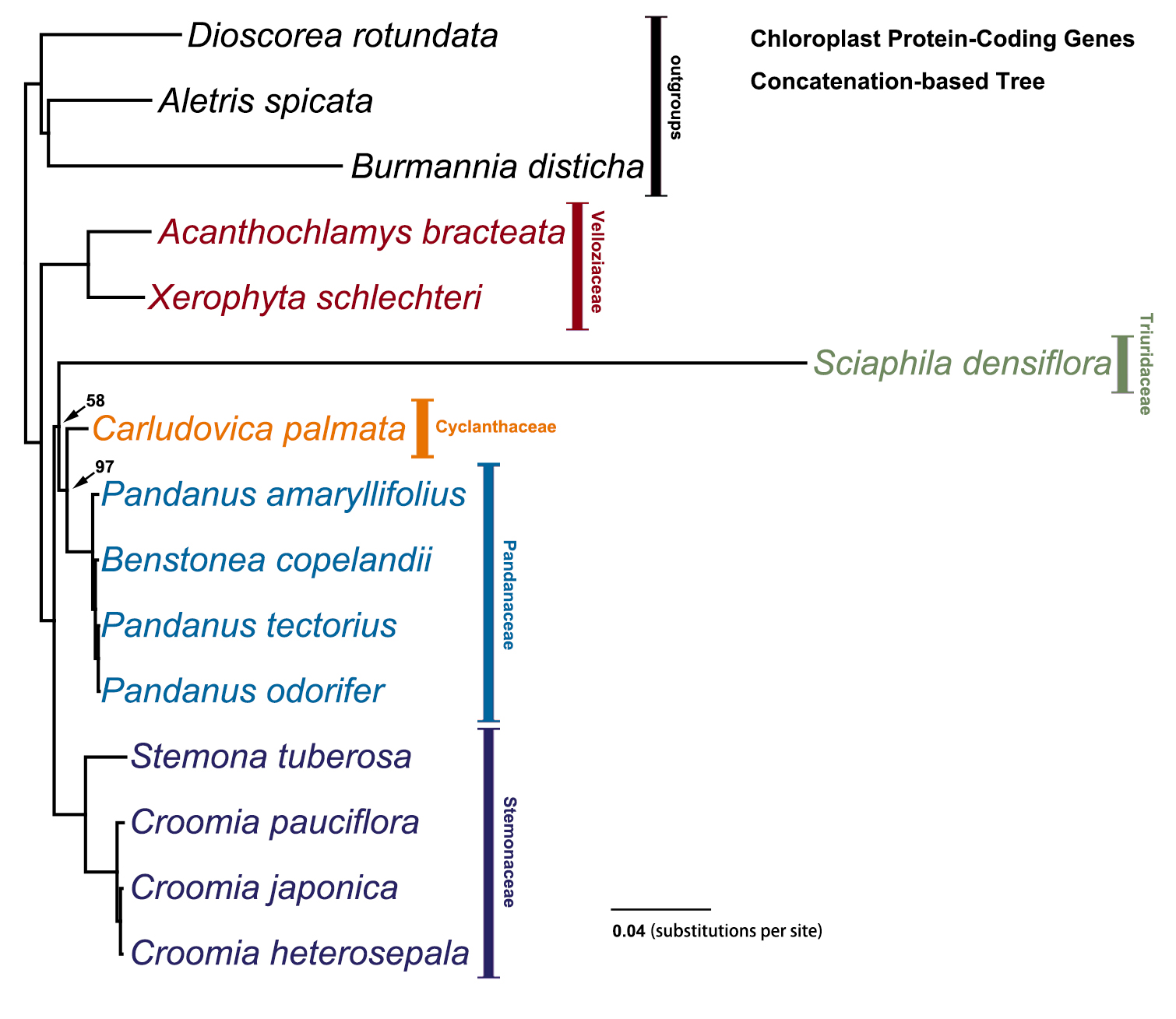

Supplement: Supplementary Figure 4 — Maximum likelihood phylogenetic tree of Pandanales based on complete plastid genomes. The phylogenetic tree was constructed using the maximum likelihood (ML) method, with numbers next to the nodes representing bootstrap support values. Nodes without labels indicate 100% bootstrap support. [file Image4.jpeg]

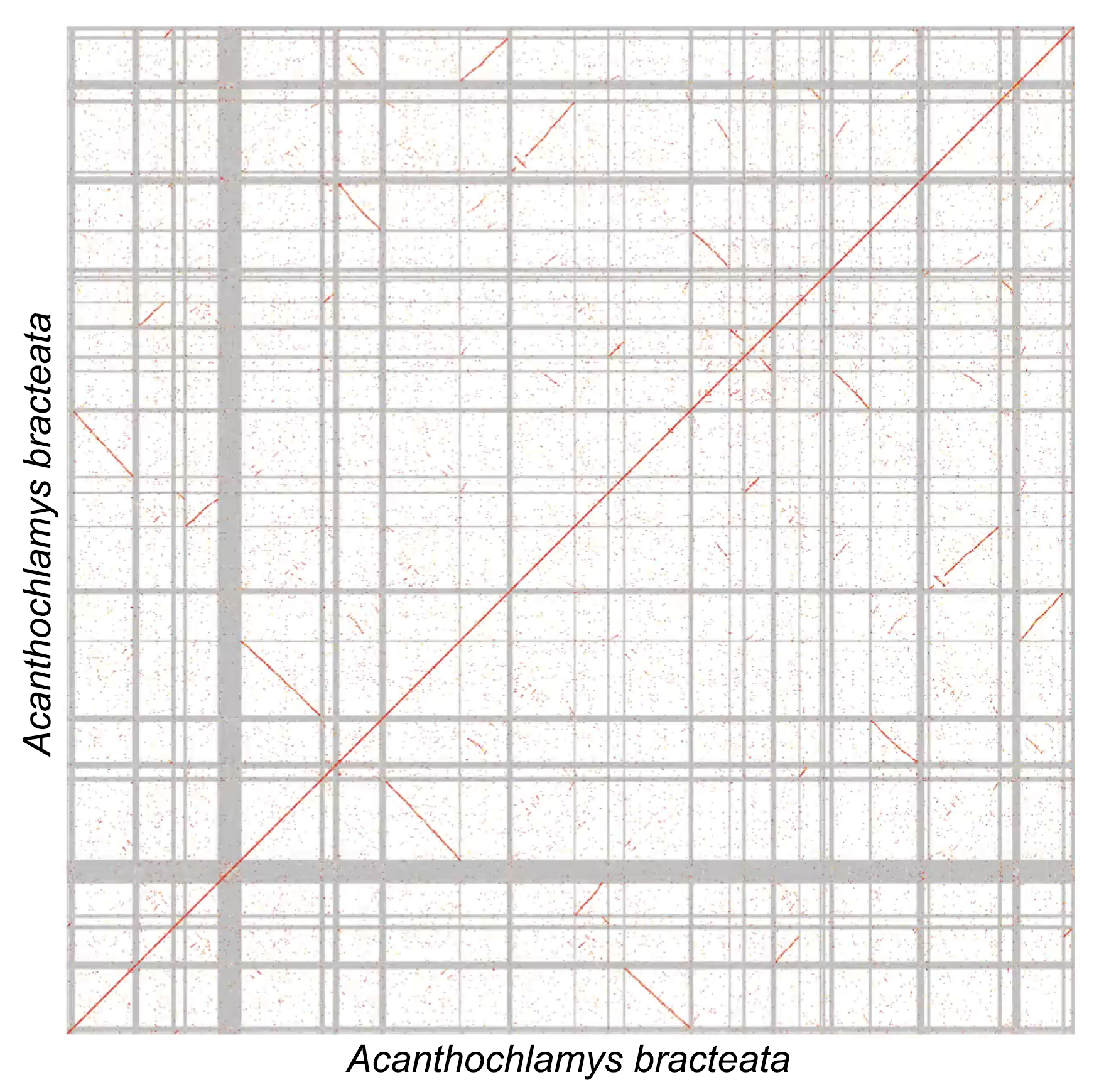

Supplement: Supplementary Figure 5 — Whole-genome duplication dot plots of Acanthochlamys bracteata. The duplication signals are highlighted with blue circles. [file Image5.jpeg]

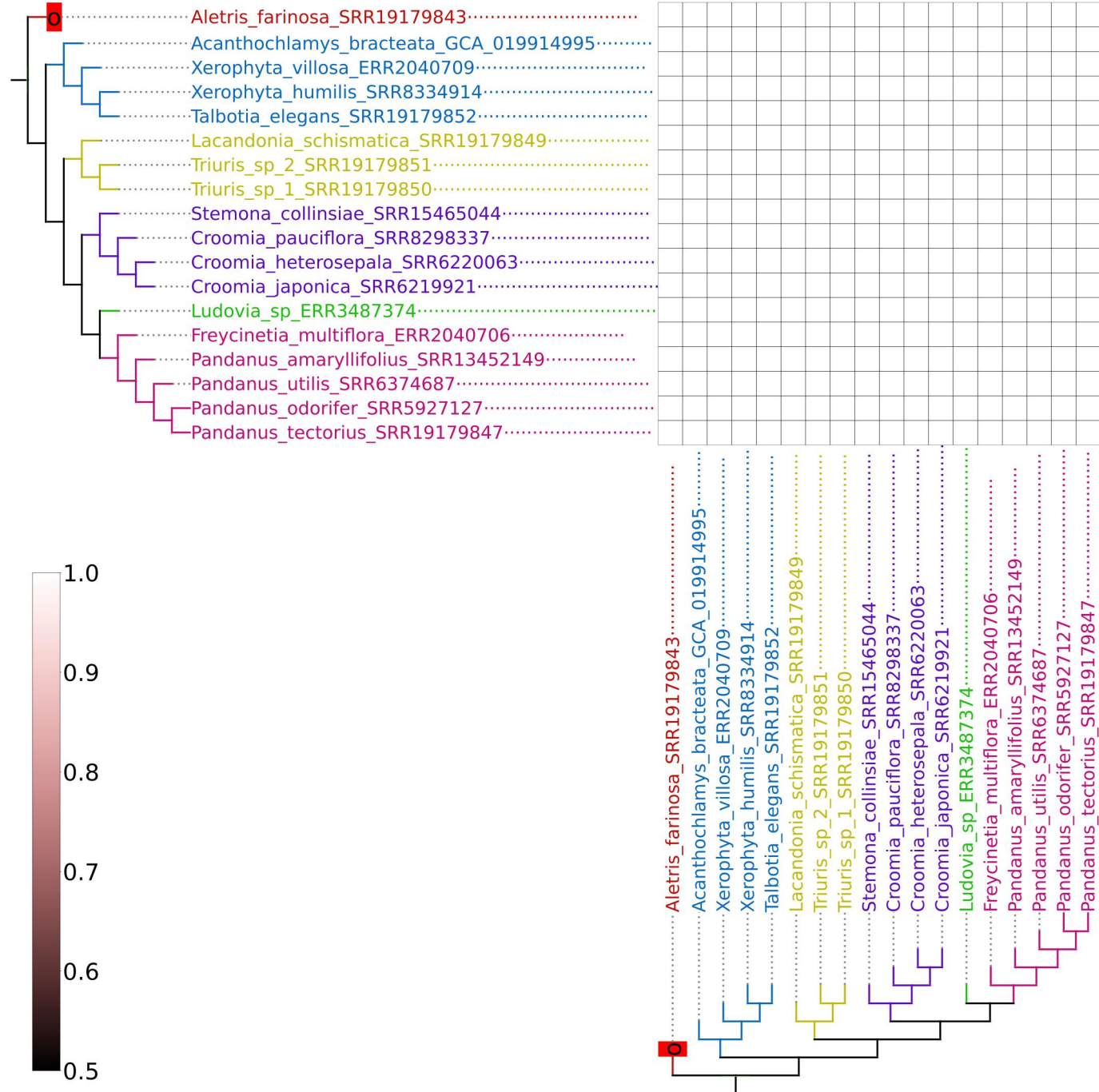

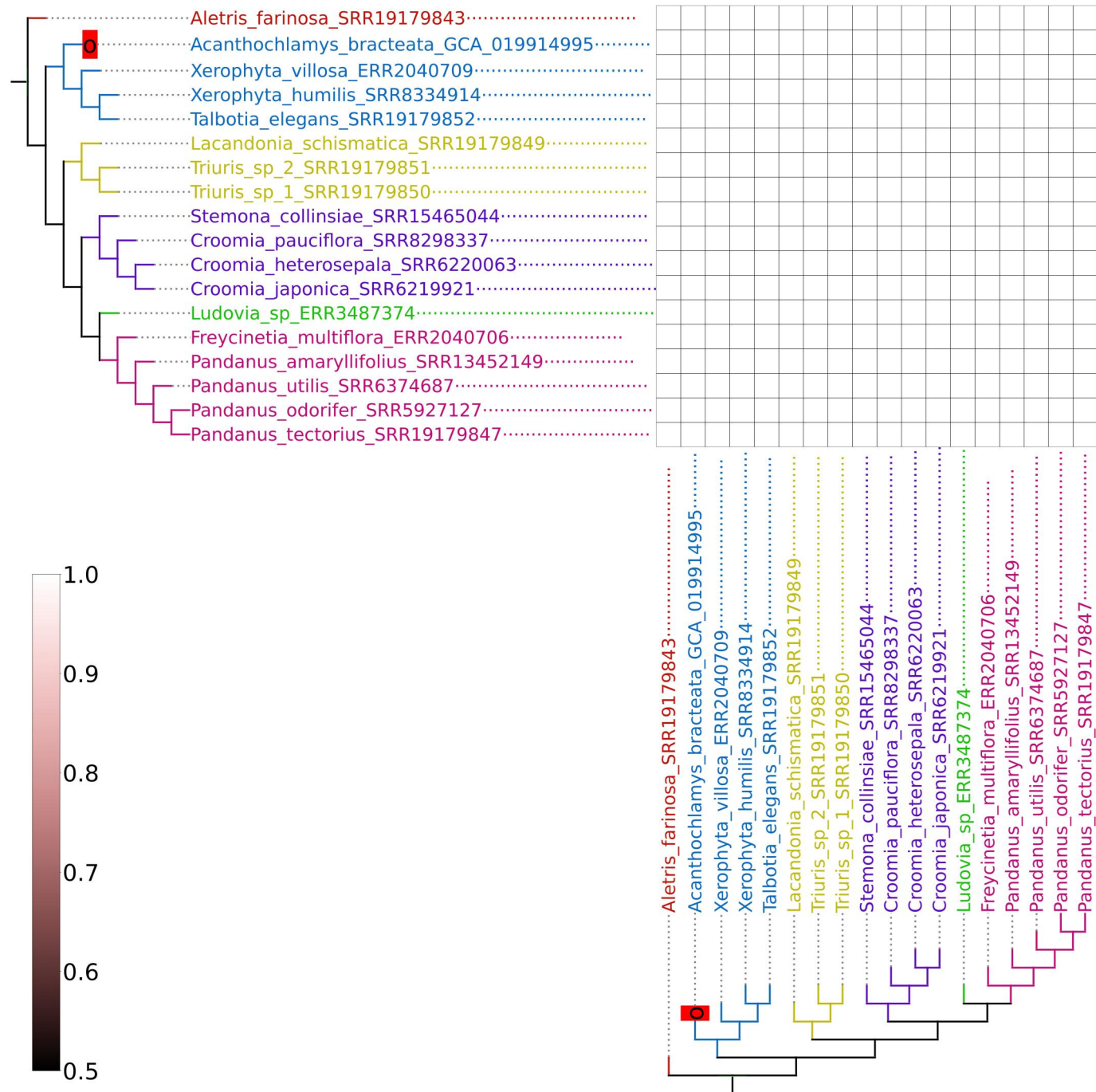

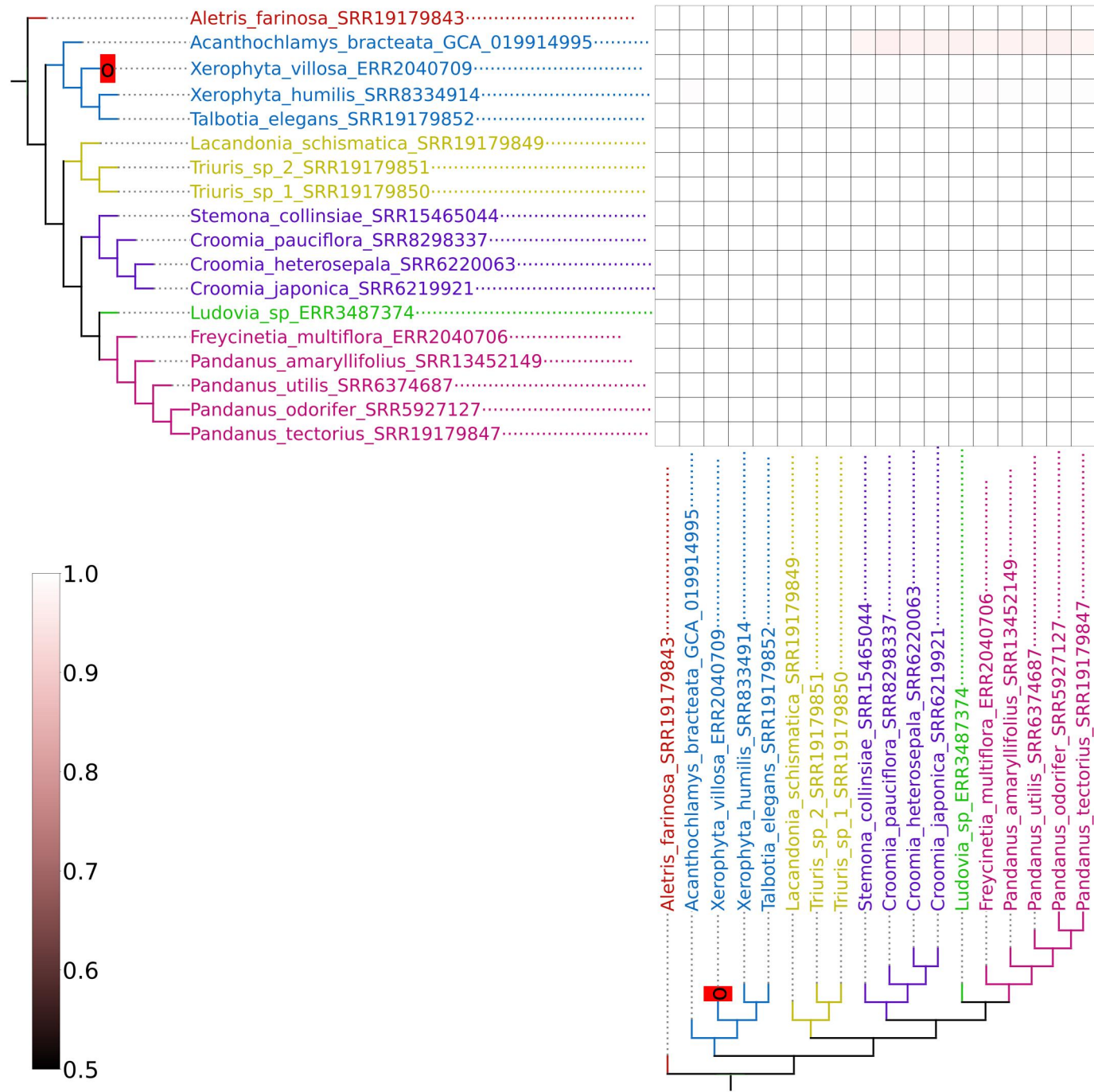

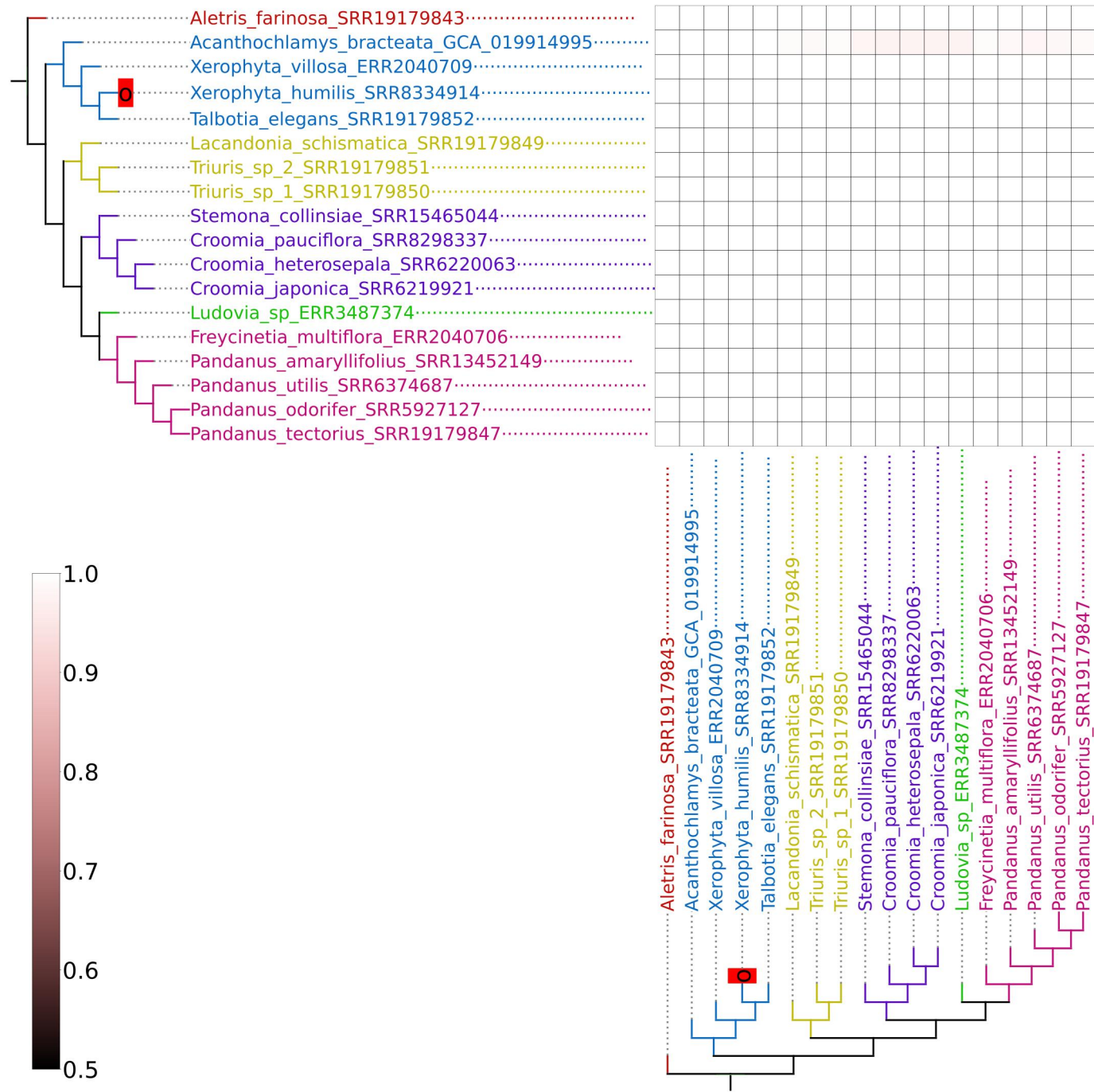

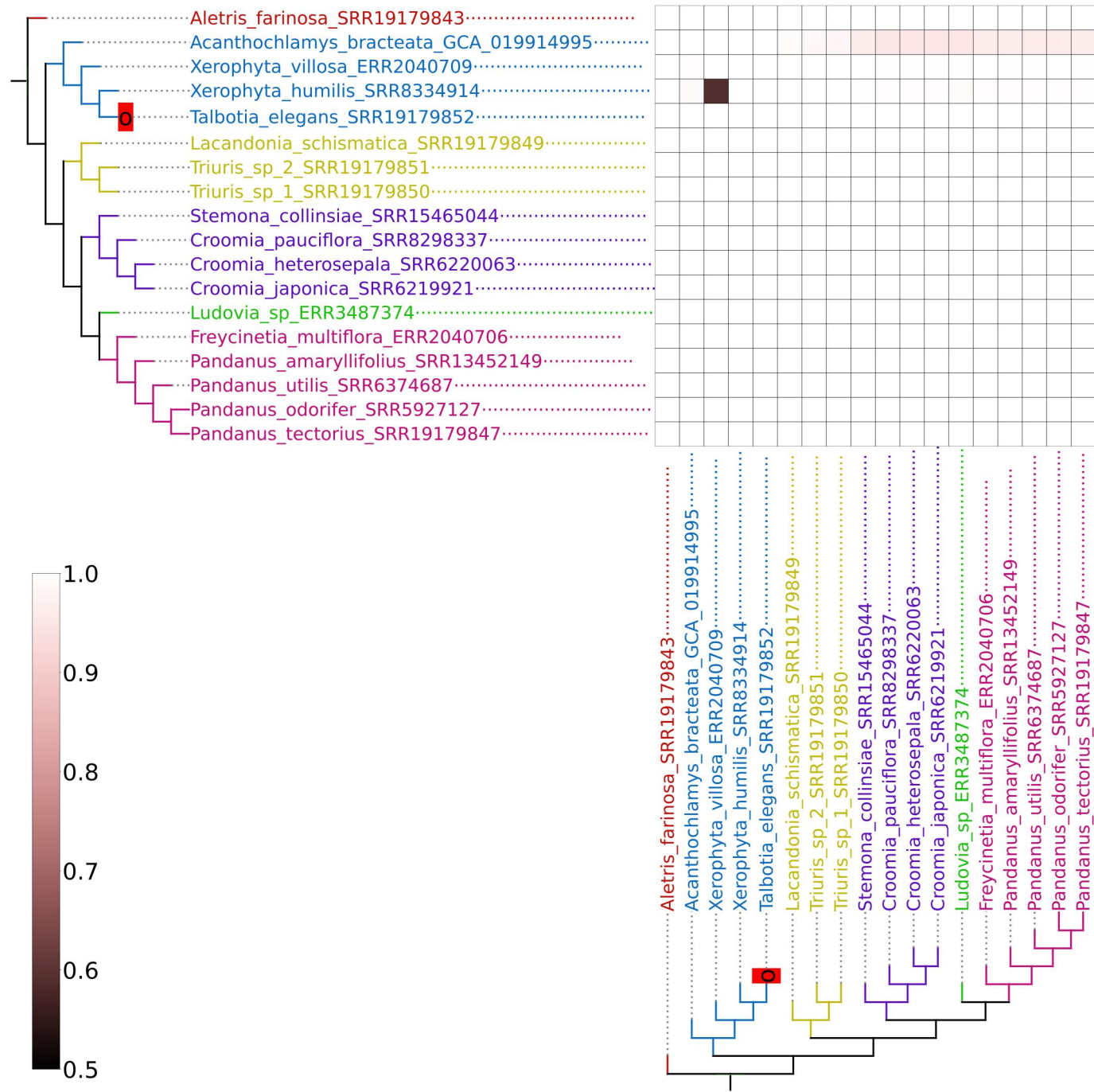

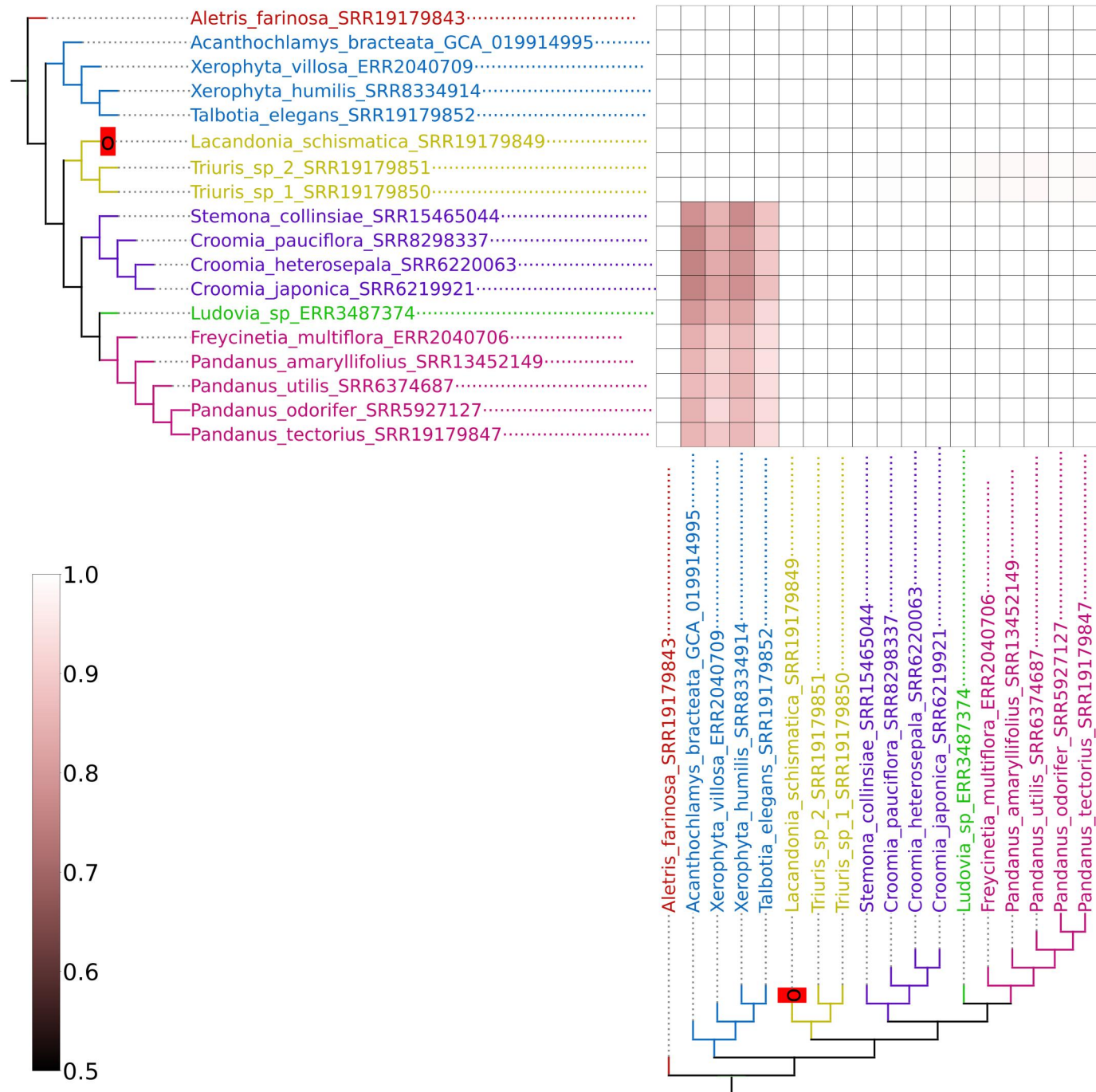

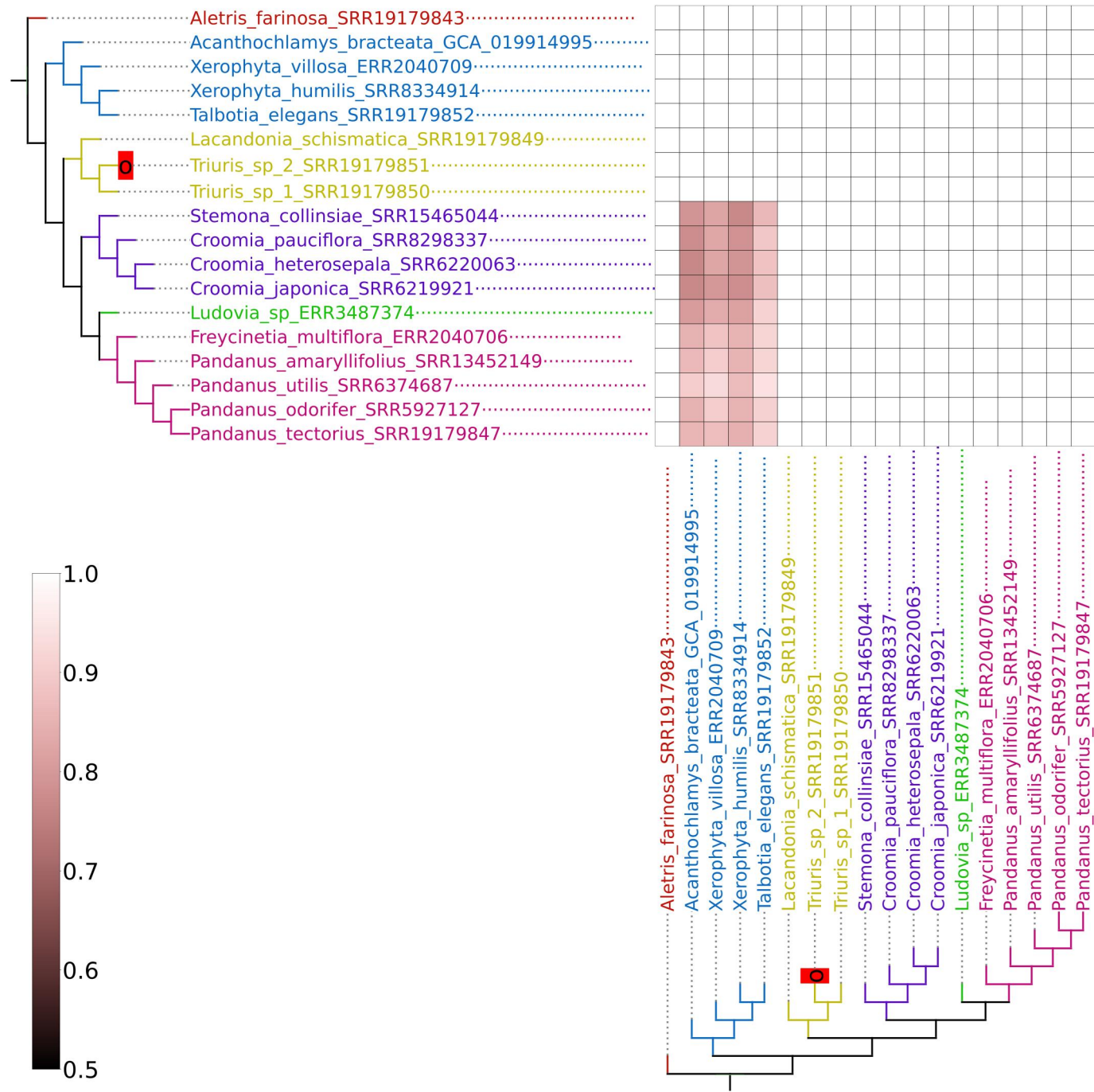

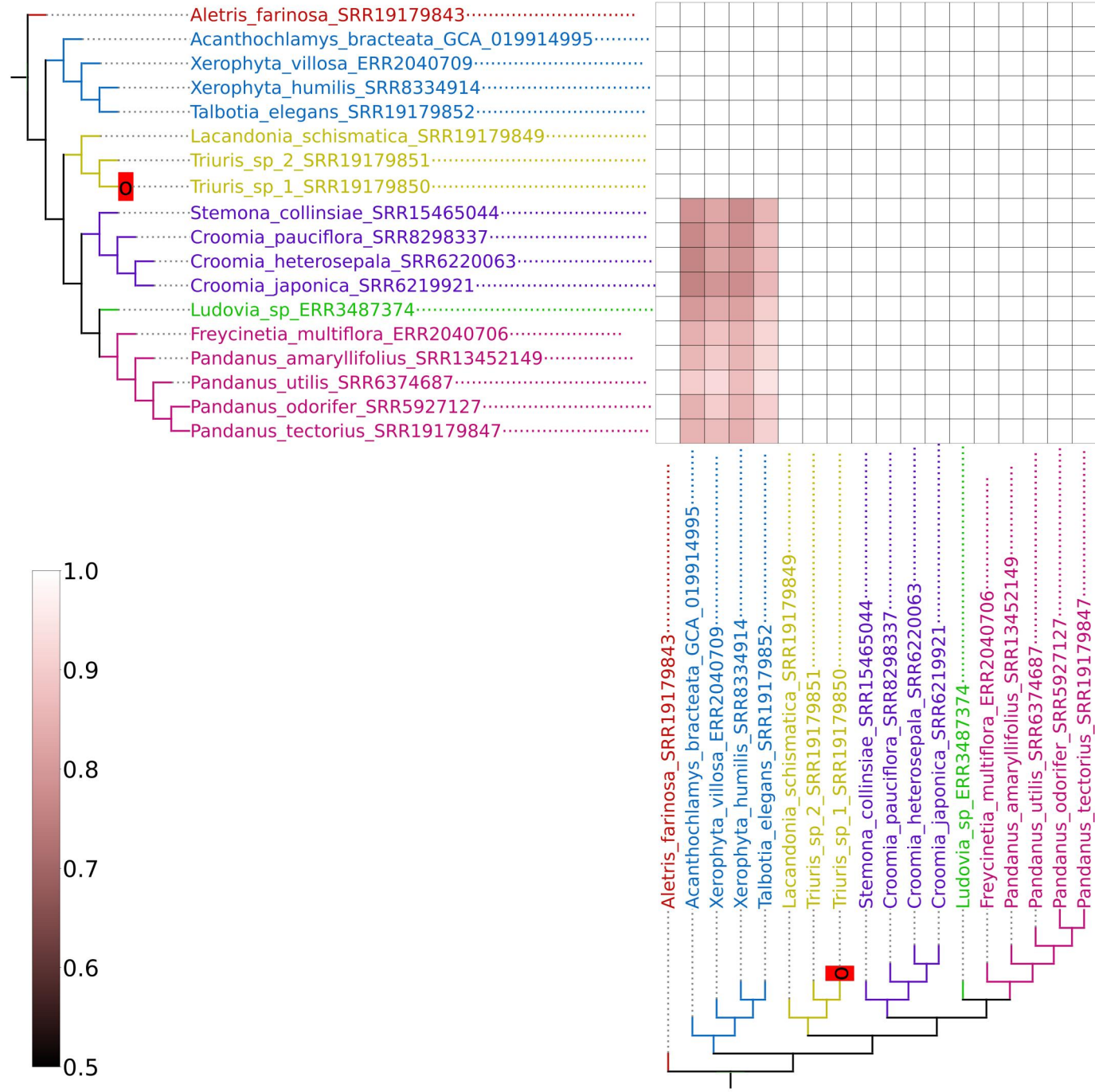

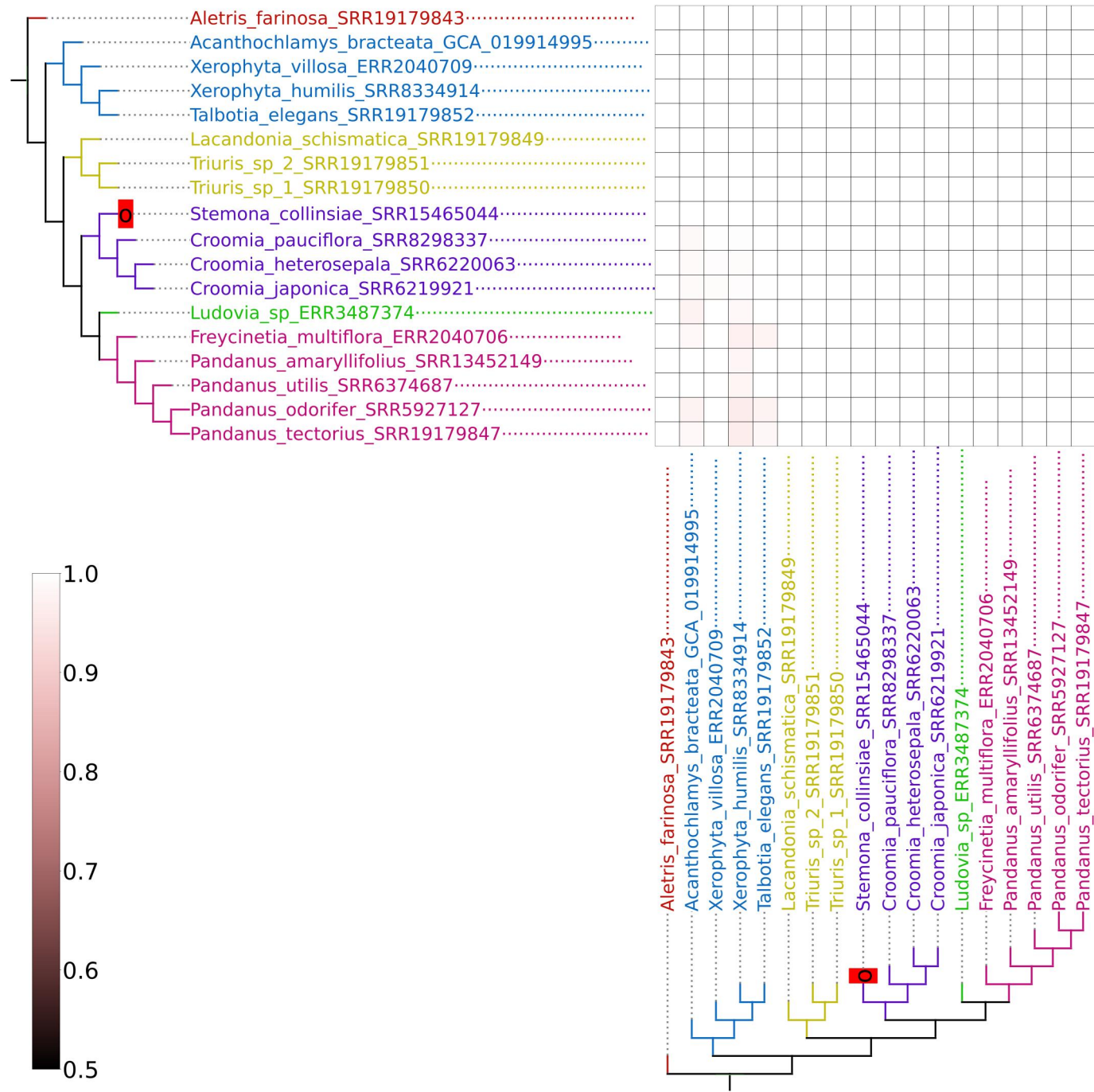

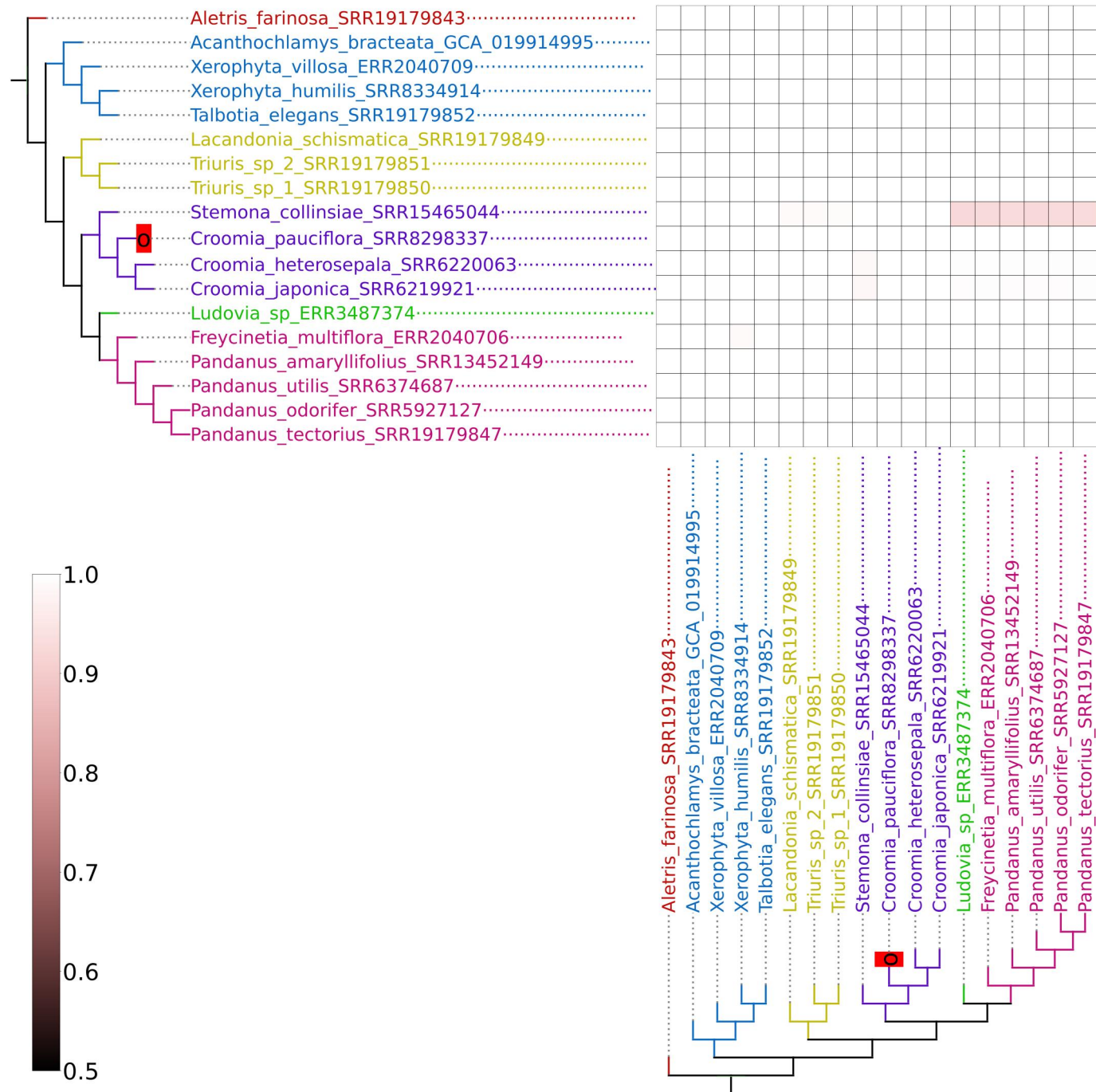

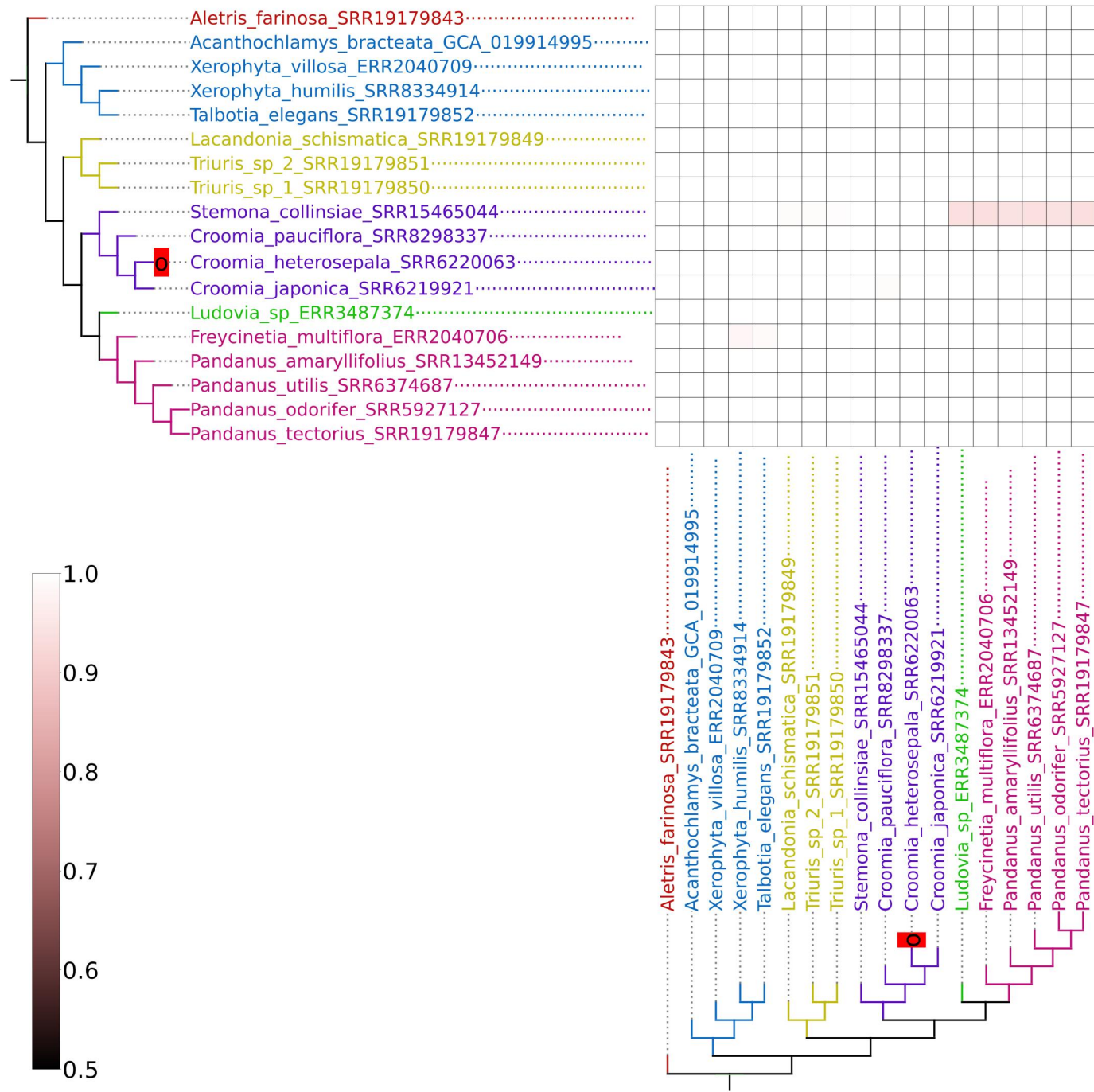

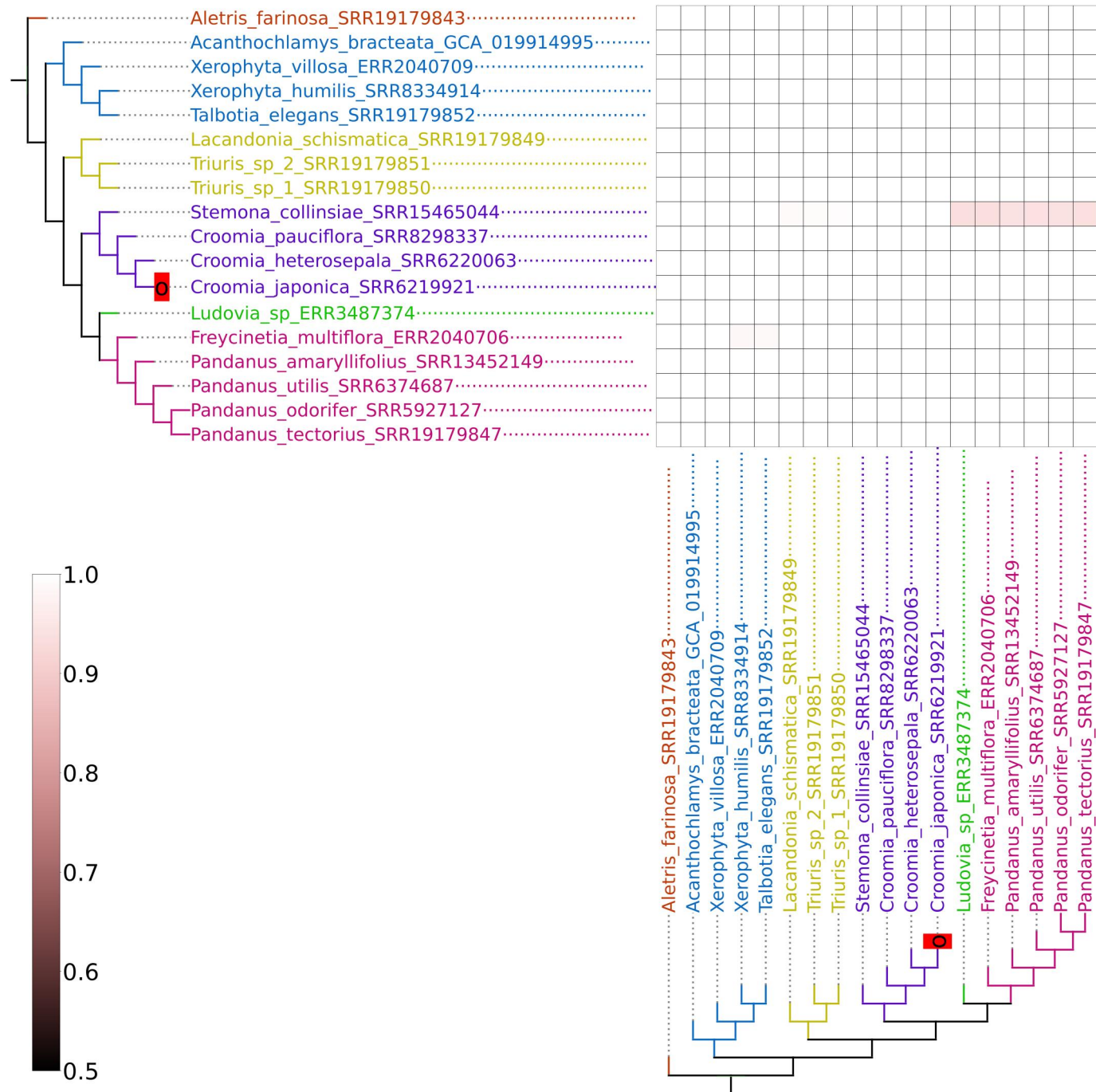

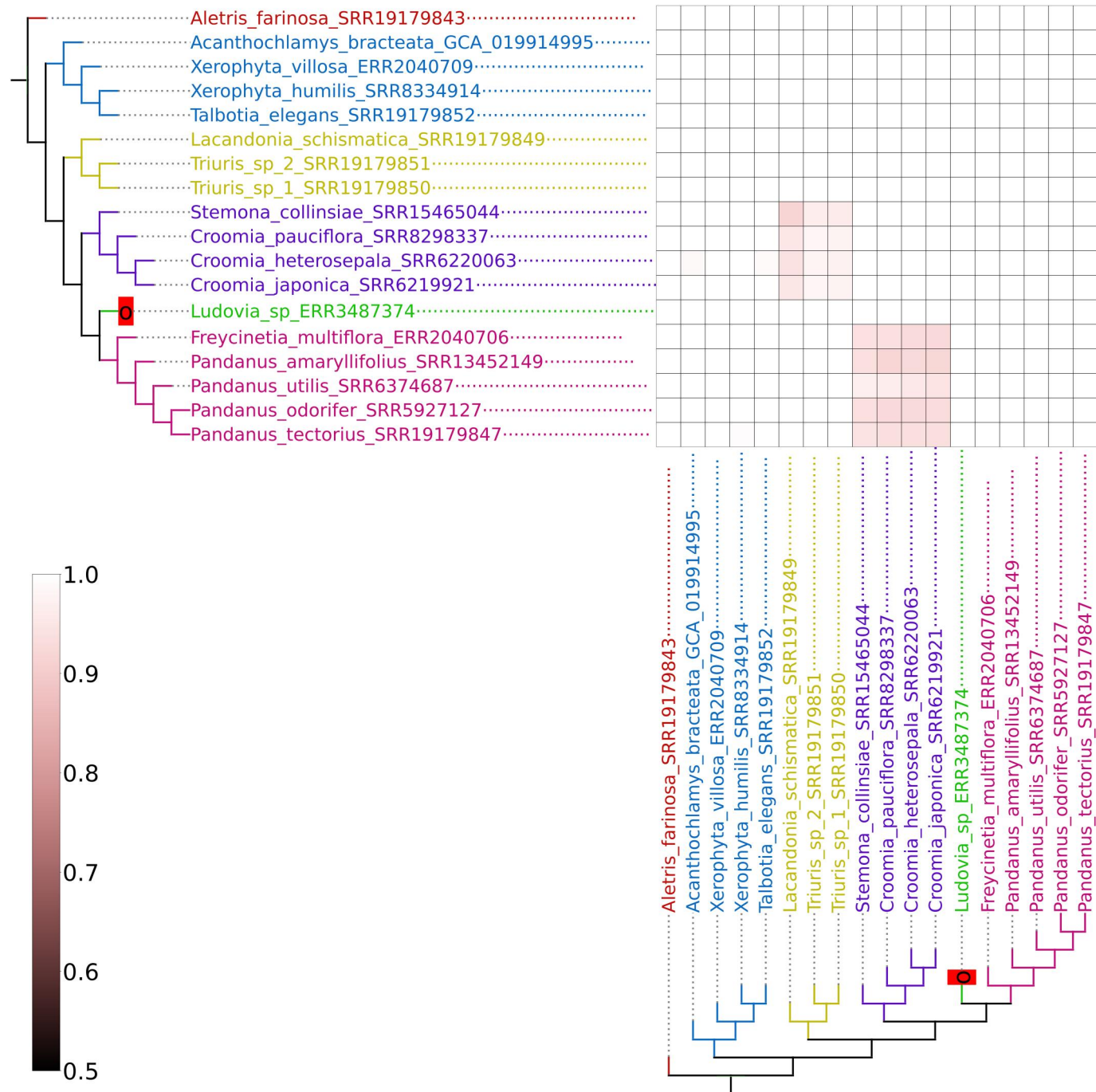

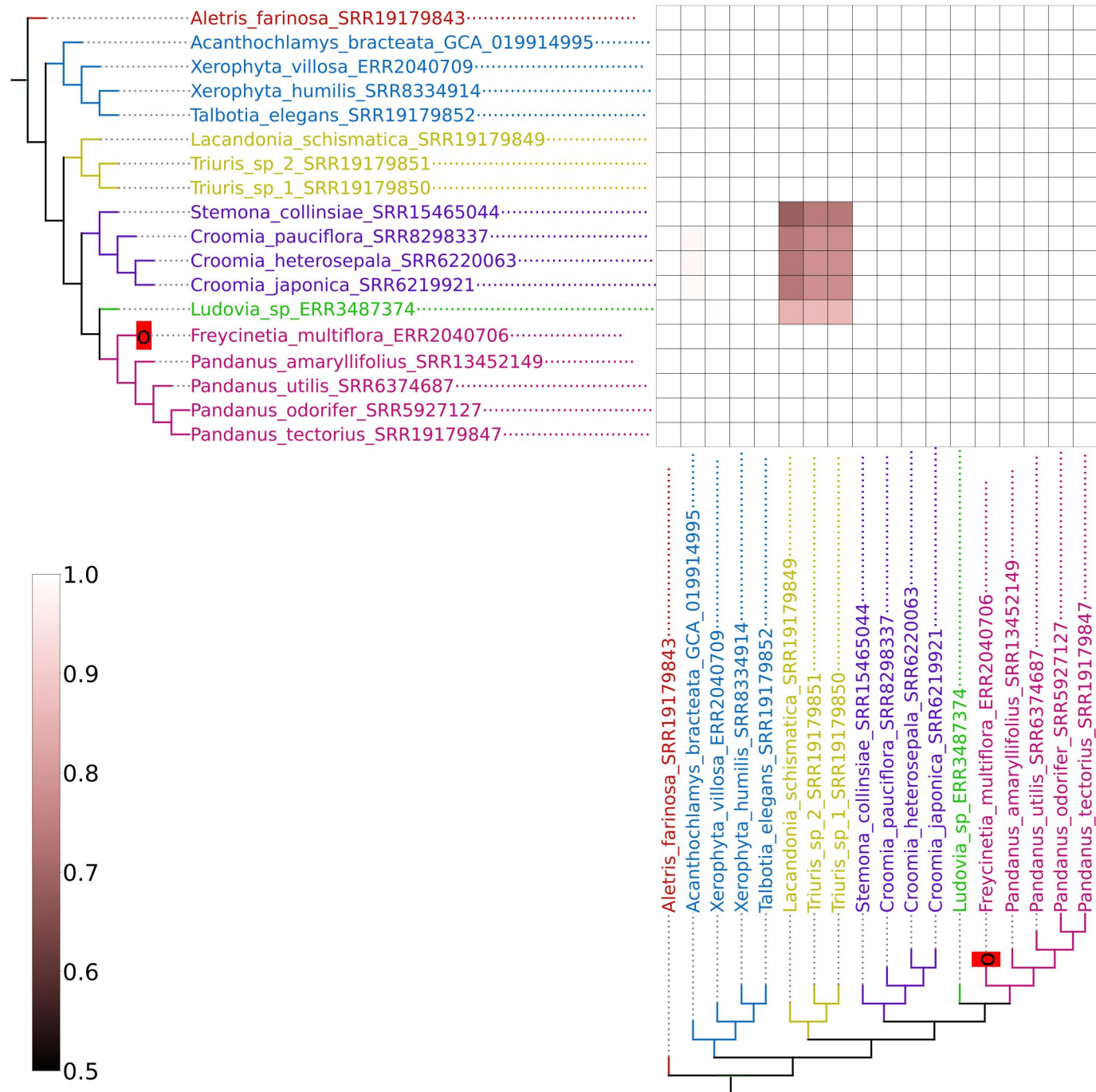

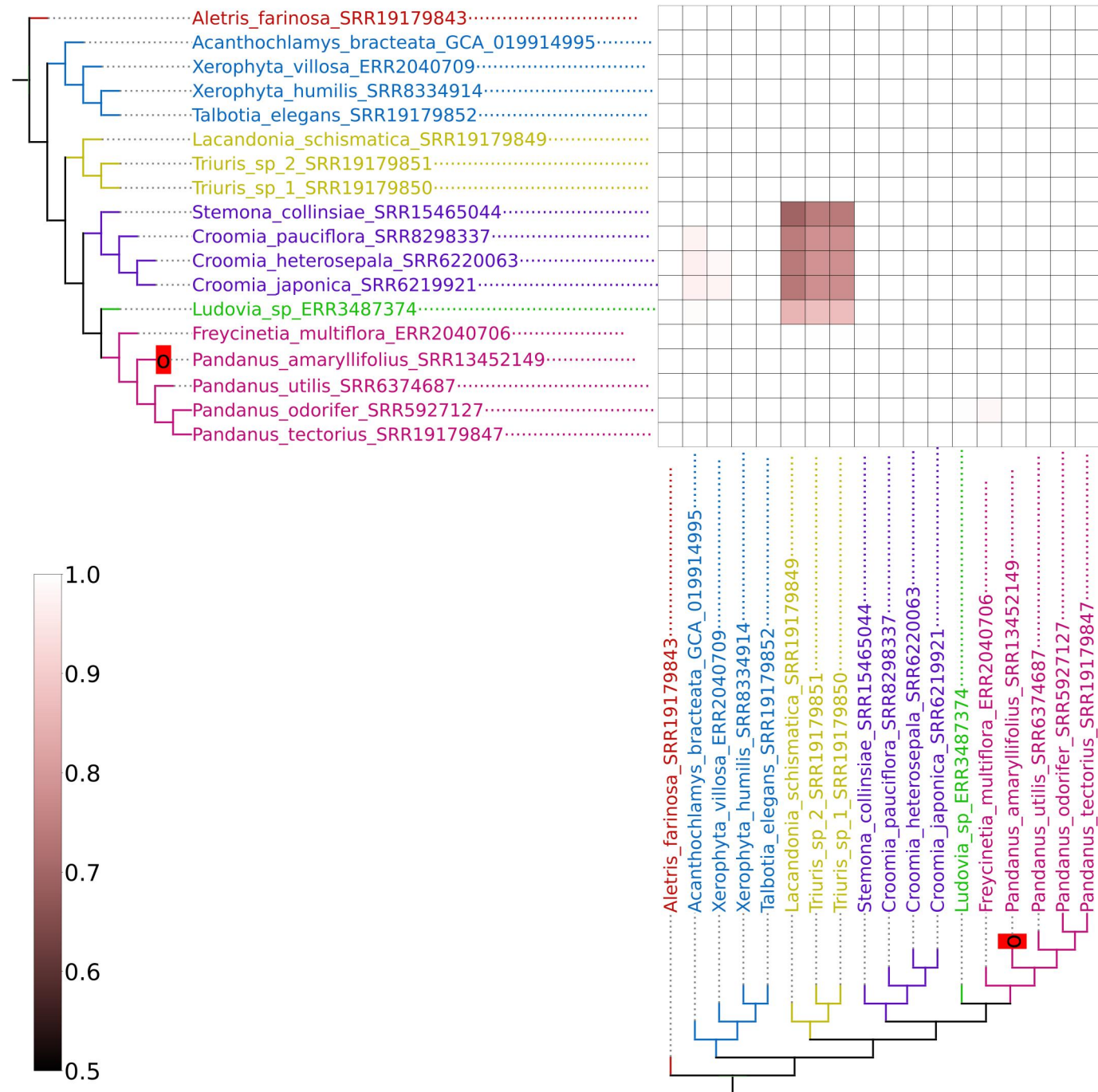

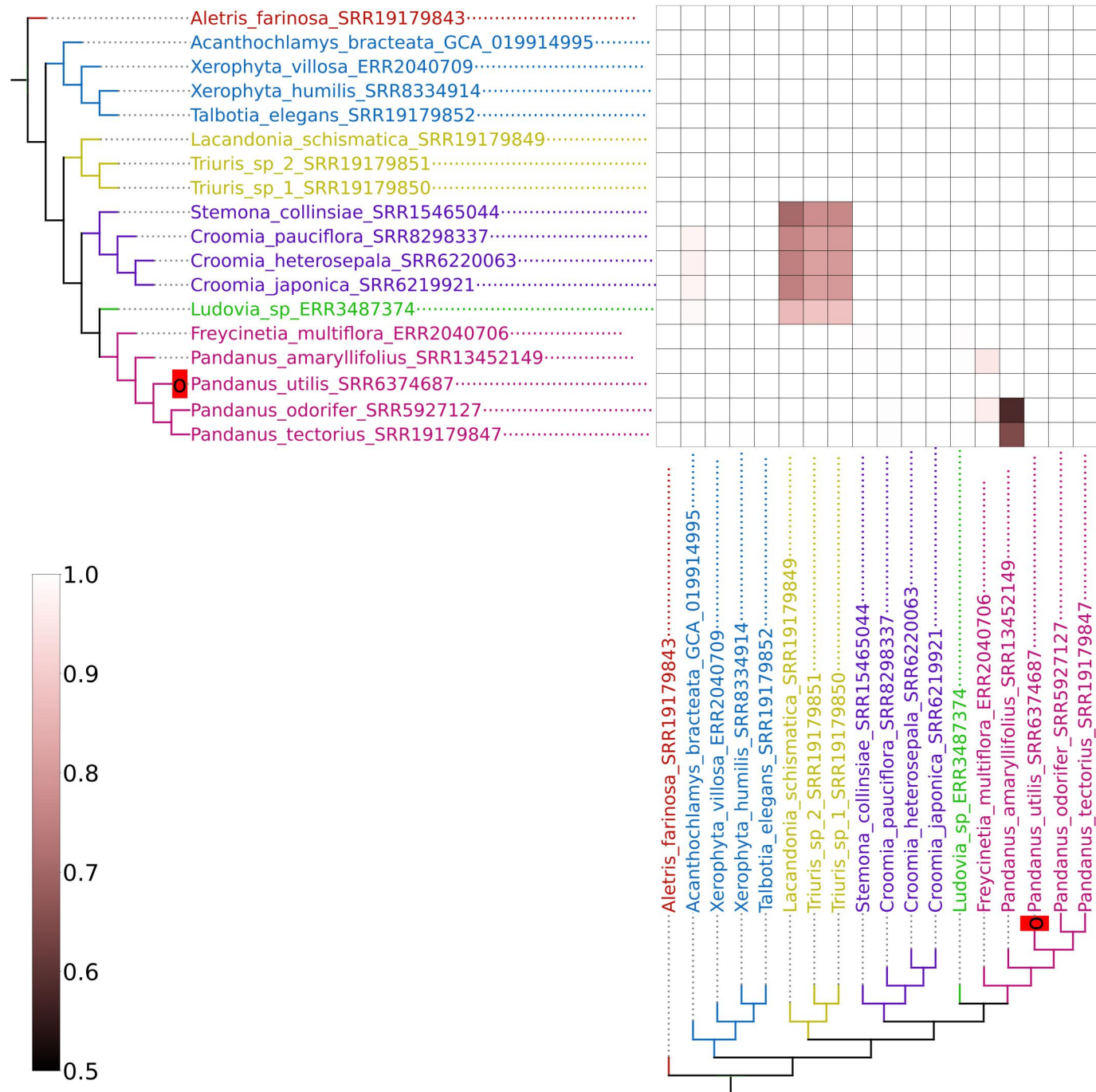

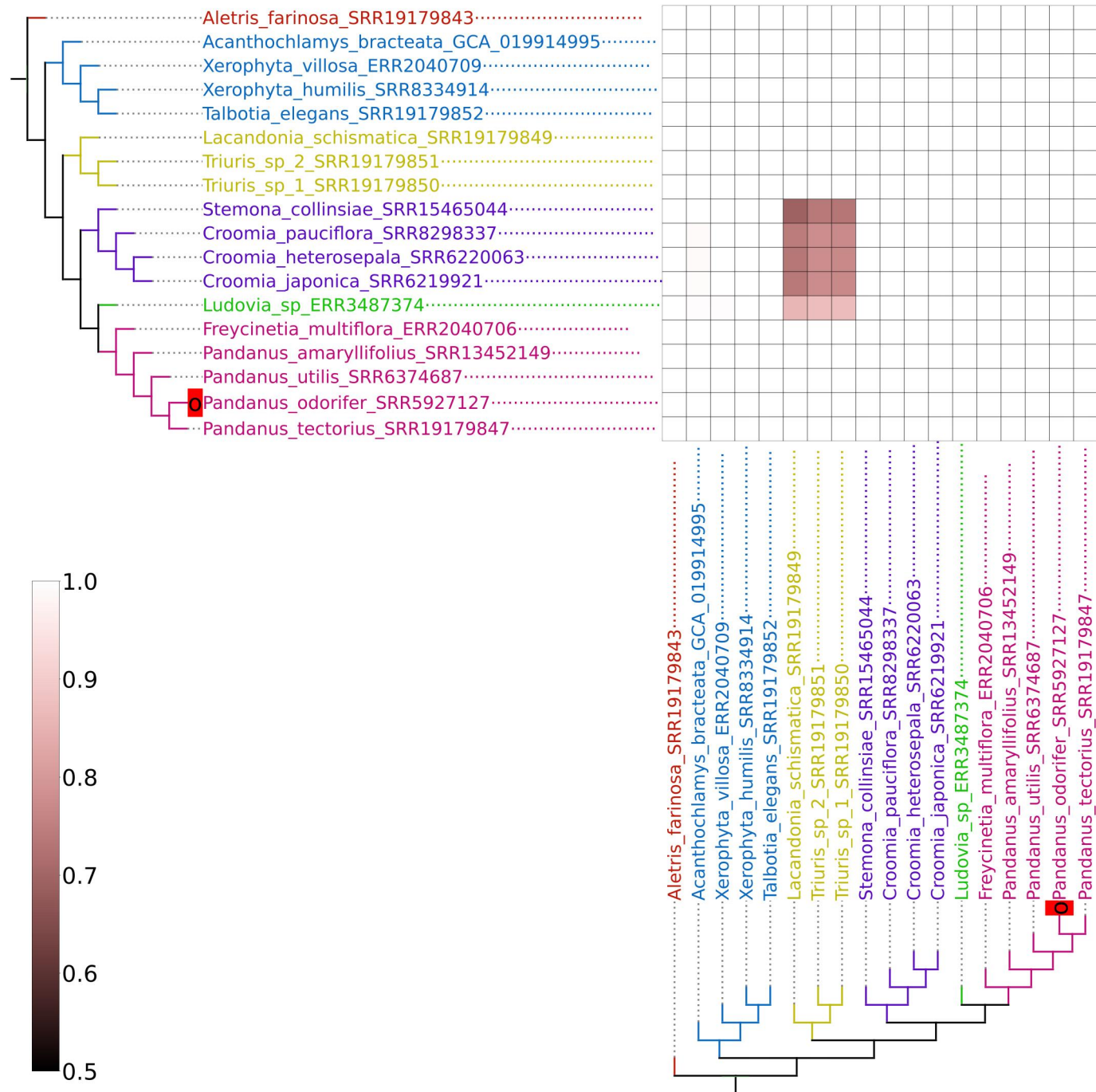

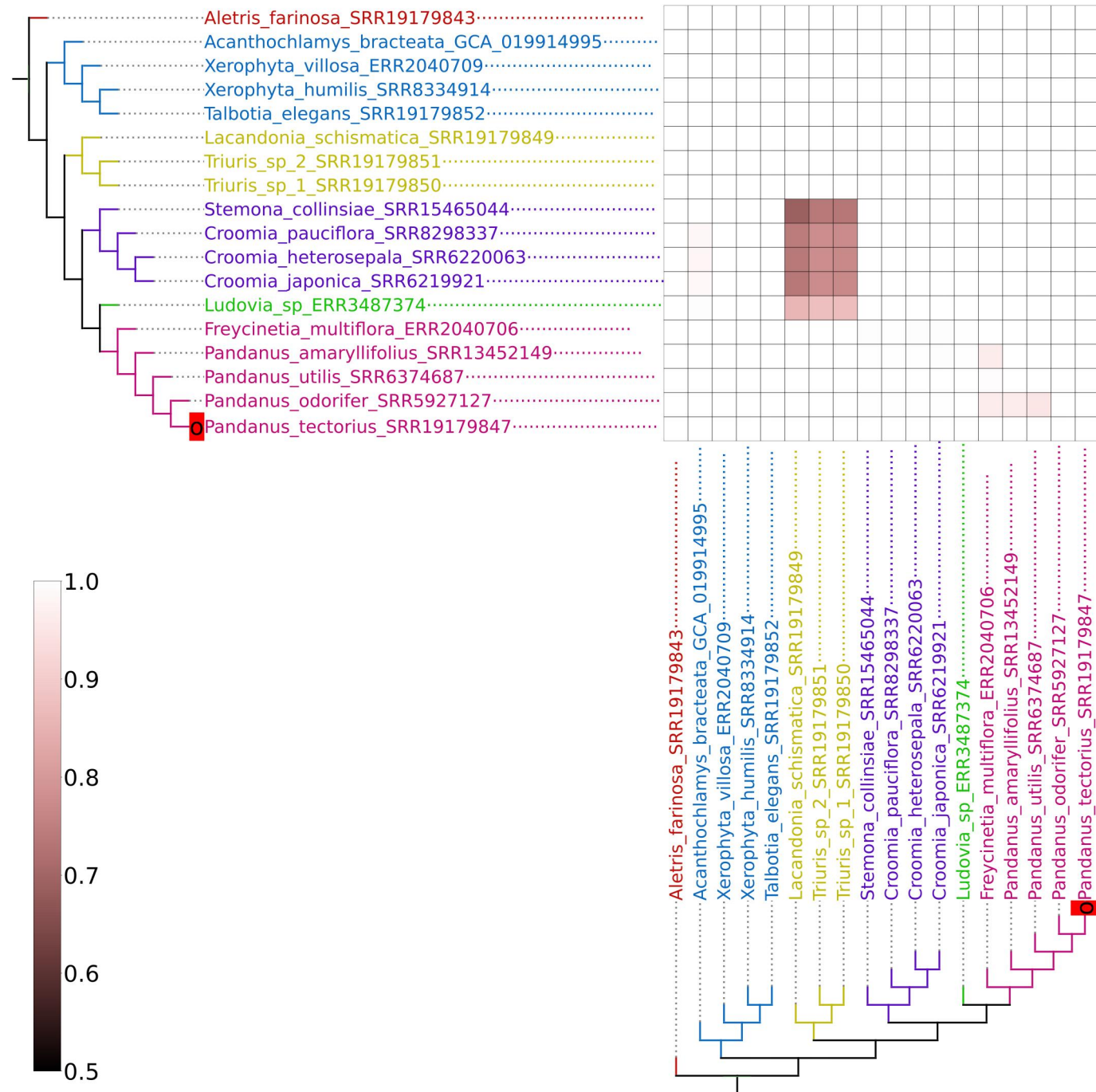

Supplement: Supplementary Figure 6 — Gene flow detection based on the results of HyDe, with heatmaps drawn for each sample in Pandanales (see Methods 2.7). [file Image6.pdf]

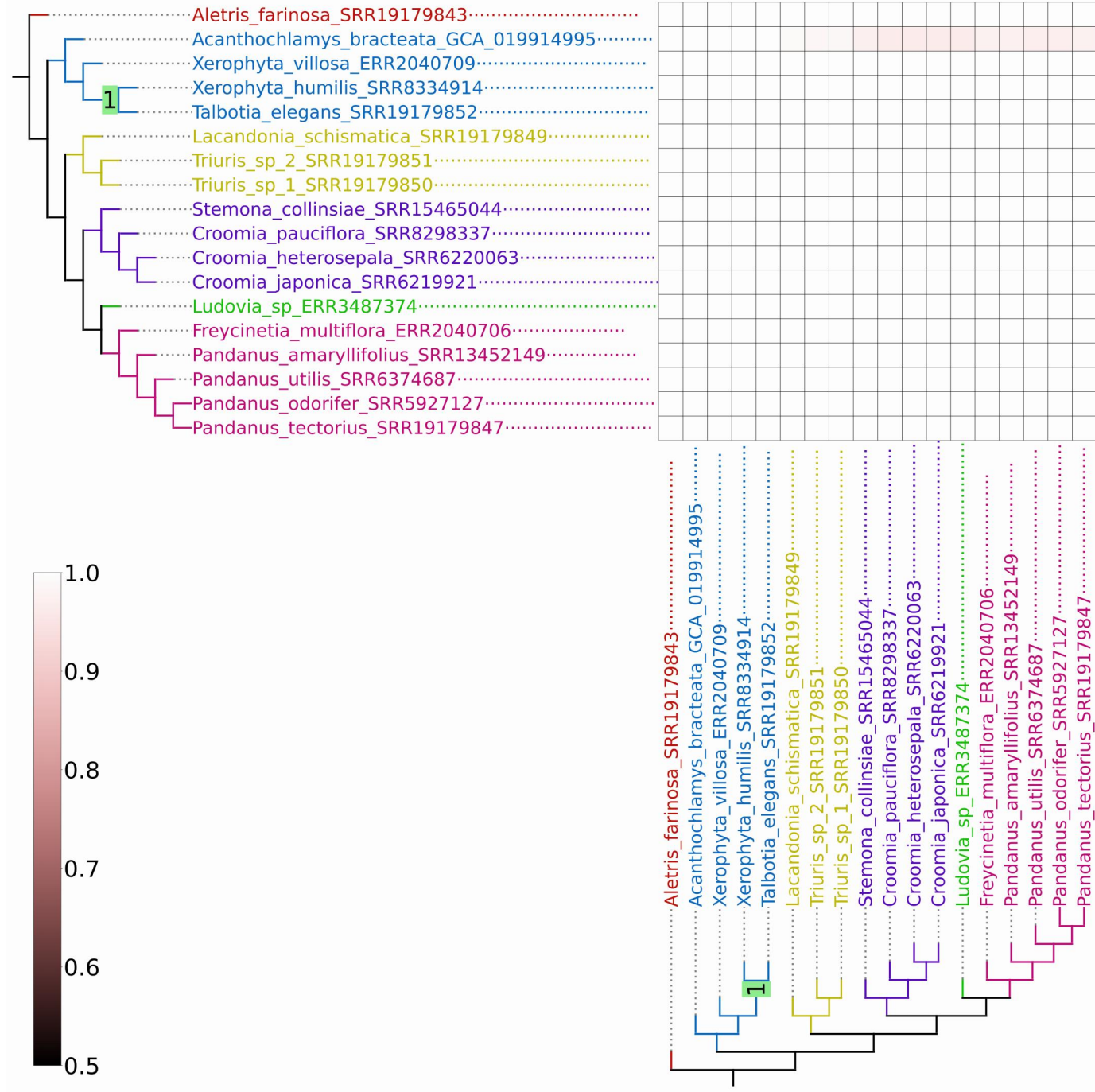

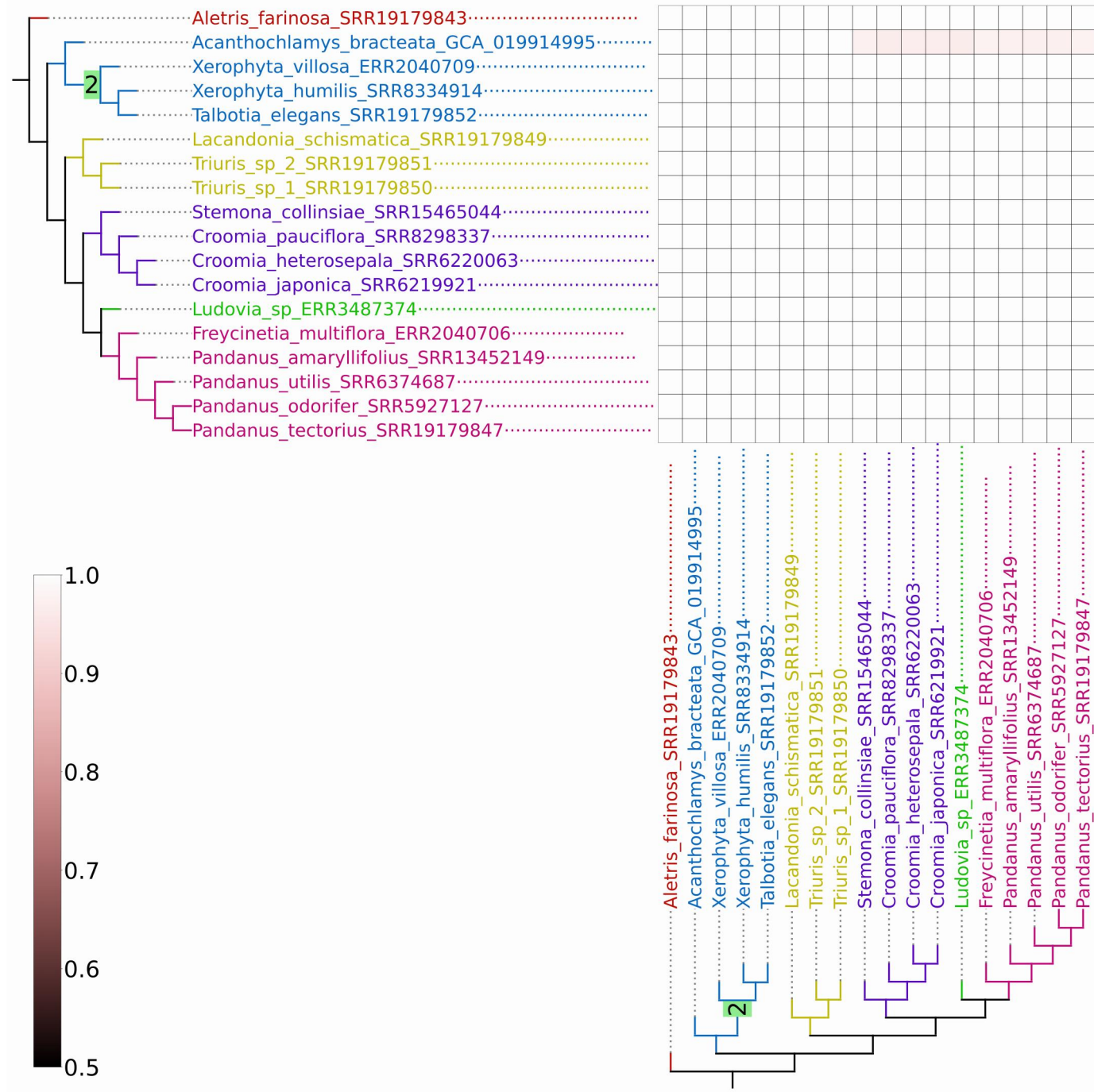

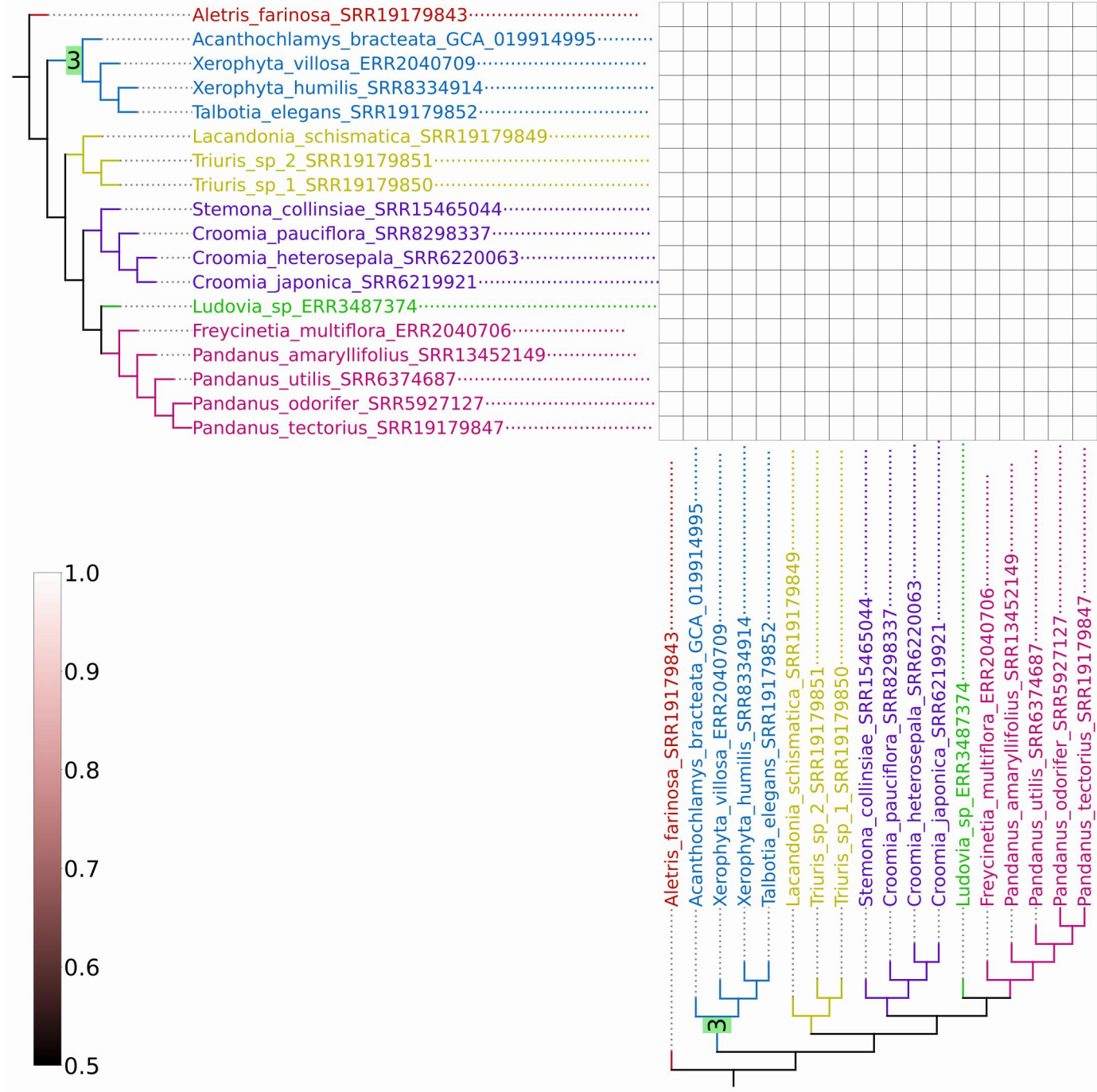

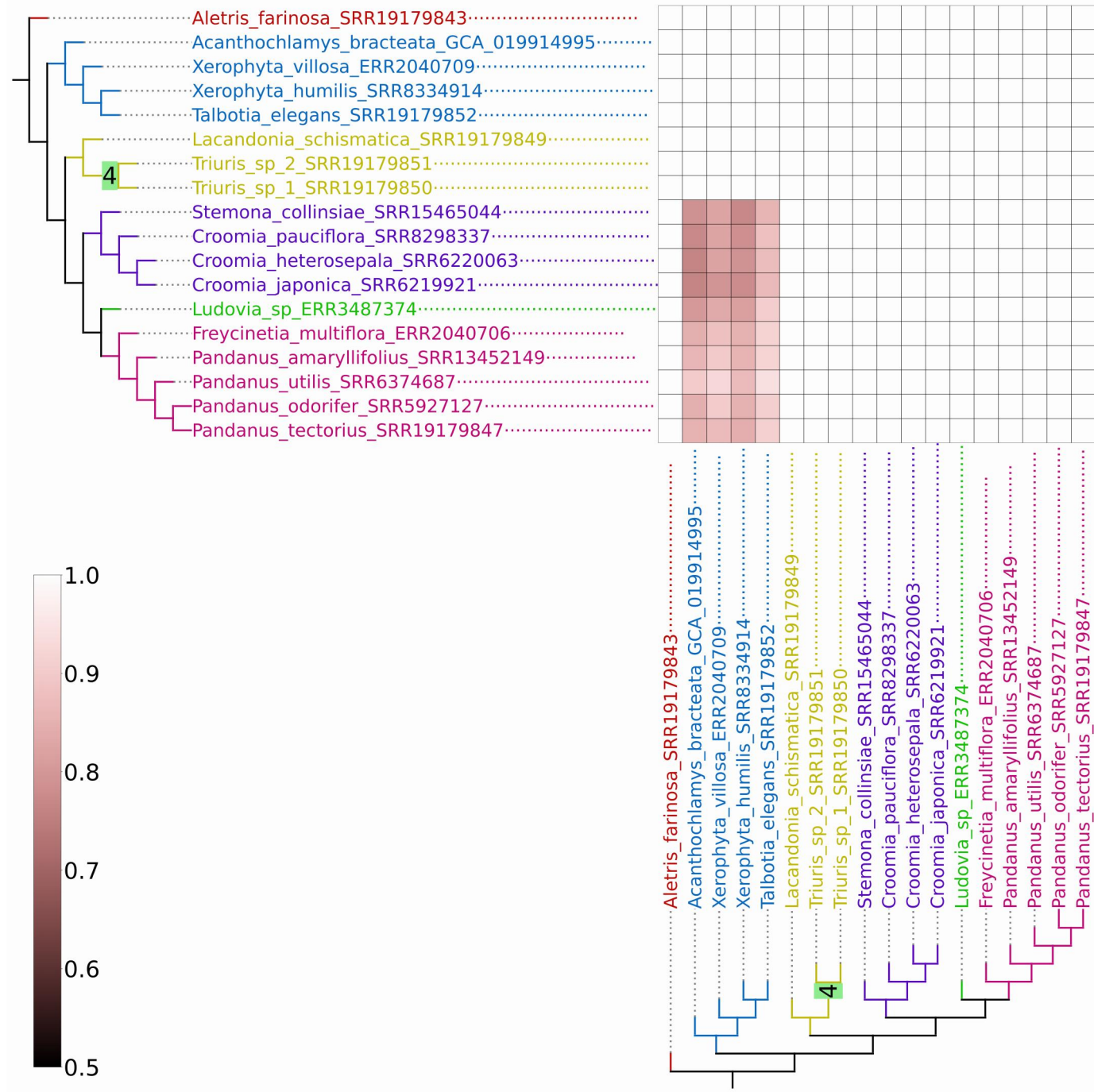

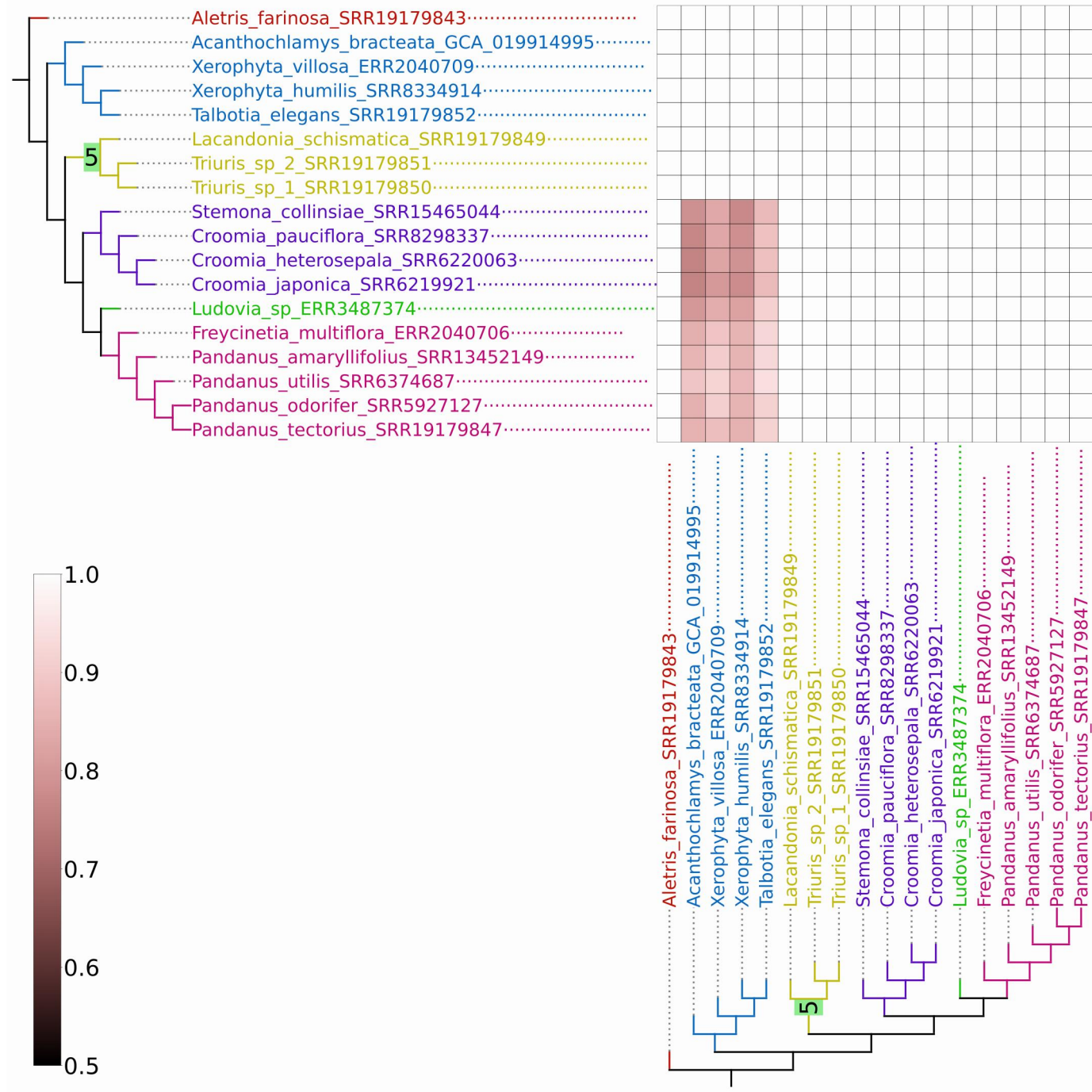

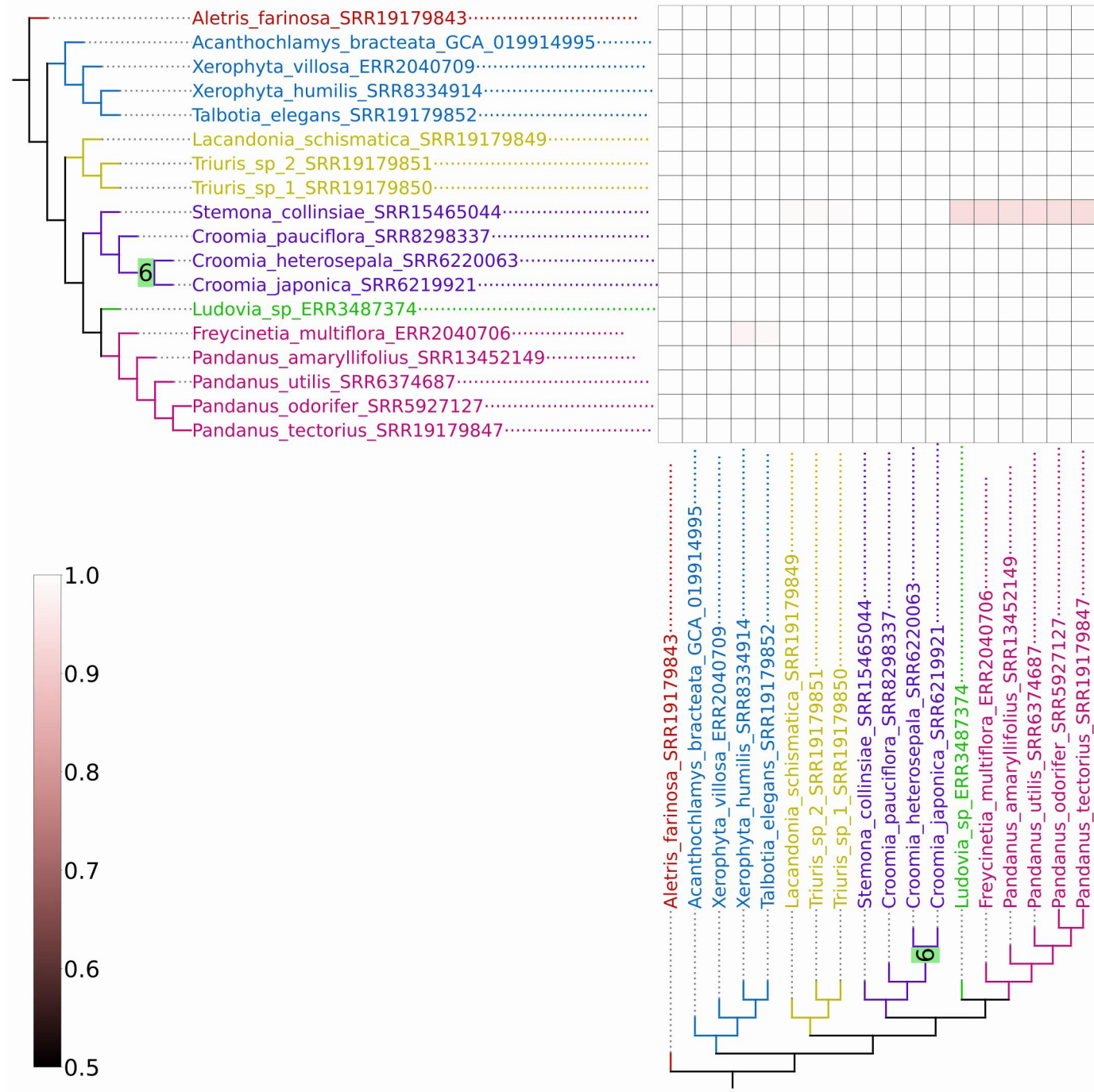

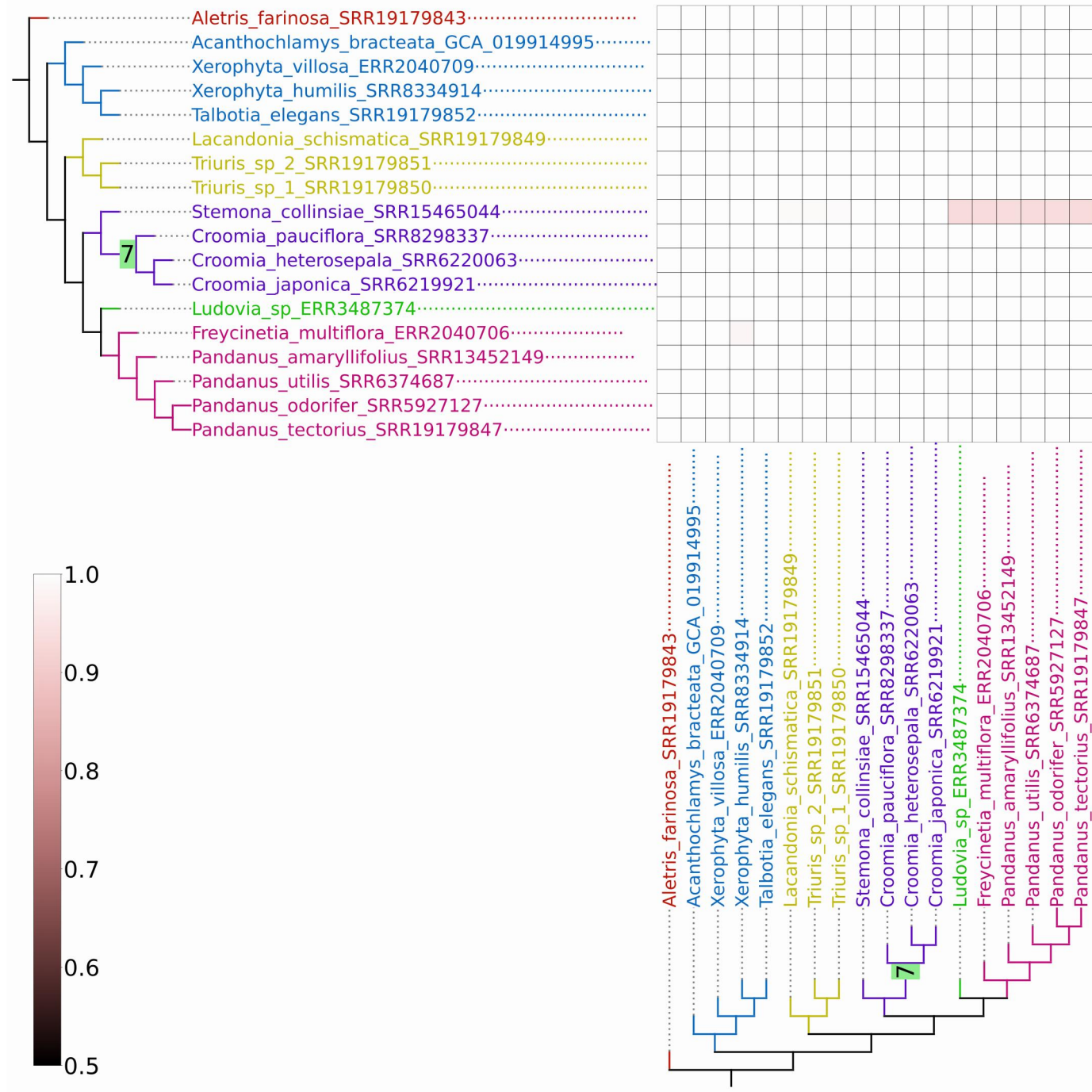

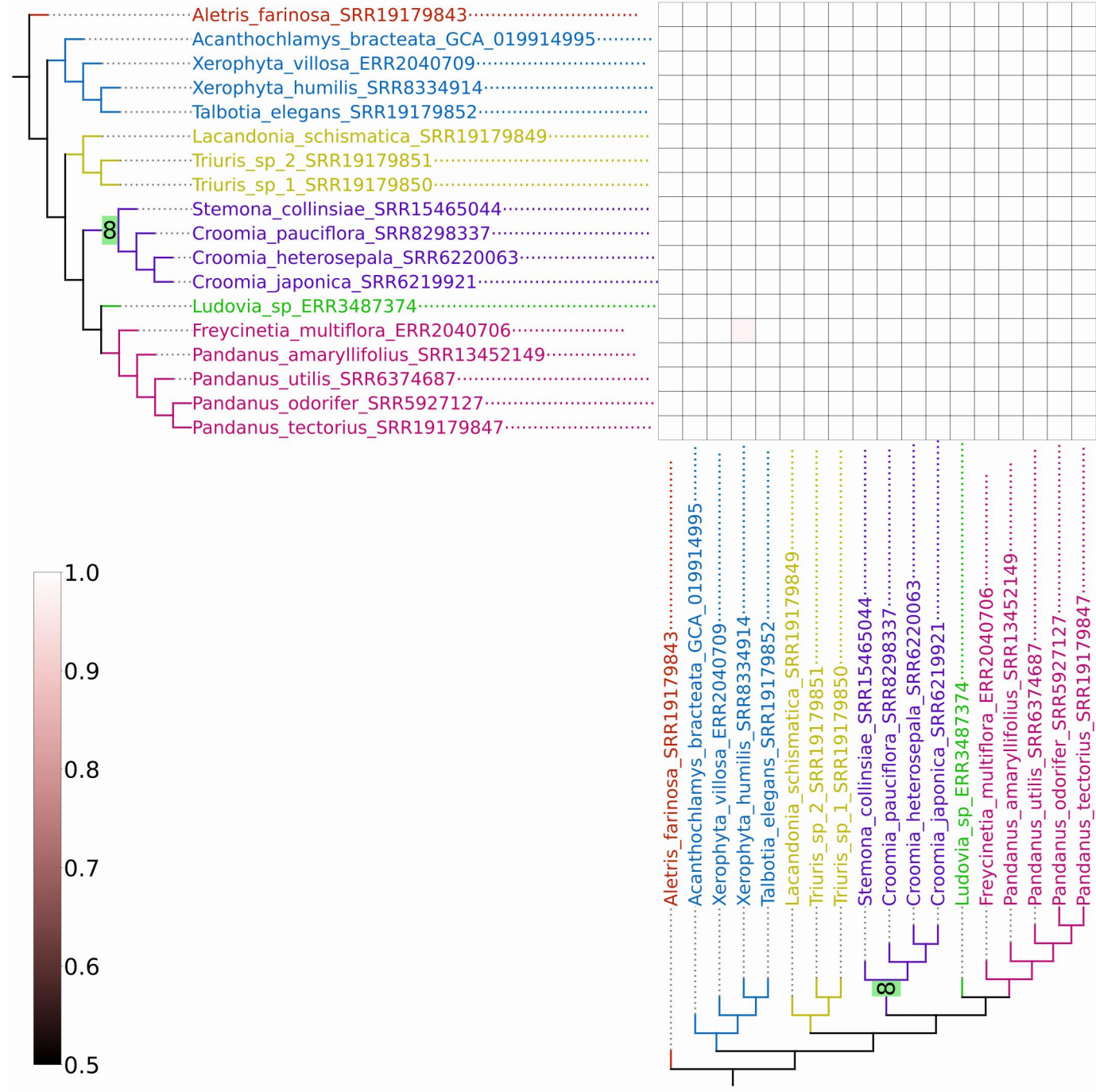

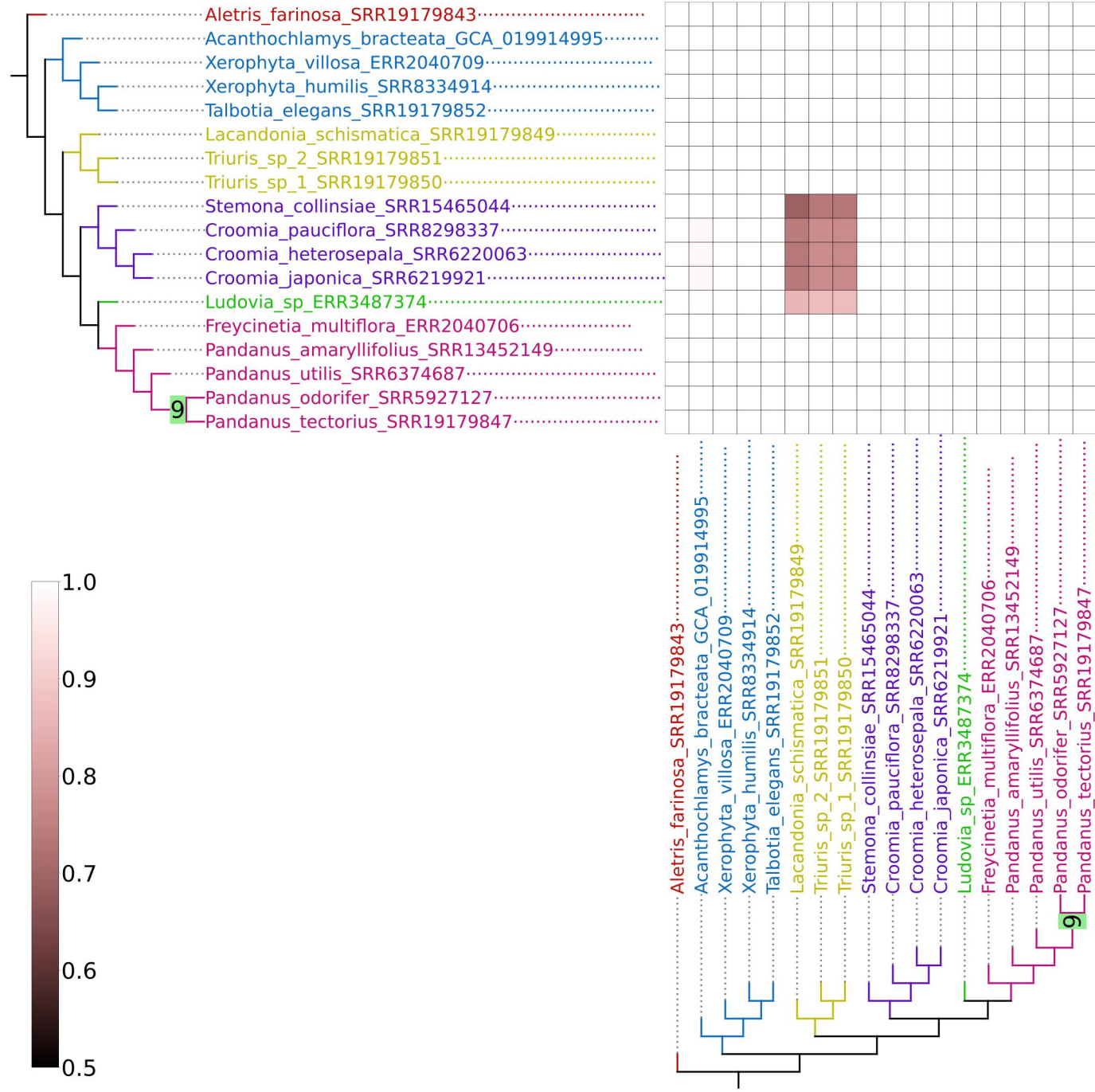

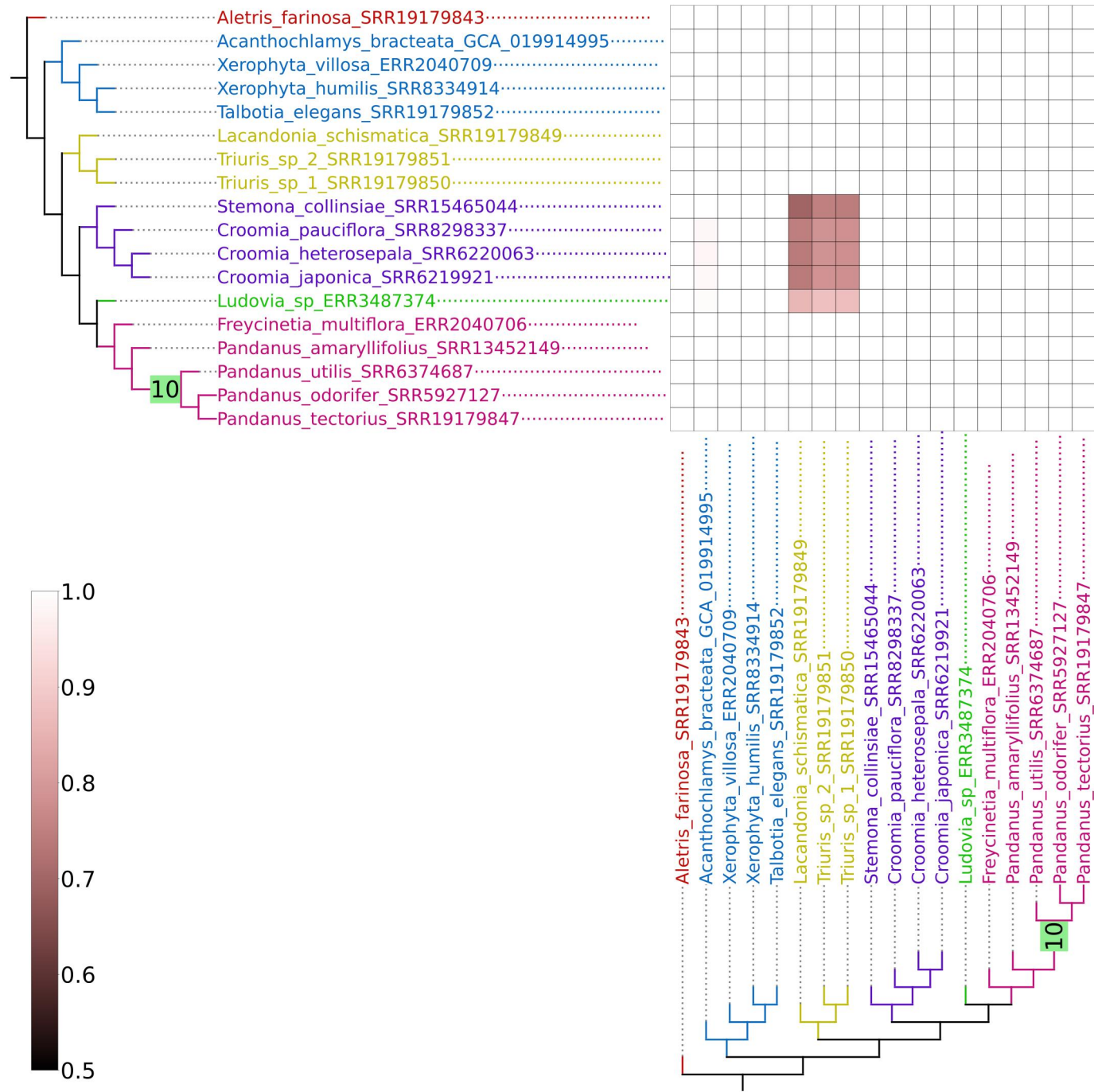

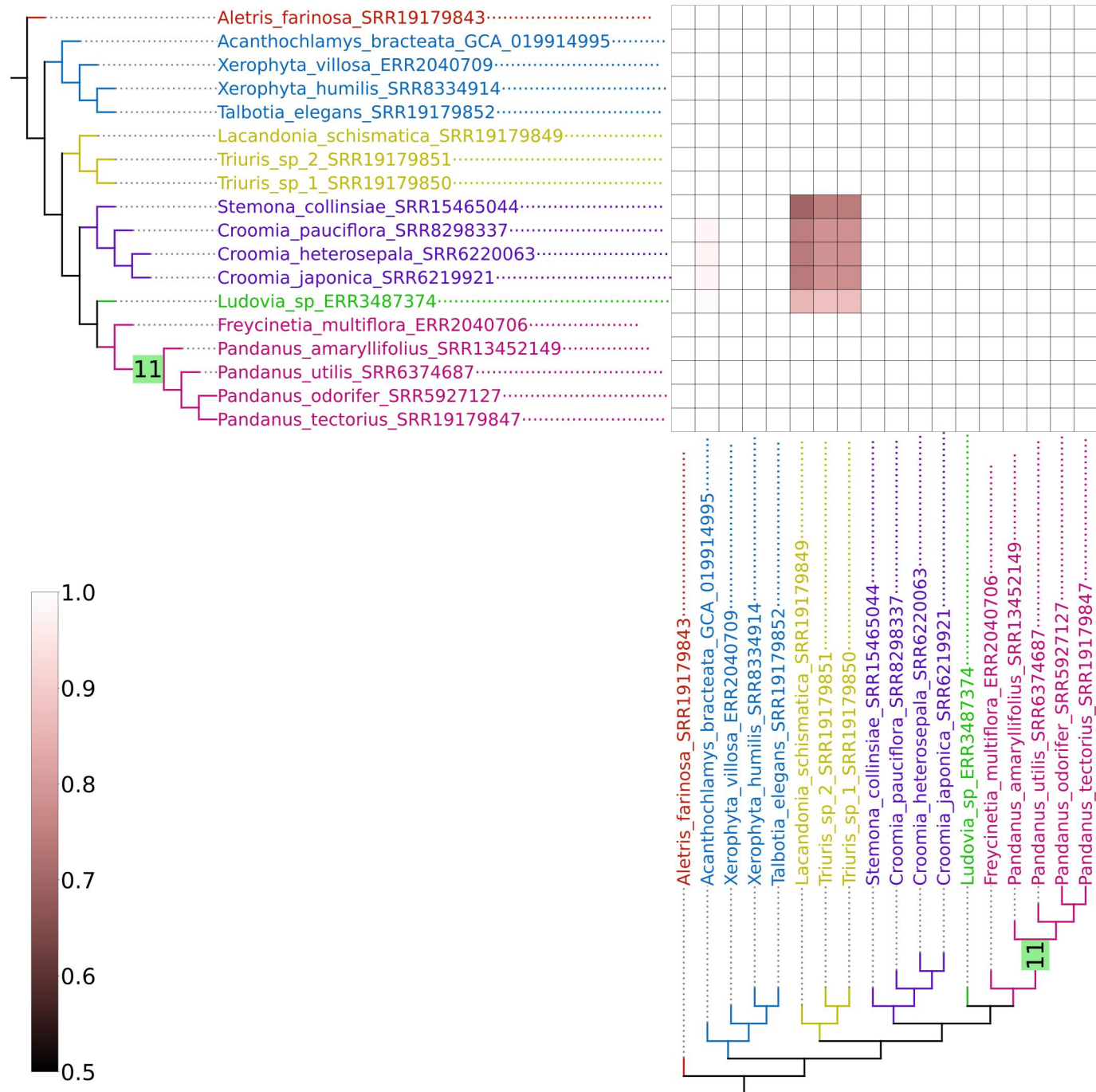

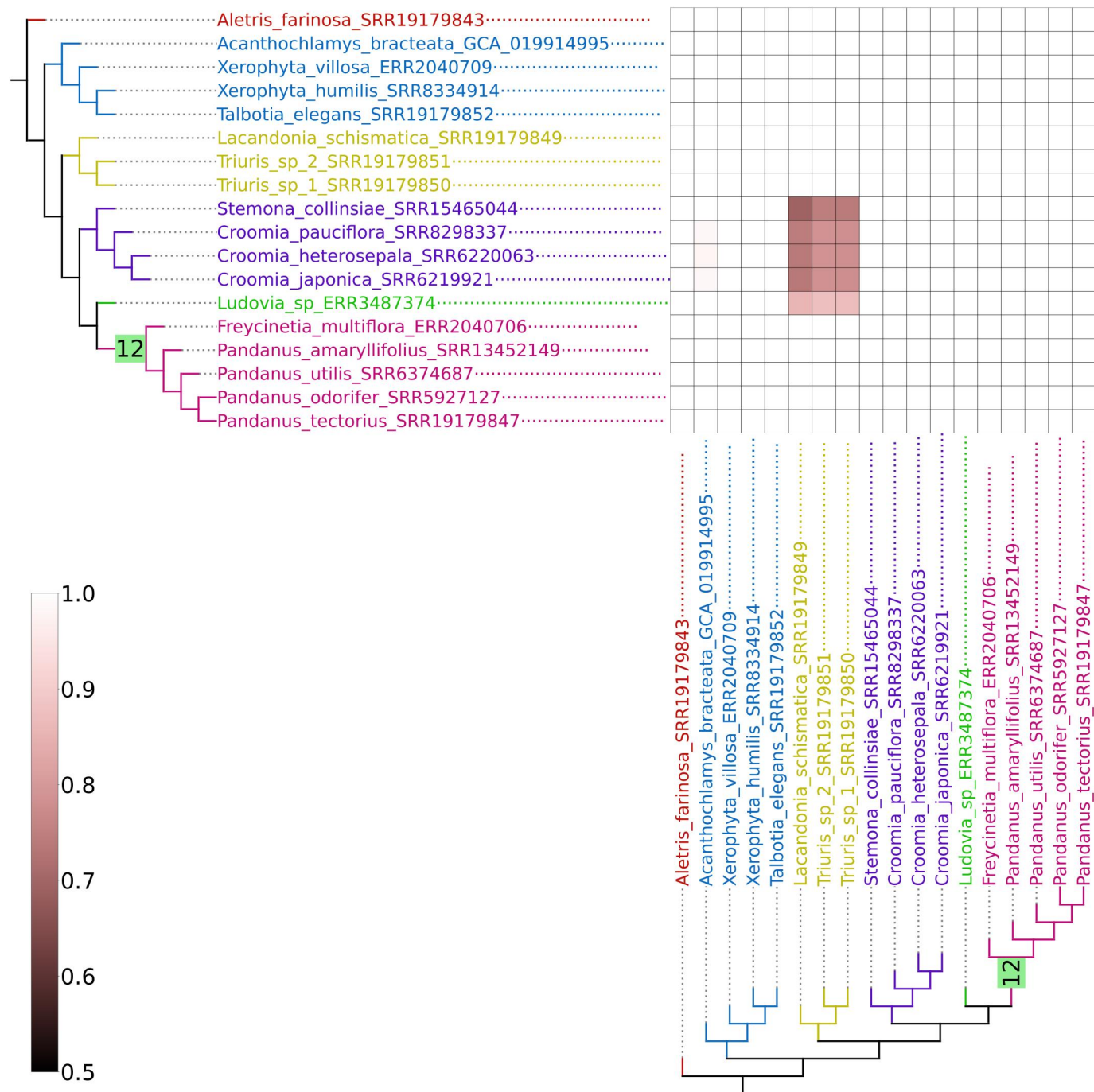

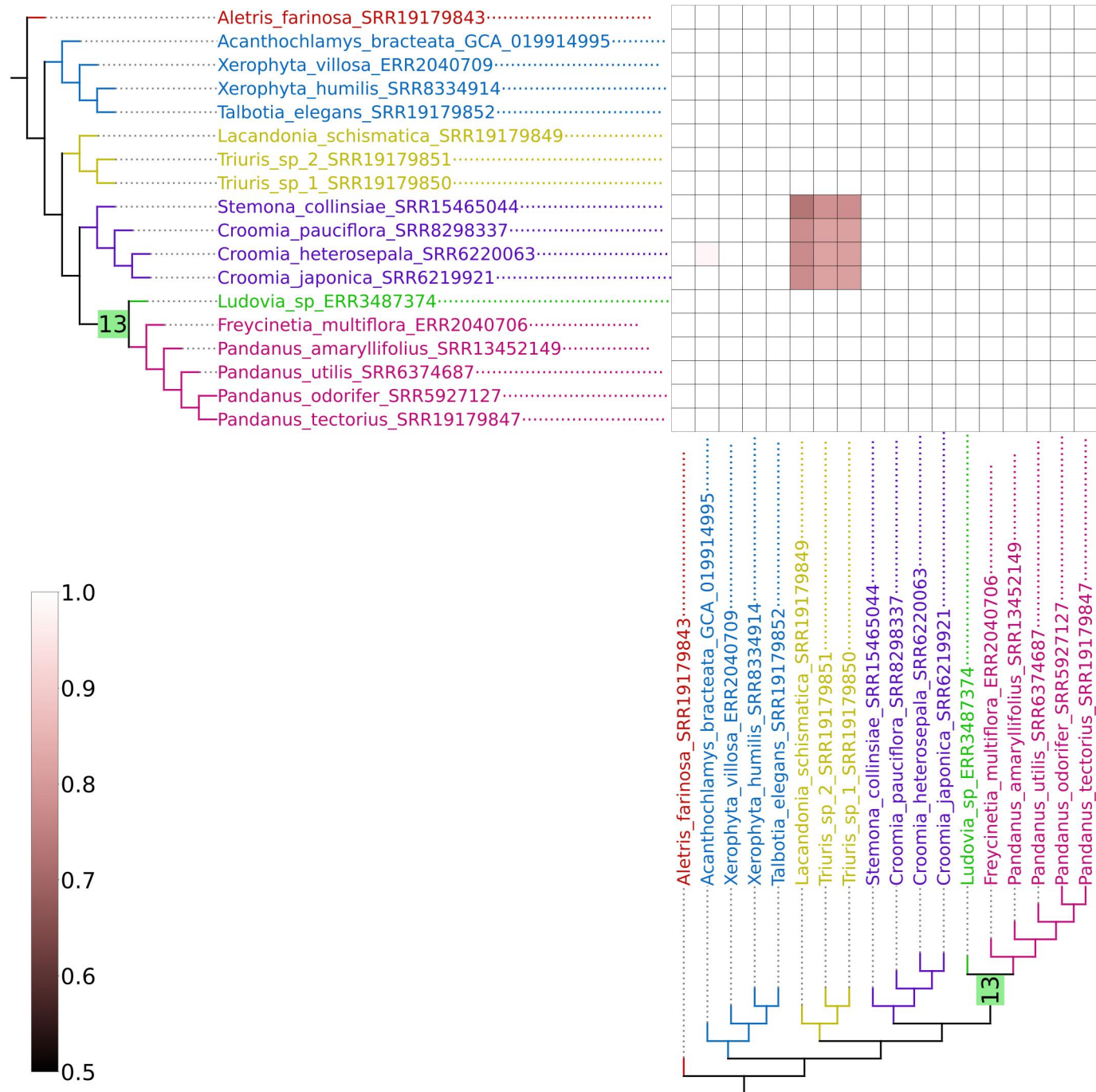

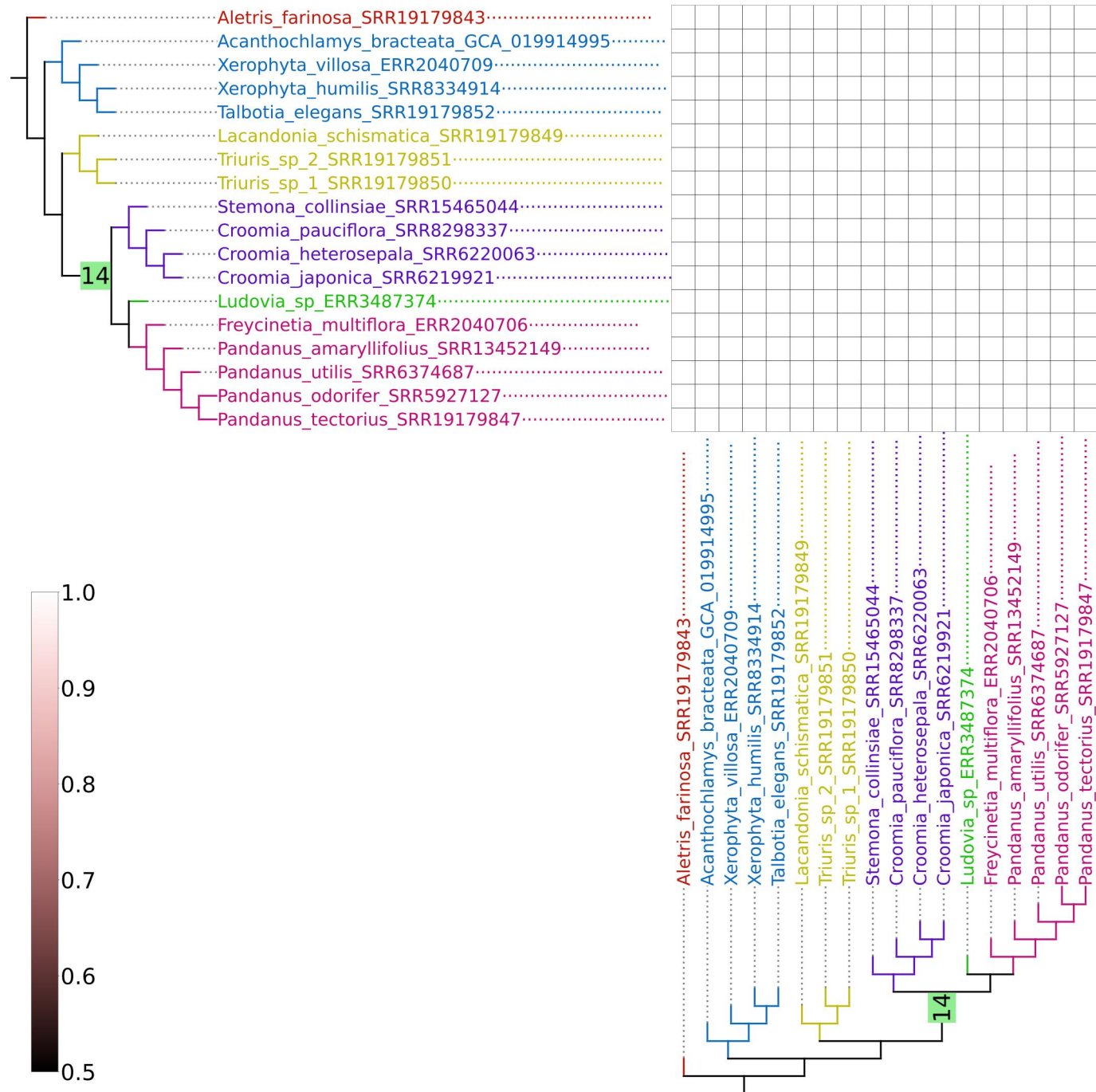

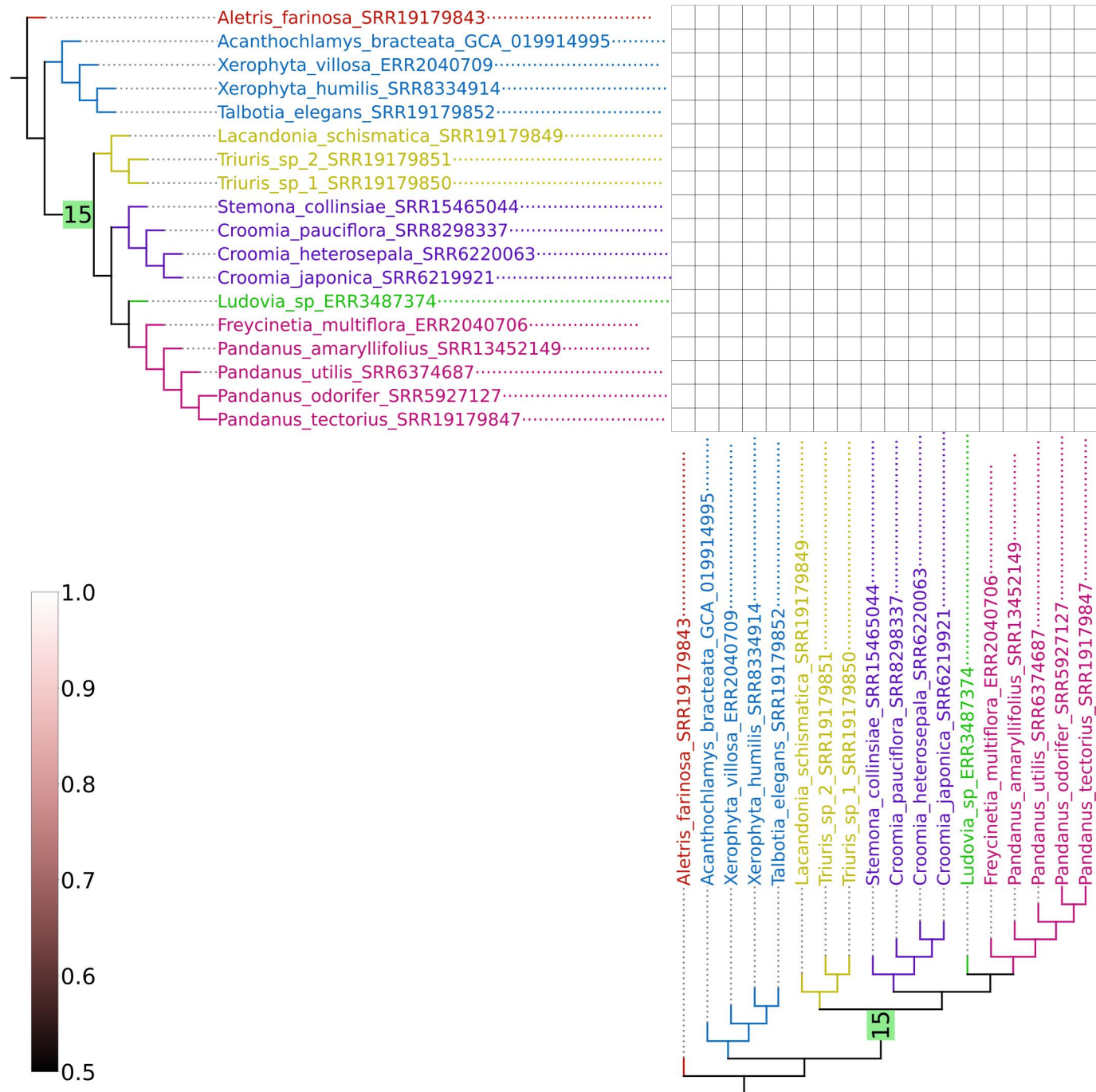

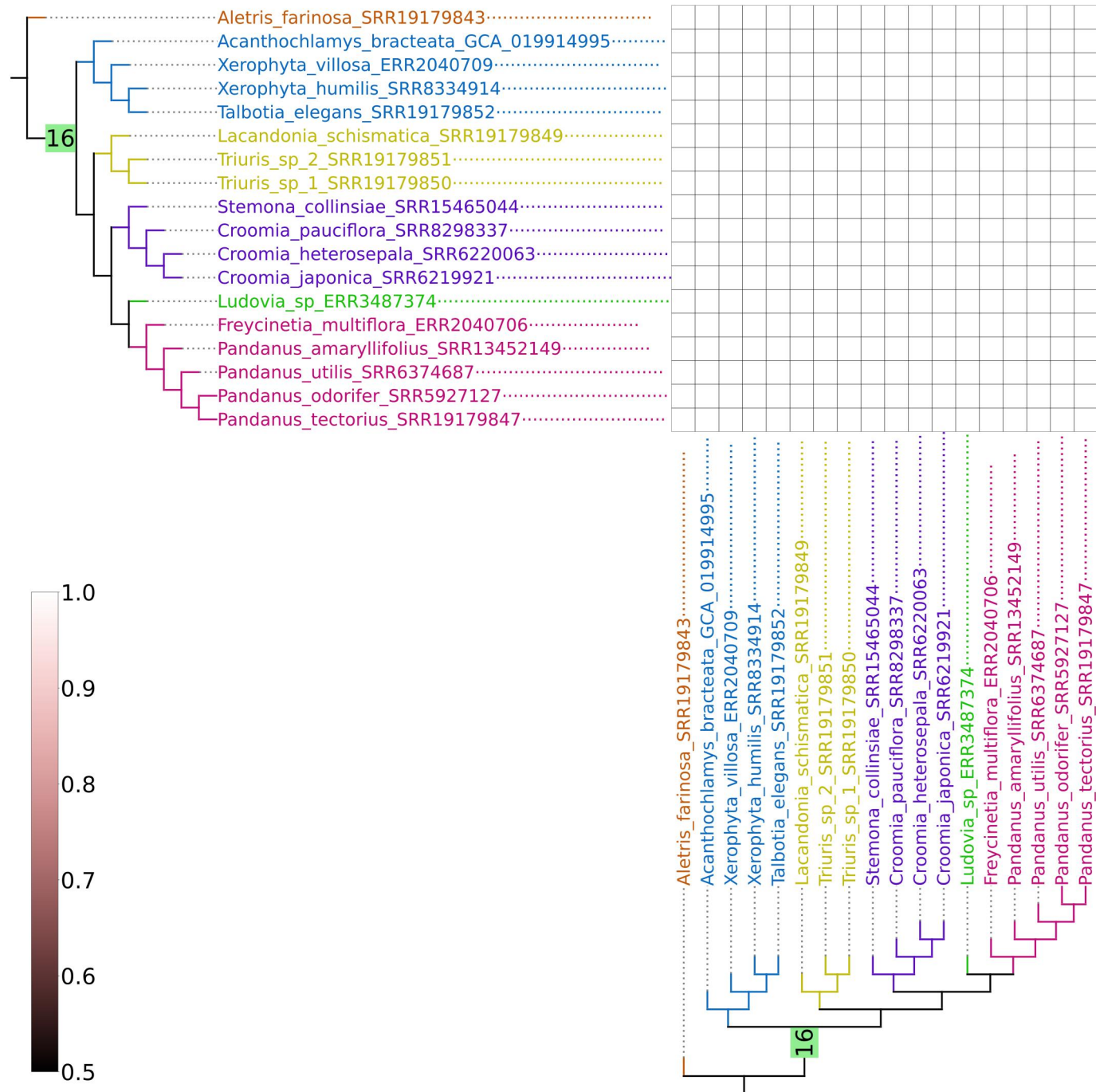

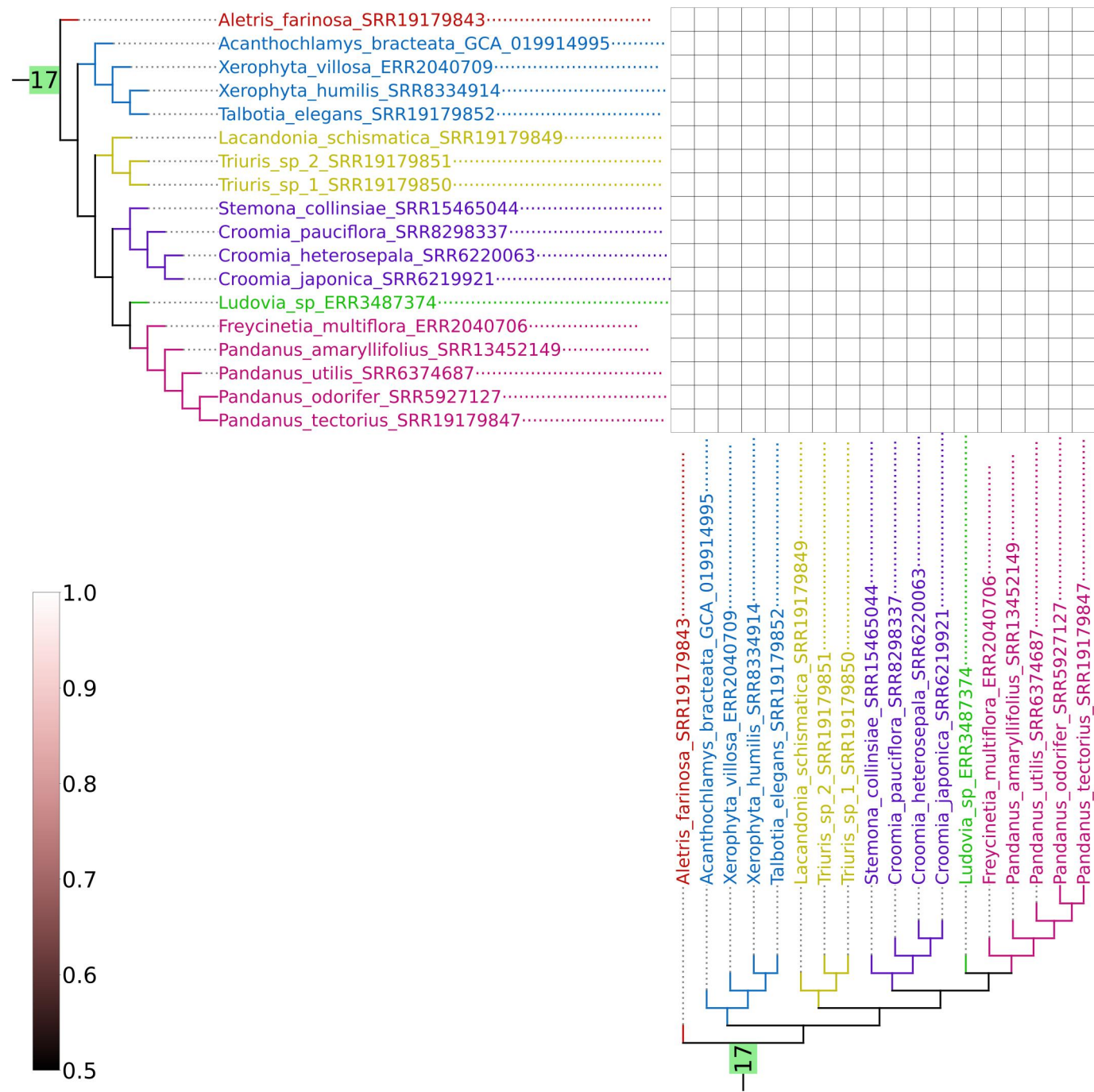

Supplement: Supplementary Figure 7 — Gene flow detection based on the results of HyDe, with heatmaps drawn for each internal node in the phylogeny of Pandanales(see Methods 2.7). [file Image7.pdf]

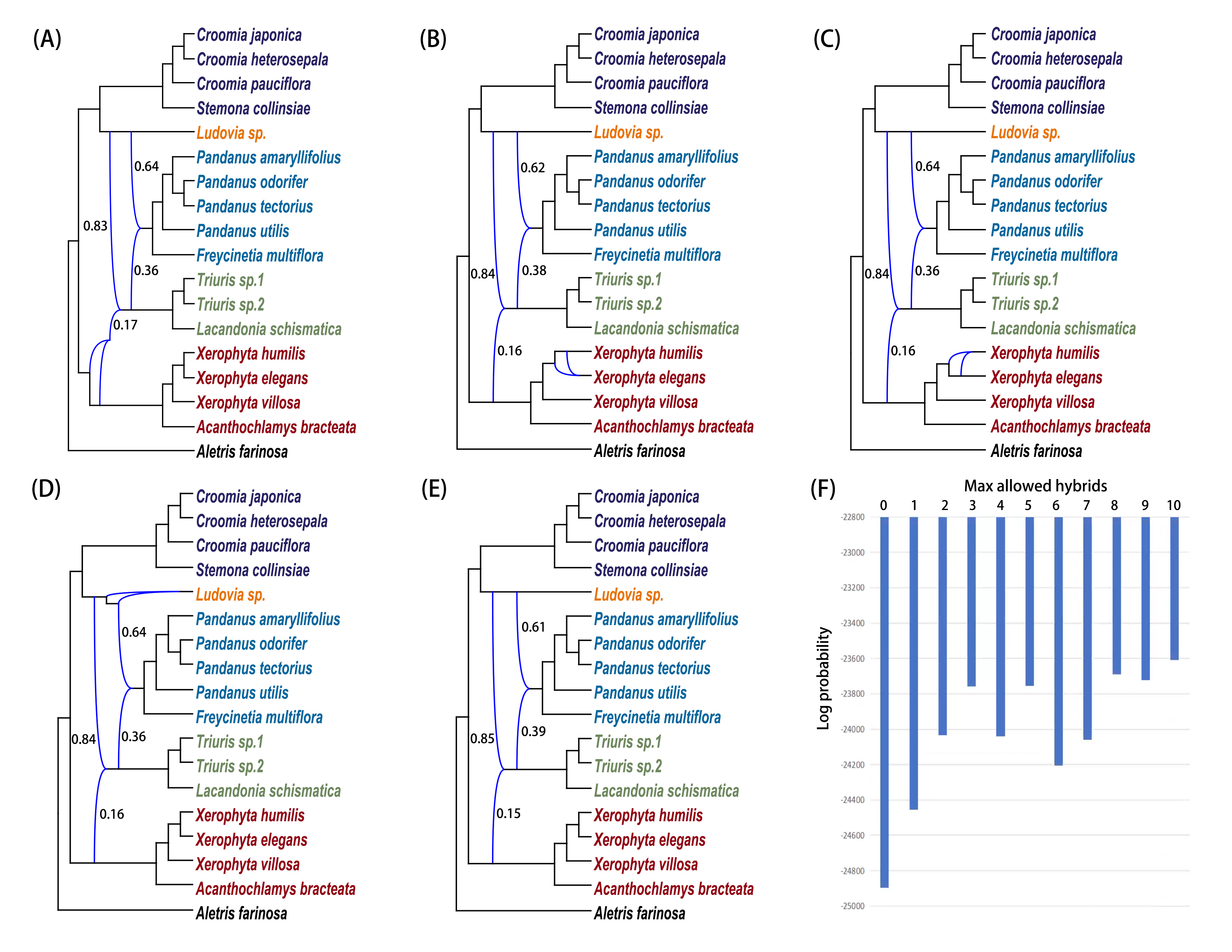

Supplement: Supplementary Figure 8 — Phylogenetic network of Pandanales constructed using single-copy orthologous genes with PhyloNet. (A-E) Phylogenetic networks generated from five independent runs where the maximum number of allowed gene flow events was set to 3. The numbers next to the curves represent inheritance values. (F) Likelihood scores for the phylogenetic networks obtained when the maximum number of gene flow events ranged from 0 to 10. [file Image8.jpeg]
